# *Nrf2* deficiency deteriorates diabetic kidney disease in *Akita* model mice

**DOI:** 10.1016/j.redox.2022.102525

**Published:** 2022-10-28

**Authors:** Yexin Liu, Akira Uruno, Ritsumi Saito, Naomi Matsukawa, Eiji Hishinuma, Daisuke Saigusa, Hong Liu, Masayuki Yamamoto

**Affiliations:** a Department of Medical Biochemistry, Tohoku University Graduate School of Medicine, Sendai, Japan; b Department of Nephrology, Blood Purification Center of the Second Xiangya Hospital, Central South University, Changsha, China; c Department of Integrative Genomics, Tohoku Medical Megabank Organization, Tohoku University, Sendai, Japan; d Advanced Research Center for Innovations in Next-Generation Medicine Tohoku University, Sendai, Japan; e Laboratory of Biomedical and Analytical Sciences, Faculty of Pharma-Science, Teikyo University, Tokyo, Japan

**Keywords:** Nrf2, Diabetic kidney disease, Oxidative stress, Inflammation, Glutathione, MALDI-MSI, DKD, diabetic kidney disease, Nrf2, NF-E2-related factor-2, Keap1, Kelch-like ECH-associated protein 1, ROS, reactive oxygen species, 8-OHdG, 8-hydroxydeoxyguanosine, MALDI-MSI, matrix-assisted laser desorption/ionization mass spectrometry imaging, NEM, *N*-ethylmaleimide, TMAO, trimethylamine *N*-oxide, IAA, indole-3-acetic acid, MCP-1, monocyte chemotactic protein-1, KIM-1, kidney injury molecule 1, NGAL, neutrophil gelatinase-associated lipocalin, TGF-β1, transforming growth factor-β1, TG, triacylglycerol, SM, sphingomyelin, CE, cholesteryl ester, LysoPC, lysophosphatidylcholine, CsMBE, CNC-sMaf binding element

## Abstract

Oxidative stress is an essential component in the progression of diabetic kidney disease (DKD), and the transcription factor NF-E2-related factor-2 (Nrf2) plays critical roles in protecting the body against oxidative stress. To clarify the roles of Nrf2 in protecting against DKD, in this study we prepared compound mutant mice with diabetes and loss of antioxidative defense. Specifically, we prepared compound *Ins2*^*Akita/+*^ (*Akita*) and *Nrf2* knockout (*Akita::Nrf2*^*−/−*^) or *Akita* and Nrf2 induction (*Akita::Keap1*^*FA/F*A^) mutant mice. Eighteen-week-old *Akita::Nrf2*^*−/−*^ mice showed more severe diabetic symptoms than *Akita* mice. In the *Akita::Nrf2*^*−/−*^ mouse kidneys, the glomeruli showed distended capillary loops, suggesting enhanced mesangiolysis. Distal tubules showed dilation and an increase in 8-hydroxydeoxyguanosine-positive staining. In the *Akita::Nrf2*^*−/−*^ mouse kidneys, the expression of glutathione (GSH) synthesis-related genes was decreased, and the actual GSH level was decreased in matrix-assisted laser desorption/ionization mass spectrometry imaging analysis. *Akita::Nrf2*^*−/−*^ mice exhibited severe inflammation and enhancement of infiltrated macrophages in the kidney*.* To further examine the progression of DKD, we compared forty-week-old *Akita* mouse kidney compounds with *Nrf2*-knockout or Nrf2 mildly induced (*Akita::Keap1*^*FA/FA*^) mice. *Nrf2*-knockout *Akita* (*Akita::Nrf2*^*−/−*^) mice displayed severe medullary cast formation, but the formation was ameliorated in *Akita::Keap1*^*FA/FA*^ mice. Moreover, in *Akita::Keap1*^*FA/FA*^ mice, tubule injury and inflammation-related gene expression were significantly suppressed, which was evident in *Akita::Nrf2*^*−/−*^ mouse kidneys. These results demonstrate that Nrf2 contributes to the protection of the kidneys against DKD by suppressing oxidative stress and inflammation.

## 1 Introduction

Diabetic nephropathy is one of the most common diseases that affects quality of life [1,2]. Thus, there is an urgent need to develop new therapeutic drugs to slow down the progression of diabetic nephropathy at an earlier stage. The term diabetic kidney disease (DKD) is now used to describe less advanced stages of renal injury in patients with diabetes mellitus [3]. The fact that current therapies related to blood pressure control and renin-angiotensin system blockade have not fully prevented the progression of DKD [4] suggests that several unneglectable factors remain in the development of active DKD [5]. To unravel the pathogenesis of DKD, oxidative stress and inflammation have been intensively studied as important mechanisms underlying the development of DKD [6,7]. In fact, the generation of oxidative stress and/or weakened antioxidative defense have been shown to coincide with the progression of DKD in patients [8]. Inflammation has also been reported to accelerate the progression of DKD, such that a parallel has been observed between persistent macrophage accumulation and the severity of DKD [9].

NF-E2-related factor 2 (Nrf2) has been shown to play essential roles in protecting against oxidative and electrophilic stresses [10,11]. Nrf2 is a transcription factor belonging to the cap-n-collar (CNC) family, which binds to CNC-sMaf binding element (CsMBE) or antioxidant/electrophile response element (ARE/EpRE) by forming a heterodimer with one of the sMaf factors [[12], [13], [14]]. Under unstressed conditions, Nrf2 is ubiquitinated by the Kelch-like ECH-associated protein 1 (Keap1)-based E3 ubiquitin ligase complex and degraded through the proteasome pathway [15]. When cells are exposed to stresses, such as reactive oxygen species (ROS) or electrophiles, reactive cysteine residues of Keap1 are modified by the stressors, and Nrf2 ubiquitination is halted [16]. Nrf2 is stabilized and translocated into the nucleus, which induces the expression of cytoprotective detoxifying and antioxidant enzymes [[17], [18], [19]]. Nrf2 also alleviates the inflammatory response by regulating the expression of proinflammatory cytokines [20]. This cytoprotective response system is referred to as the Keap1-Nrf2 system [17,21,22].

Several lines of recent evidence suggest that Nrf2 activation protects pancreatic β-cells from oxidative stress in diabetes model animals [10,23]. Genetic as well as pharmaceutical induction of Nrf2 represses the onset of diabetes in diabetes mouse models [11,[23], [24], [25]]. Therefore, the Keap1-Nrf2 system has emerged as an attractive therapeutic target in the treatment of diabetic complications. In fact, the Nrf2 inducer bardoxolone methyl has been reported to increase the glomerular filtration rate in chronic kidney disease (CKD) patients with type 2 diabetes [26].

While it has been reported that the expression of Nrf2 is upregulated in glomerular cells in the kidneys of diabetic nephropathy patients [27], it is unclear whether Nrf2 actually prevents the development of DKD. Nrf2 has been shown to protect kidneys from oxidative stress damage caused by acute renal injury (AKI), including ischemia‒reperfusion injury (IRI) and unilateral ureteral obstruction (UUO), in mouse models. In the kidneys of IRI model mice, Nrf2 induces the expression of antioxidant and NADPH synthesis enzymes and protects renal tissues [28]. Nrf2 has also been shown to ameliorate the progression of tubular damage in IRI model mice. Furthermore, *Nrf2* gene deletion aggravates fibrosis, inflammation and tubular damage after UUO [29]. However, in contrast to the situation of AKI, how Nrf2 contributes to the suppression of CKD or how loss of Nrf2 influences the development of CKD has not been examined closely. We surmise that this is partly because of the lack of optimal model mouse lines for CKD. In this regard, rodent models of streptozotocin-induced diabetes have been used to study diabetic complications. By utilizing a streptozotocin model mouse, Nrf2 was shown to suppress oxidative and nitrosative stresses and prevent DKD-like changes in the kidney [[30], [31], [32]]. However, this streptozotocin mouse model does not appear to serve as a good model system for clarifying the contribution of Nrf2 to the prevention of DKD.

Therefore, in this study, we used *Akita* mutant model mice. *Akita* mice are spontaneous diabetes model mice that carry a Cys96Tyr nonsynonymous mutation in the *Ins2* gene [33] and are known to clearly recapitulate features of early DKD [34]. This mutation induces abnormal disulfide bond formation in the insulin molecule, which generates misfolding changes and endoplasmic reticulum (ER) stress in pancreatic β-cells [35]. The mice thus show impaired insulin secretion and the development of hyperglycemia, indicating that this *Akita* mouse model is useful for studying ER stress in pancreatic β-cells [35]. As heterozygous *Ins2*^*Akita/+*^ mice display pancreatic β-cell damage in a dominant-negative manner leading to severe hyperglycemia persisting throughout the mouse life [36], *Akita* mice also provide a valuable model for studying diabetic complications. For example, *Akita* mice serve as a model of diabetic sympathetic autonomic neuropathy [37] and DKD with modest albuminuria and glomerular structural changes [38,39].

In this study, we also utilized *Nrf2*-null (*Nrf2*^*−/−*^) mice [19] and genetically Nrf2-induced mice through *Keap1* gene knockdown (*Keap1*^*FA/FA*^) [40]. To this end, *Akita* mice were crossed with *Nrf2*^*−/−*^ and *Keap1*^*FA/FA*^ mice to generate compound *Akita::Nrf2*^*−/−*^ and *Akita::Keap1*^*FA/FA*^ mutant mice, respectively. We found that *Nrf2* deficiency in the *Akita::Nrf2*^*−/−*^ mice resulted in worsened renal pathology and impaired antioxidant defense, inflammation and fibrosis in the kidneys. In contrast, Nrf2 induction in the *Akita::Keap1*^*FA/FA*^ mice ameliorated tubulointerstitial injury in the kidneys. These results revealed indispensable roles of Nrf2 in protecting against DKD development in *Akita* mice and provided lines of unequivocal evidence supporting Nrf2-targeted therapy for DKD.

## 2 Materials and methods

### 2.1 Animals

*Nrf2*^*−/−*^ and *Keap1*^*FA/FA*^ mice were previously described [19,40]. *Akita* mice (*In2*^*Akita/+*^）with the C57BL/6J background were crossed with *Nrf2*^*−/−*^ and *Keap1*^*FA/FA*^ mice with the C57BL/6J background to generate *Akita::Nrf2*^*−/−*^ and *Akita::Keap1*^*FA/FA*^ mice. Male mice were used for all experiments in this study. *Akita* mice were genotyped by PCR using the following primer sets: forward 5′-GCACAAGCGTGGCATTGTAG-3′, reverse 5′-AGCTGGTAGAGGGAGCAGAT-3′, probe *Akita* mutant 5′-FAM-ATCAGTGCTACACCAGC-3′ and probe *Akita* WT-HEX-TCAGTGCTGCACCAG-3’. These mice were raised in a specific pathogen-free animal room at Tohoku University.

The mice were housed in metabolic cages for 3 consecutive days to collect 24-h urine output; during this time, daily water intake and food consumption were recorded. The mice were fed a standard diet and given free access to water when they were in individual metabolic cages. The systolic blood pressure was measured by the tail-cuff method with a BP98A instrument (Softron).

### **2.2** Blood and urine assays

The level of blood glucose was determined using tail blood and a OneTouch UltraVue (Johnson & Johnson) or Antsense III (Horiba) glucometer. Urinary albumin was measured in the 24-h urine collections using an LBIS Mouse Albumin ELISA Kit according to the manufacturer's instructions (Fujifilm). The urine creatinine concentration of the 24-h urine was detected by using the LC‒MS/MS method, and urine osmolality was examined by a freezing point depression method. For assessment of fractional excretion of glucose, the urine glucose concentration was measured by using a Glucose Assay Kit-WST according to the manufacturer's instructions (Dojindo).

### **2.3** Histological analyses

Paraformaldehyde (PFA)-fixed and 4-μm paraffin-embedded kidney sections were stained with periodic acid-Schiff (PAS), hematoxylin and eosin (HE), and Masson's trichrome for morphological analysis. Ten cortical high-power fields (objective, 40X) with glomeruli were randomly selected when the lens was circling around the cortex area in the longitudinally sectioned kidney sections. The mesangial area, capillary loop area and tuft area of each glomerulus were measured using ImageJ software on PAS-stained slides. Cortical thickness was measured at a similar area of the longitudinally sectioned kidneys in Masson trichrome-stained sections to evaluate renal fibrosis. Kidney section slides were observed under a DM2500LED microscope (Leica).

The 4% PFA-fixed (for NQO1 and F4/80 immunostaining) and Bouin's solution-fixed (for 8-hydroxydeoxyguanosine (8-OHdG) immunostaining) kidneys were paraffin-embedded and cut into 4-μm sections before they were subjected to immunohistochemistry [41]. Deparaffined kidney sections were incubated with antibodies or immune complexes for 16 h at 4°C. Mouse monoclonal (clone Cl:A3-1) anti-rat F4/80 antibody (#MCA497GA, AbD Serotec, 5 μg/mL), a goat polyclonal anti-NQO1 antibody (#ab2346, Abcam, 1:2000), and an anti-mouse monoclonal 8-OHdG (#MOG-020P, JaICA, 2 μg/mL) conjugated with the EnVision + System- HRP Labelled Polymer (#K4000, Dako) were used. Ten random high-power fields distributed at the cortex area and outer medullary area were photographed. The percentage of the F4/80-positive area was calculated by using ImageJ software.

Kidney samples were fixed with 4% PFA for 3 h, dehydrated in gradient sucrose overnight, and embedded with Tissue-Tek OCT. Cryosections (8-μm thickness) were blocked with serum-free protein block (#X0909, Dako). The sections were then incubated overnight with a rat anti-mouse monoclonal Mac-2 antibody (#CL8942AP, Cedarlane) at a dilution of 1:1000 at 4°C. After washing with PBS, the slides were incubated with donkey anti-rat secondary antibody (Alexa Fluor 488, A-21208, Invitrogen). DAPI (4′,6-diamidino-2-phenylindole, D1306, Molecular probes) was applied to label the nucleus.

### **2.4** RT‒qPCR

Total RNA was extracted from half of the mouse kidneys homogenized in Sepasol-RNA1 Super G (Nacalai Tesque). After quantification with the NanoPhoto meter NP80 (Implen), extracted RNA was reverse transcribed into cDNA using a PrimeScript RT reagent Kit (Takara). The obtained templates were used for qPCR with the KAPA SYBR FAST qPCR master mix (2x) Kit (Kapa Biosystems) or Thunderbird qPCR Mix (Toyobo). Data were acquired by using a QuantStudio 6 Flex or a StepOnePlus Real-Time PCR system (Thermo Fisher Scientific) and normalized to the expression of the *Hprt* gene. The primer sets are described in Table S1.

### **2.5** Mass spectrometry imaging

The *in situ* GSH level was measured by matrix-assisted laser desorption/ionization mass spectrometry imaging (MALDI-MSI) analysis as previously described [11,41]. Briefly, fresh kidney samples were quickly frozen in liquid nitrogen before they were cut, and 8-μm sections were attached to indium-tin oxide slides (100 Ω/sq, Matsunami). *N*-ethyl maleimide (NEM) [42,43] was evenly sprayed on top of the sections with which GSH forms a conjugate at the thiol residue after incubation at room temperature for 1 h. The matrix, a-cyano-4-hydroxycinnamic acid (CHCA, Sigma‒Aldrich), was evaporated onto the slides at a thickness of 1.5 μm utilizing an iMLayer (Shimadzu) before MALDI-MSI analysis was performed with an iMScope (Shimadzu). The MS/MS signal of GHS-NEM was detected as *m/z* 304.1. The iMScope has an ion trap mass spectrometer. NEM-derivatized GSH was detected at *m/z* 433.1, and the transition for MS/MS analysis was *m/z* 433.1 to 304.1.

### **2.6** Plasma and urine metabolome analyses

The creatinine level was obtained by LC‒MS/MS analyses, which were performed on a UltiMate3000RSLC system coupled to a TSQ quantiva triple quadrupole mass spectrometer (Thermo Fisher Scientific). A Scherzo SS-C18 column (2 mm i.d. X50 mm, 3 μm, Imtakt) was utilized for chromatographic separation. Sample preparation was optimized from the process described previously [44]. Briefly, 10 μL inferior vena cava plasma and urine samples were well mixed with the internal standard Cre-D3 (Toronto Research Chemicals) in acetonitrile, during which the sample was deproteinized spontaneously. After homogenization and centrifugation, 80 μL of supernatant was transferred to a new tube before it was evaporated in a vacuum concentrator for 30 min. Then, the dried samples were reconstituted in 60 μL of 30% acetonitrile. After sonication and centrifugation, the sample was used for LC‒MS/MS analyses.

Metabolome analyses of inferior vena cava plasma samples were performed by using the MxP Quant 500 Kit (Biocrates) according to the manufacturer's instructions [45,46]. Five microliter samples were used for ultrahigh-performance liquid chromatography triple quadrupole MS/MS analyses (Xevo TQ-S system, Waters).

### **2.7** Statistical analyses

The data are displayed as the mean ± standard deviation (SD). For multiple comparisons, two-way analysis of variance (ANOVA) followed by Fisher's least difference test was performed. Multivariate analyses of correlations were performed using JMP Pro software (Ver. 16.1.0). The correlations were determined by Pearson's method. Differences with *P* < 0.05 were considered significant.

## 3 Results

### **3.1** Generation of Nrf2-deficient *Akita* mice

**Fig. 1:**
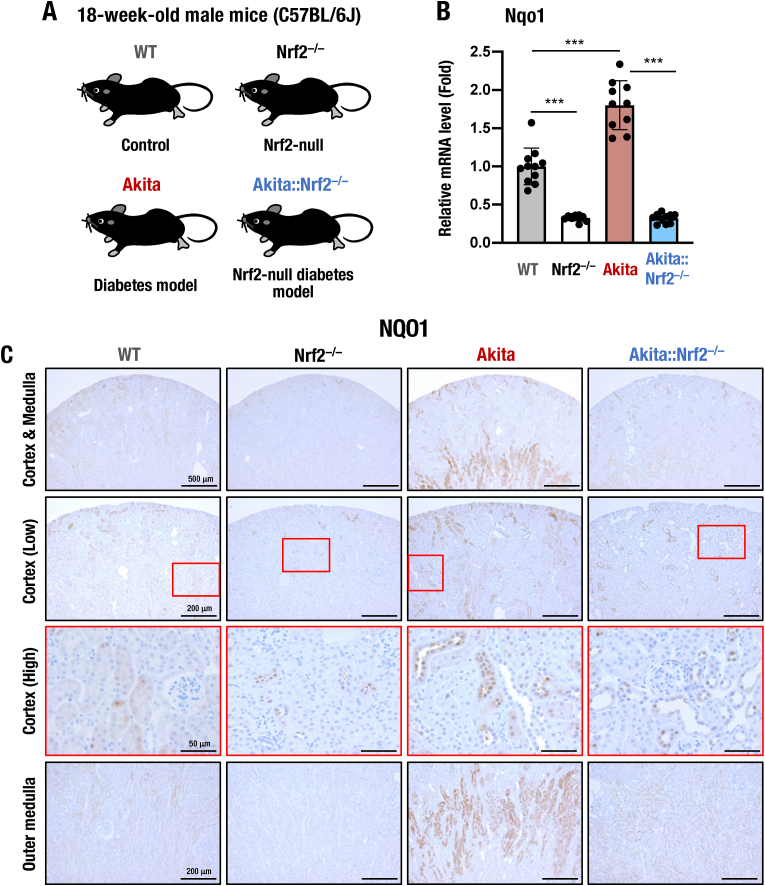
Expression of NQO1 in 18-week-old *Akita::Nrf2*^*−/−*^ mice. (A) Schematic presentation of WT, *Nrf2*^*−/−*^, *Akita* and *Akita::Nrf2*^*−/−*^ mice. Eighteen-week-old male mice were used in this study. (B) Expression of *Nqo1* mRNA in the kidneys of 18-week-old male WT, *Nrf2*^*−/−*^, *Akita* and *Akita::Nrf2*^*−/−*^ mice. The expression level was normalized to *Hprt*, and that in WT mice was set as 1. The results are presented as the mean ± SD. Statistical analyses were performed using ANOVA followed by Fisher's LSD post hoc test. (C) Immunochemistry of NQO1 in kidney sections from 18-week-old male mice. Upper images, low-magnification images including cortex and medulla. The two middle images correspond to lower- and higher-magnification images of the cortex. Lower images show the outer medulla. Cortex images are demonstrated by red squares. *Bars*, 500 μm (Cortex & Medulla), 200 μm (Cortex Low and Outer medulla) and 50 μm (Cortex High). ***P < 0.001. (For interpretation of the references to color in this figure legend, the reader is referred to the Web version of this article.)

To assess the role Nrf2 plays in response to increased blood glucose, we crossed C57BL/6J background *Nrf2*-knockout (*Nrf2*^*−/−*^) mice with heterozygous *Ins2*^*Akita*^ (abbreviated here as *Akita*) mice to generate *Nrf2*-deficient diabetic *Akita::Nrf2*^*−/−*^ mice. As 18-week-old C57BL/6J background *Akita* mice do not display overt DKD [38], we challenged to determine how *Nrf2* depletion aggravates early diabetic kidney change in these mice. To this end, four genotypes of mice, namely, WT, *Nrf2*^*−/−*^, *Akita* and *Akita::Nrf2*^*−/−*^mice, were generated for analysis (Fig. 1A).

We assessed the expression level of the Nrf2 target *Nqo1* gene to evaluate the activity of Nrf2 signaling in the kidneys of the generated *Akita* mice lacking Nrf2 at 18 weeks of age. We found that the *Nqo1* mRNA expression level was 1.8-fold higher in the kidneys of 18-week-old *Akita* mice than in those of WT mice (Fig. 1B). The expression level was markedly reduced in the kidneys of *Nrf2*^*−/−*^ and *Akita::Nrf2*^*−/−*^ mice compared to those of WT and *Akita* mice.

We next performed NQO1 immunostaining of kidney sections (Fig. 1C). Importantly, NQO1 expression was markedly increased in the kidneys of *Akita* mice compared to those of WT mice. The expression was predominantly elevated in tubular cells located at the outer medulla followed by the distal part of tubules in the cortex area but was not substantially elevated in the glomerular area. Consistent with the mRNA content results, NQO1 expression was markedly decreased in the kidney sections of *Nrf2*^*−/−*^ mice compared to those of WT mice. Immunostaining for NQO1 was also decreased in the kidney sections of *Akita::Nrf2*^*−/−*^ mice. These results indicate that Nrf2 signaling is induced in *Akita* mouse kidneys and strongly suppressed by *Nrf2* gene knockout.

### 3.2 Oxidative stress and glutathione levels in *Akita* mouse kidneys

**Fig. 2:**
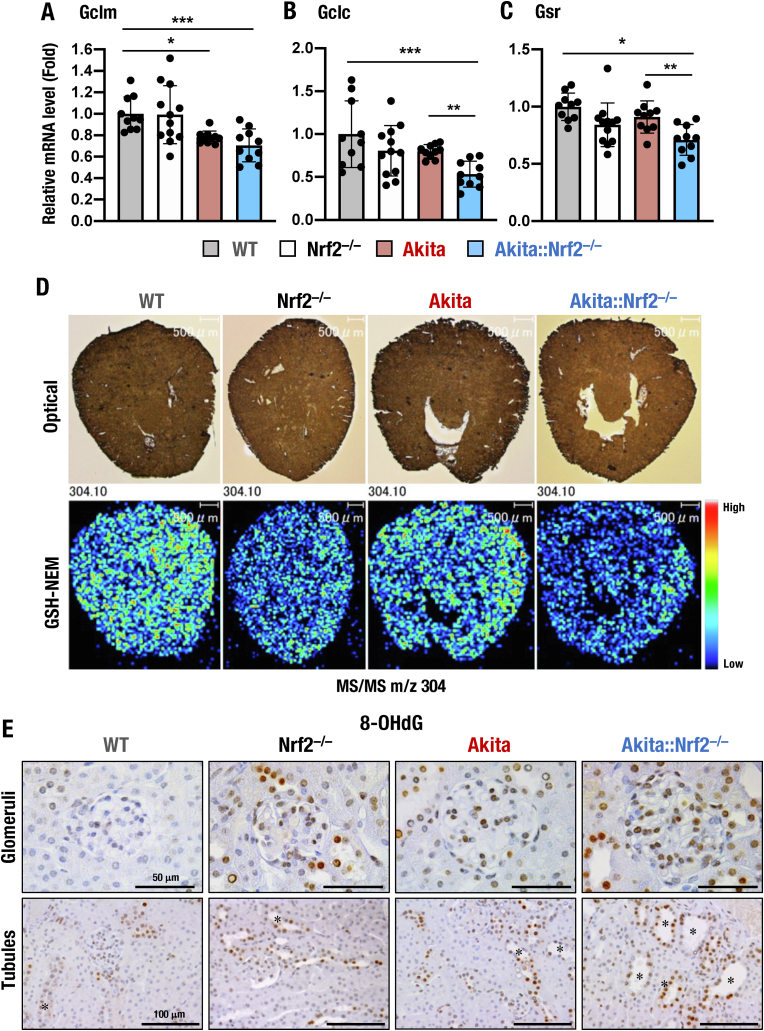
Oxidative stress and GSH levels in the kidneys of *Akita::Nrf2*^*−/−*^ mice. (A–C) mRNA expression levels of GSH synthesis-related genes in the kidneys of 18-week-old male WT, *Nrf2*^*−/−*^, *Akita* and *Akita::Nrf2*^*−/−*^ mice. The expression levels of the *Gclm* (A), *Gclc* (B) and *Gsr* (C) genes were normalized to *Hprt* expression and quantified as the fold increase relative to those of WT mice, which were set as 1. The results are presented as the mean ± SD. Statistical analyses were performed using ANOVA followed by Fisher's LSD post hoc test. *P < 0.05, **P < 0.01 and ***P < 0.001. (D) MALDI-MSI for GSH in kidney sections from 18-week-old male WT, *Nrf2*^*−/−*^, *Akita* and *Akita::Nrf2*^*−/−*^ mice. GSH was conjugated with NEM to generate GSH-NEM on the kidney sections. The MS/MS signals of GHS-NEM were detected as *m/z* 304.1. Optical images (upper panel) and MS/MS images of GSH-NEM signals (lower panel) of kidney sections. (E) Immunohistochemical staining of the oxidative stress marker 8-OHdG in the glomeruli (upper panels) and tubules (lower panel) of kidney sections. Images from each group of 18-week-old male mice are shown. Bars, 50 μm (upper panels) and 100 μm (lower panels). *P < 0.05, **P < 0.01 and ***P < 0.001.

As glutathione plays a critical role in the antioxidant response, helping to protect against excessive ROS production [47], a decrease in the reduced glutathione (GSH) level impairs the effectiveness of antioxidant defenses and could be used as an indicator of advanced oxidative stress conditions. To clarify how Nrf2 contributes to the regulation of GSH levels in DKD, we examined the expression of glutathione-related Nrf2 target genes [21,48,49]. Specifically, we analyzed the expression levels of *Gclm*, *Gclc* (encoding glutamate-cysteine ligase) and *Gsr* (glutathione reductase) and found that *Gclm* mRNA levels in the kidneys were significantly lower in *Akita* mice than in WT mice, while they were mildly lower in *Akita::Nrf2*^*−/−*^ mice than in *Akita* mice (Fig. 2A). The mRNA levels of *Gclc* and *Gsr* in the kidneys were comparable among WT, *Nrf2*^*−/−*^ and *Akita* mice but significantly lower in *Akita::Nrf2*^*−/−*^ mice than in mice of the other three genotypes (Fig. 2B and C). These results indicate that Nrf2 regulates the expression of glutathione-related Nrf2 target genes in the kidneys.

GSH is known to be differentially distributed within the kidneys of disease model mice [28]. Thus, we next explored how the distributions and levels of GSH change within kidneys by using matrix-assisted laser desorption/ionization mass spectrometry imaging (MALDI-MSI). As GSH is highly reactive, we utilized *N*-ethylmaleimide (NEM) to conjugate the thiol residue of GSH to form GSH-NEM. This method has previously been used to stabilize GSH [[41], [42], [43]]. The MS/MS signal of GSH-NEM in the kidneys was detected at *m/z* 304 and was observed throughout the kidneys (Fig. 2D). By integrating the spectra and spatial information, we visualized the abundance and distribution of GSH-NEM in kidney sections *in situ*.

The signals denoting GSH-NEM were lower in the kidneys of *Nrf2*^*−/−*^ mice than in those of WT mice (55.6 ± 5.1% of WT) and were decreased in the kidneys of *Akita* mice (73.3 ± 18.8% of WT). The signals were decreased most significantly in the kidneys of *Akita::Nrf2*^*−/−*^ mice compared to those of the other three mouse genotypes (40.6 ± 9.0% of WT, Fig. 2D).

We also evaluated the level of oxidative stress under diabetic conditions among the mouse kidneys by immunohistochemistry of 8-OHdG, an oxidative stress marker. We found enhancement of 8-OHdG staining in *Nrf2*^*−/−*^ mouse kidney compared with WT mouse kidney. In contrast, the kidneys of *Akita* mice displayed only a mild increase in 8-OHdG staining. 8-OHdG staining was dramatically elevated in the glomeruli and tubules of the kidneys in *Akita::Nrf2*^*−/−*^ mice (Fig. 2E). Taken together, these data demonstrate that the *Akita::Nrf2*^*−/−*^ compound mutant mice are more susceptible to the ROS-induced renal damage provoked in the diabetic condition than simple *Akita* mice or *Nrf2*^*−/−*^ mice.

### **3.3** *Akita::Nrf2*^−/−^ mice display severe diabetes symptoms

**Fig. 3:**
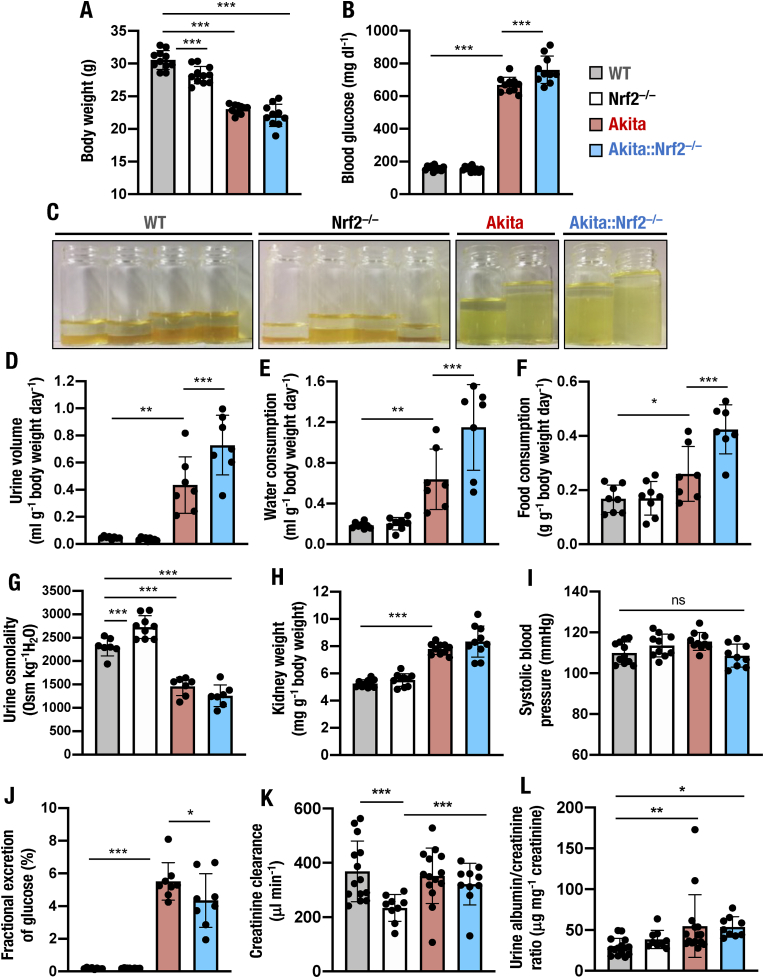
Severe diabetes symptoms of *Akita::Nrf2*^*−/−*^ mice. Body weights (A) and blood glucose levels (B) of 18-week-old male WT, *Nrf2*^*−/−*^, *Akita* and *Akita::Nrf2*^*−/−*^ mice. (C–L) Diabetes symptoms and urine osmolality of 18-week-old male WT, *Nrf2*^*−/−*^, *Akita* and *Akita::Nrf2*^*−/−*^ mice observed in metabolic cages. Representative appearance of 24-h urine output (C), 24-h urine volume (D), water consumption (E), food consumption (F) and urine osmolality (G), left kidney weight normalized by body weight (H), systolic blood pressure (I), fractional excretion of glucose (J), creatinine clearance (K) and urine albumin/creatinine ratio in the 24-h urine (L). The results are presented as the mean ± SD. Statistical analyses were performed using ANOVA followed by Fisher's LSD post hoc test (A, B, D-L). *P < 0.05, **P < 0.01 and ***P < 0.001.

For the physical examination related to diabetes mellitus, we first measured body weight. We found that the body weights of *Nrf2*^*−/−*^ mice were mildly lower than those of WT mice, and the body weights of *Akita* and *Akita::Nrf2*^*−/−*^ mice were decreased further compared to those of WT and *Nrf2*^*−/−*^ mice (Fig. 3A). The body weights were comparable between *Akita* and *Akita::Nrf2*^*−/−*^ mice. We also measured blood glucose levels. Those of *Akita* and *Akita::Nrf2*^*−/−*^ mice were elevated compared to those of WT mice (Fig. 3B). The blood glucose level of *Akita::Nrf2*^*−/−*^ mice was slightly higher than that of *Akita* mice.

To precisely examine the diabetic symptoms, we next held the mice in metabolic cages. We found that the *Akita* mice displayed an excessive volume of urine excretion, along with an increase in consumption of water and food (Fig. 3C–F). Urine volume, consumption of water, and food intake were significantly higher in *Akita::Nrf2*^*−/−*^ mice than in *Akita* mice (Fig. 3D–F). We also evaluated urine osmolality. We found that urine osmolality was mildly increased in *Nrf2*^*−/−*^ mice compared with WT mice, but urine osmolality was decreased significantly in *Akita* and *Akita::Nrf2*^*−/−*^ mice compared to WT and *Nrf2*^*−/−*^ mice (Fig. 3G). The osmolality of *Akita::Nrf2*^*−/−*^ mice was mildly decreased compared to that of *Akita* mice. These results indicate that *Akita* mice developed robust diabetes mellitus at 18 weeks of age and that *Akita::Nrf2*^*−/−*^ mice display more severe diabetic symptoms than *Akita* mice.

Of note, we found that the kidney weight was elevated in *Akita* mice compared with WT mice (Fig. 3H). The weight was comparable between *Akita* and *Akita::Nrf2*^*−/−*^ mice and between WT and *Nrf2*^*−/−*^ mice, demonstrating the presence of hyperfiltration in *Akita* and *Akita::Nrf2*^*−/−*^ mice. We next measured blood pressure and found that systolic blood pressure tended to be greater in *Akita* mice than in WT mice, while systolic blood pressure was lower in *Akita::Nrf2*^*−/−*^ mice than in *Akita* mice (Fig. 3I). We also determined the fractional excretion of glucose and found that it was dramatically elevated in *Akita* mice compared to WT and *Nrf2*^*−/−*^ mice, while it was decreased in *Akita::Nrf2*^*−/−*^ mice compared to *Akita* mice (Fig. 3J). These data indicate that *Nrf2* depletion in *Akita* mice decreases urine glucose excretion.

We next assessed the renal function of *Akita::Nrf2*^*−/−*^ mice by evaluating the creatinine clearance and albumin/creatinine ratio in 24-h urine samples. Creatinine clearance in *Nrf2*^*−/−*^ mice was lower than that in WT mice, but *Akita* and *Akita::Nrf2*^*−/−*^ mice displayed creatinine clearance levels comparable to those of WT mice (Fig. 3K). The urine albumin/creatinine ratio was mildly increased in *Akita* mice compared to WT mice, and the ratio was comparable between *Akita* and *Akita::Nrf2*^*−/−*^ mice (Fig. 3L). These data indicate that depletion of *Nrf2* in 18-week-old *Akita* diabetic mice does not result in overt aggravation of renal physiological functions or albuminuria despite various pathological changes.

### **3.4** Increase in uremic toxins and oxidative stress-related metabolite in *Akita::Nrf2*^−/−^ mice

**Fig. 4:**
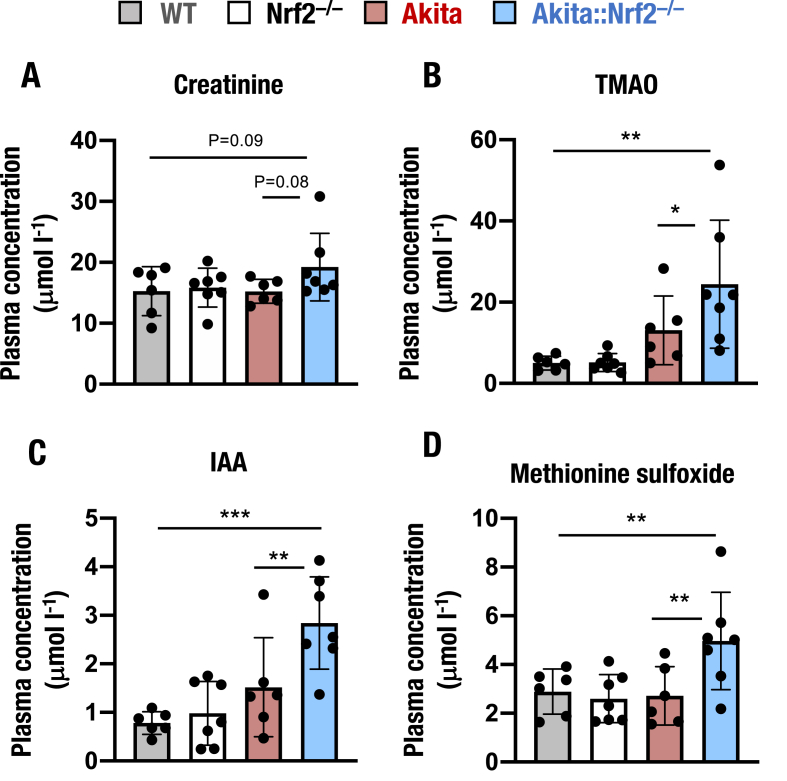
Plasma levels of uremic toxins and oxidative stress-related metabolites. Plasma levels of creatinine (A), trimethylamine *N*-oxide (TMAO, B), indole-3-acetic acid (IAA, C) and methionine sulfoxide (D) in 18-week-old *Akita::Nrf2*^*−/−*^ mice. The results are presented as the mean ± SD. Statistical analyses were performed using ANOVA followed by Fisher's LSD post hoc test. *P < 0.05, **P < 0.01 and ***P < 0.001.

Thus far, we identified that, despite increases of various diabetic markers, the *Nrf2* knockout in *Akita* mice did not severely affect kidney function until 18-weeks of age. Therefore, to assess the development of DKD in *Akita* mice and the potential influence of *Nrf2* deficiency on the disease, we extended the metabolomic analyses by using Biocrates MxP Quant 500. In an initial inspection of the metabolome data, we found that plasma creatinine levels were mildly elevated in *Akita::Nrf2*^*−/−*^ mice, but there were no significant differences in the levels among all four genotype groups (WT, *Nrf2*^*−/−*^, *Akita* and *Akita::Nrf2*^*−/−*^; Fig. 4A).

We then examined changes in uremic toxins and oxidative stress-related markers. As trimethylamine *N*-oxide (TMAO) are shown to be elevated in CKD cases [50], we measured the plasma level of TMAO in these four genotype mice. The plasma TMAO levels were significantly elevated in *Akita::Nrf2*^*−/−*^ mice compared to *Akita* and WT mice (Fig. 4B). Plasma level of another uremic toxin, indole-3-acetic acid (IAA) levels [51] was also elevated in *Akita::Nrf2*^*−/−*^ mice compared to *Akita* and WT mice (Fig. 4C). Methionine sulfoxide is known to be generated by the oxidation of methionine and has been used as a useful oxidative stress marker [52]. We found that plasma level of methionine sulfoxide was comparable among WT, *Nrf2*^*−/−*^ and *Akita* mice (Fig. 4D), but the level was elevated significantly in *Akita::Nrf2*^*−/−*^ mice.

To further evaluate the relationships of these uremic toxins with creatinine, we analyzed the correlation between creatinine and TMAO, IAA or methionine sulfoxide. The plasma levels of TMAO and IAA showed significant positive correlations with the creatinine level (Figs. S1A and B, respectively). In contrast, methionine sulfoxide did not show a significant correlation with the plasma creatinine level (Fig. S1C). These observations indicated that while the creatinine level was changed only mildly in the *Akita::Nrf2*^*−/−*^ mice, the uremic toxin levels were well correlated with the plasma creatinine level. This finding raises an intriguing possibility: that despite the mild changes in the plasma creatinine level, subclinical damage to renal function may be ongoing in *Akita::Nrf2*^*−/−*^ mice.

### **3.5** *Nrf2* deficiency triggers severe inflammation in *Akita* mouse kidneys

**Fig. 5:**
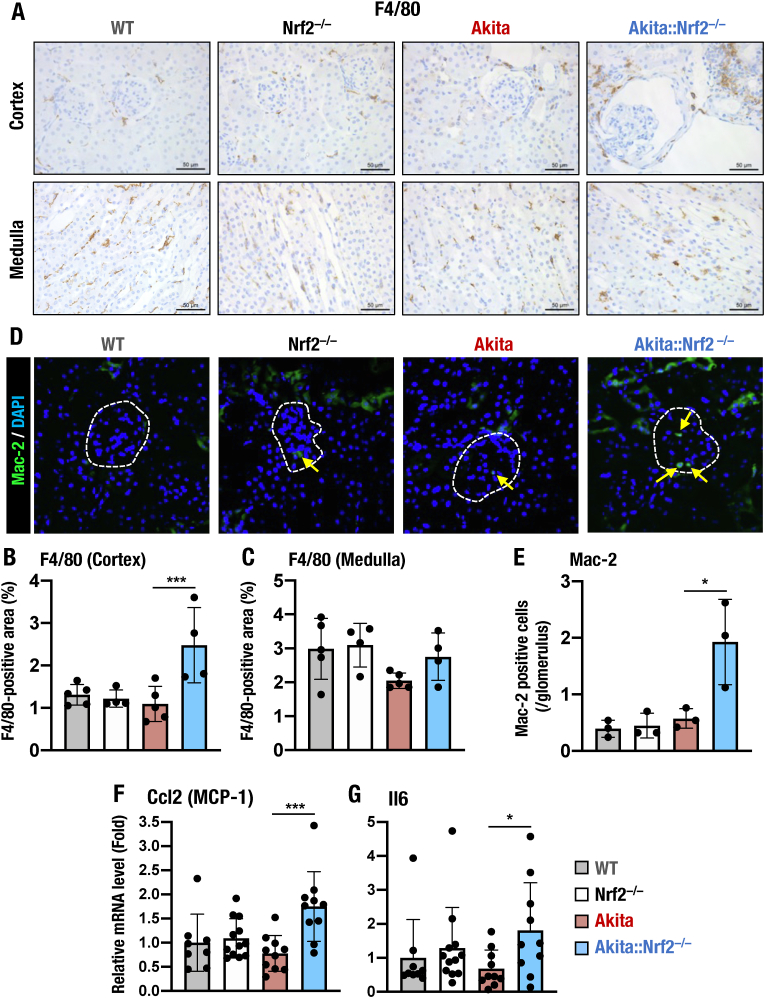
Nrf2 deficiency sensitizes *Akita* mice to severe renal inflammation. (A–C) Expression of the macrophage marker F4/80 in the kidneys of 18-week-old male WT, *Nrf2*^*−/−*^, *Akita* and *Akita::Nrf2*^*−/−*^ mice. Immunochemistry images of F4/80 (A) and quantitative analysis of the F4/80-positive area within the cortex (B) and medulla (C). (D and E) Expression of another macrophage marker, Mac-2, in the kidneys of 18-week-old male WT, *Nrf2*^*−/−*^, *Akita* and *Akita::Nrf2*^*−/−*^ mice. Immunofluorescence of Mac-2 (D) and quantification of Mac-2-positive cells (E) were performed to assess glomerular infiltrating macrophages. Yellow arrows within the glomerular region (white dashed line) indicate Mac-2-positive cells. (F and G) mRNA expression levels of the chemokine *Ccl2* (F) and the proinflammatory cytokine *Il6* (G) in the kidneys of 18-week-old male WT, *Nrf2*^*−/−*^, *Akita* and *Akita::Nrf2*^*−/−*^ mice. The expression level was normalized to *Hprt* expression. Relative mRNA levels were quantified as the fold increase relative to those of WT mice, which were set as 1. The results are presented as the mean ± SD. Statistical analyses were performed using ANOVA followed by Fisher's LSD post hoc test. *P < 0.05 and ***P < 0.001. (For interpretation of the references to color in this figure legend, the reader is referred to the Web version of this article.)

Inflammation has an important role in the pathogenesis of type 2 diabetes and DKD [53,54]. Nrf2 has been shown to exhibit inhibitory effects on inflammation by influencing macrophages and proinflammatory cytokines [20]. To clarify how Nrf2 is involved in the inflammatory response in DKD, we first examined macrophage infiltration in *Akita* and *Akita::Nrf2*^*−/−*^ mice. When assessed by F4/80 staining, macrophage infiltration was found to be increased significantly in the cortex of *Akita::Nrf2*^*−/−*^ mice, but this increase was not as significant in the outer medulla of *Akita::Nrf2*^*−/−*^ mice compared with *Nrf2*^*−/−*^ and *Akita* mice (Fig. 5A). We quantified the F4/80-positive area and found that it was indeed elevated in the cortex but not in the outer medulla of *Akita::Nrf2*^*−/−*^ mice (Fig. 5B and C).

As a marker for mature proinflammatory macrophages, F4/80 staining can only detect interstitial macrophages/monocytes. Therefore, to assess macrophage infiltration into the glomerulus, we exploited the alternative macrophage/monocyte marker Mac-2 [55,56]. Immunofluorescent staining of Mac-2 revealed that the number of infiltrating Mac-2-positive cells was comparable in the kidneys of WT, *Nrf2*^*−/−*^ and *Akita* mice. In contrast, Mac-2-positive macrophages/monocytes were considerably increased in the kidneys of *Akita::Nrf2*^*−/−*^ mice (Fig. 5D and E).

We also analyzed the expression of chemokine and proinflammatory cytokine genes, which are important for macrophage infiltration in mouse kidneys. We found that the expression levels of the *Ccl2* (encoding monocyte chemotactic protein-1, MCP-1) and *Il6* (encoding interleukin-6, IL-6) genes were increased in the kidneys of *Akita::Nrf2*^*−/−*^ mice compared with the expression in the kidneys of WT, *Nrf2*^*−/−*^ and *Akita* mice (Fig. 5F and G), consistent with the results of infiltrating macrophages. Taken together, these results demonstrate that DKD triggered in *Akita* mice is protected by and large by Nrf2, but the lack of Nrf2 sensitizes the kidney to severe inflammation, and the kidney develops overt DKD.

### **3.6** *Nrf2* ablation leads to renal fibrosis development in the kidneys of *Akita* mice

**Fig. 6:**
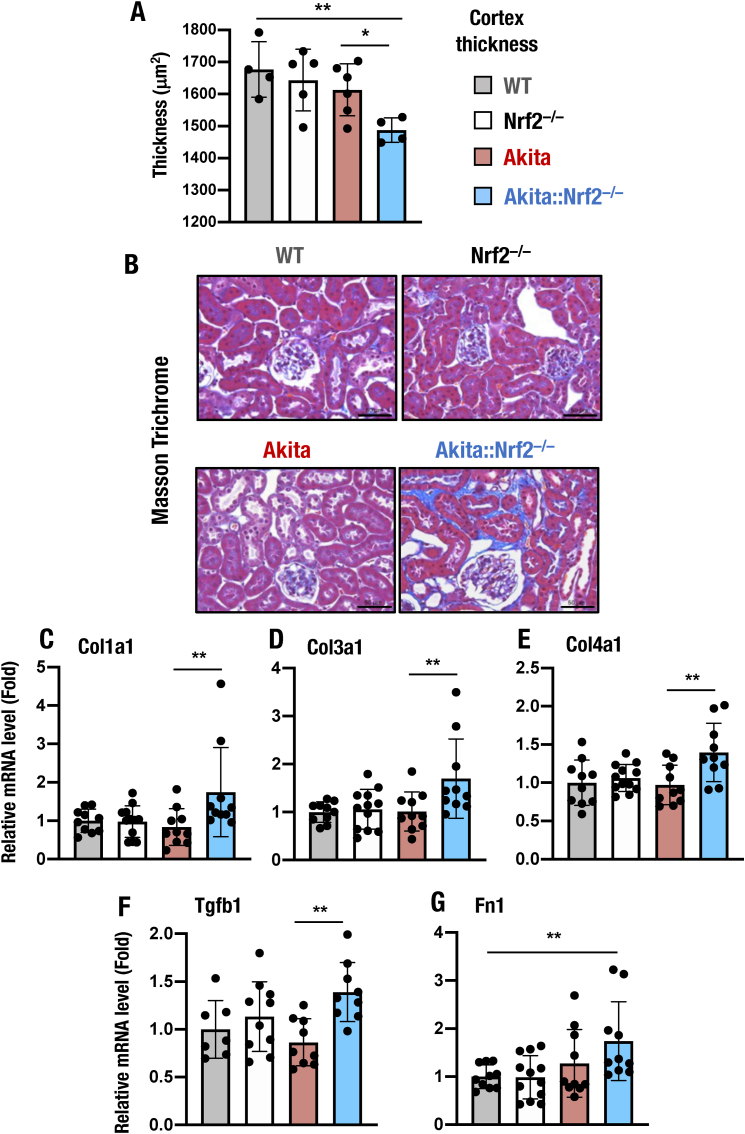
Development of fibrosis in the kidneys of *Akita::Nrf2*^*−/−*^ mice. (A) Quantification of cortex thickness in Masson's trichrome-stained specimens from 18-week-old male WT, *Nrf2*^*−/−*^, *Akita*, and *Akita::Nrf2*^*−/−*^ mice. (B) Representative images of Masson's trichrome-stained specimens from 18-week-old male WT, *Nrf2*^*−/−*^, *Akita* and *Akita::Nrf2*^*−/−*^ mice. *Bars*, 50 μm. (C–G) Expression levels of fibrogenic *Col1a1* (C), *Col3a1* (D), *Col4a1* (E), *Tgfb1* (F) and *Fn1* (G) mRNAs. Expression levels were normalized by *Hprt* and quantified as the fold increase relative to those of WT mice, which were set as 1. The results are presented as the mean ± SD. Statistical analyses were performed using ANOVA followed by Fisher's LSD post hoc test. **P < 0.01.

A reduction in renal cortical thickness emerges along with the progression of CKD [57]. The cortical thickness of the kidney has been shown to be related to the degree of renal impairment in patients with CKD [[57], [58], [59]]. Therefore, to assess the development of CKD in *Akita* mice and protection from the development of CKD by Nrf2, we next measured the thickness of the renal cortex by means of Masson's trichrome staining of kidney sections. We found that the cortical thickness of 18-week-old *Akita::Nrf2*^*−/−*^ mice was significantly decreased compared to that of WT, *Nrf2*^*−/−*^ and *Akita* mice (Fig. 6A).

We also assessed tubulointerstitial fibrosis, which is the common pathway leading to end-stage kidney disease and renal failure. While *Nrf2*^*−/−*^ or *Akita* mice did not display obvious signs of fibrosis or tubular atrophy in the respective kidney sections, sections from *Akita::Nrf2*^*−/−*^ mice exhibited blue-stained collagen fibers, which appeared surrounding the to-be or already atrophied tubules (Fig. 6B).

We also examined the expression of fibrosis-related genes in the kidneys of these mice. In agreement with the results of Masson's trichrome staining, the expression levels of the *Col1a1*, *Col3a1* and *Col4a1* (encoding the components Type I, III and IV collagen, respectively) genes were also prominently increased in the kidneys of *Akita::Nrf2*^*−/−*^ mice compared with those in WT, *Nrf2*^*−/−*^ and *Akita* mice (Fig. 6C–E). In addition, the expression levels of the *Tgfb1* (encoding transforming growth factor-β1, TGF-β1) and *Fn1* (encoding fibronectin) genes were increased in the kidneys of *Akita::Nrf2*^*−/−*^ mice (Fig. 6F and G).

These results demonstrate that the loss of Nrf2 facilitates the development of renal fibrosis and end-stage kidney disease in *Akita* mice. The enhanced tubulointerstitial fibrosis in *Akita::Nrf2*^*−/−*^ mice appears to be one of the common mechanisms leading to renal failure, and these results in turn support the contention that induction of Nrf2 acts to protect the kidney from diabetes-induced fibrosis and failure.

### 3.7 Renal glomerular and tubular changes in *Akita::Nrf2*^*−/−*^ mice

**Fig. 7:**
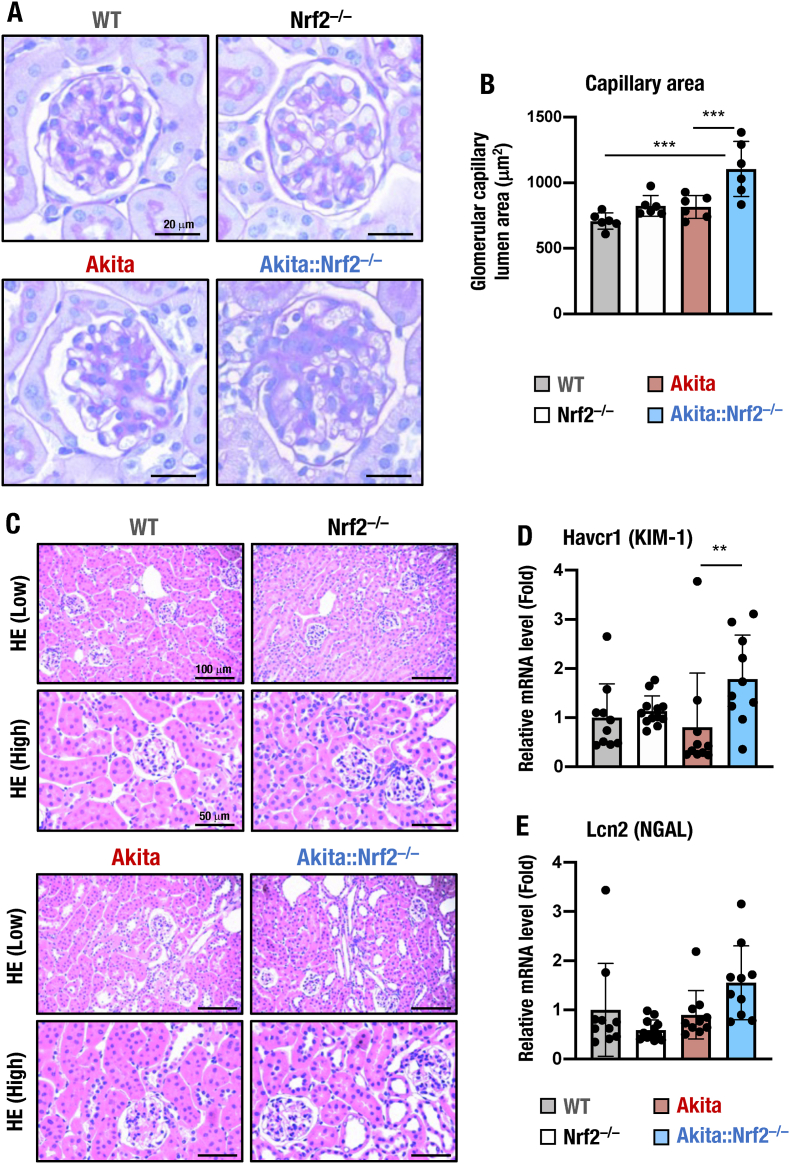
Glomerular and tubular abnormalities in *Akita::Nrf2*^*−/−*^ mouse kidneys. (A, B) PAS staining of kidney specimens from 18-week-old male WT, *Nrf2*^*−/−*^, *Akita* and *Akita::Nrf2*^*−/−*^ mice (A). Bars, 20 μm. The quantified capillary area is shown in (B). (C) Images of HE-staining of WT, *Nrf2*^*−/−*^, *Akita* and *Akita::Nrf2*^*−/−*^ mouse kidneys. Bars, 100 μm (upper panels) and 50 μm (lower panels). (D, E) The expression levels of *Havcr1* (KIM-1, D) and *Lcn2* (NGAL, E) were detected in the kidney by qPCR. mRNA levels were normalized by *Hprt* and expressed as the fold changes relative to those of WT mice, which were set as 1. The results are presented as the mean ± SD. Statistical analyses were performed using ANOVA followed by Fisher's LSD post hoc test. **P < 0.01.

To investigate how Nrf2 depletion affects kidney protection against diabetes mellitus, morphological changes in the kidneys of *Akita::Nrf2*^*−/−*^ mice were closely examined. To assess mesangial expansion in the kidneys of *Akita* and *Akita::Nrf2*^*−/−*^ mice, we stained the kidney sections with PAS. The staining demonstrated the presence of modest mesangial expansion in *Akita* mice compared with WT mice at 18 weeks of age (Fig. 7A). Notably, obvious distended capillary loops were observed in the glomeruli of *Akita::Nrf2*^*−/−*^ mice. We quantified the capillary lumen area. *Akita* mice displayed an increased capillary lumen area (Fig. 7B), which was the most prominent finding observed in the glomerulus of *Akita::Nrf2*^*−/−*^ mice, suggesting enhanced mesangiolysis [60]. Some capillary loops of *Akita::Nrf2*^*−/−*^ even dilated to the extent of a microaneurysm, which is often observed in the advanced stage of diabetic nephropathy in humans [61].

We evaluated the roles of Nrf2 in renal tubular changes during DKD. As shown in Fig. 7C, tubular morphology in HE staining of kidney sections was maintained in *Akita* and *Nrf2*^*−/−*^ mice. However, in the cortex of *Akita::Nrf2*^*−/−*^ mice, there were considerably dilated distal tubules with enlargement of tubular lumens and flattened tubular epithelial cells. The occurrence of these enlarged distal tubules may be associated with osmotic polyuria and oxidative stress-mediated injury [62,63].

We also examined functional tubular damage by analyzing the changes in the expression levels of the *Havcr1* (encoding kidney injury molecule 1, KIM-1) and *Lcn2* (encoding neutrophil gelatinase-associated lipocalin, NGAL) genes, which sensitively indicated early renal tubular injuries [64,65]. Although *Havcr1* and *Lcn2* gene expression levels were comparable between WT, *Nrf2*^*−/−*^ and *Akita* mouse kidneys, the expression levels of these genes were significantly increased in *Akita::Nrf2*^*−/−*^ mice (Fig. 7D and E). These results indicate that *Nrf2* depletion indeed worsens tubular changes in DKD.

### 3.8 Relationship between gene expression and DKD-related phenotypes

To examine how Nrf2-based gene expression regulation influences DKD-related phenotypic changes, we next analyzed the correlations between gene expression levels in kidneys and DKD-related phenotypes. We determined the mRNA expression of 16 genes in *Akita* and *Akita::Nrf2*^*−/−*^ mice, including the antioxidant and detoxification genes *Nqo1*, *Hmox1* (encoding heme oxygenase 1), *Gclc*, *Gclm* and *Gsr*; the inflammation-related genes *Il6, Il1b* (encoding interleukin-1β, IL-1β), *Ccl2* (MCP-1) and *Adgre1* (encoding F4/80); the fibrosis-related genes *Fn1* (fibronectin), *Tgfb1* (TGF-β1), *Col1a1*, *Col3a1* and *Col4a1*; and the kidney damage-related genes *Havcr1* (KIM-1) and *Lcn2* (NGAL). We performed multivariate analyses of the correlations between the expression of these genes and urine osmolarity, urine volume, kidney weight and body weight as DKD-related phenotypic parameters.

We identified urine osmolarity-associated genes and found that *Gclc* was positively correlated with osmolarity (Table S2). We next examined genes that contributed to urine volume. *Nqo1* and *Gclc* expression was negatively correlated with urine volume, while *Adgre1* and *Col4a1* gene expression was positively correlated with urine volume (Table S2). We also evaluated the relationship between kidney weight and gene expression but could not find genes that were significantly correlated with kidney weight (Table S2). We next evaluated the association of body weight and gene expression and found that *Nqo1* and *Gsr* gene expression was positively correlated with body weight, while *Ccl2* gene expression was negatively correlated with body weight (Table S2).

We then plotted the correlations between *Gclc* expression and urine osmolarity (Fig. S2A); *Gclc*, *Col4a1* and *Adgre1* expression and urine volume (Figs. S2B–D); and *Nqo1* and *Ccl2* gene expression and body weight (Figs. S2E and F). The results demonstrated a strong correlation between the expression of these genes and the phenotypic parameters, showing very good agreement with the results in Supplementary Table 2. Taken together, these data suggest that changes in oxidative stress, inflammation and fibrosis contribute to DKD-related phenotypes in *Akita::Nrf2*^*−/−*^ mice.

### 3.9 Changes in metabolic gene expression in *Akita::Nrf2*^*−/−*^ mice

**Fig. 8:**
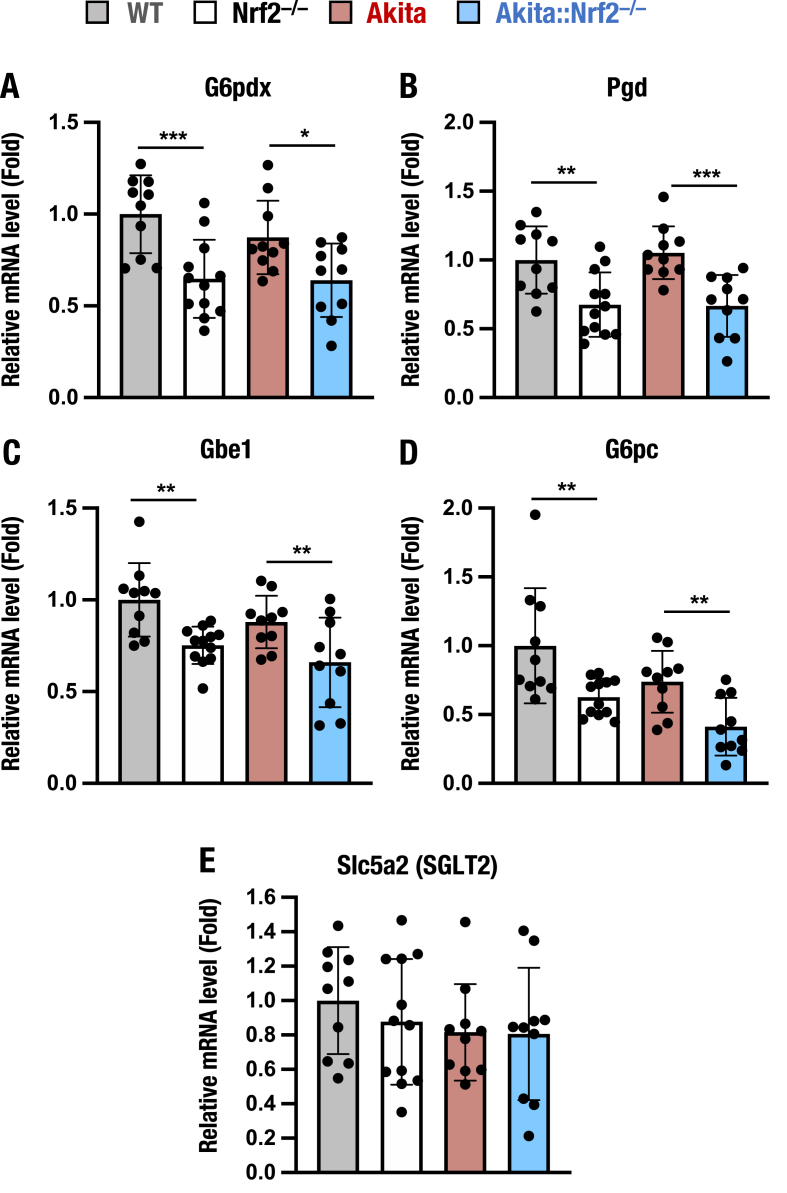
Expression of metabolic Nrf2 target genes in the kidneys of 18-week-old *Akita::Nrf2*^*−/−*^ mice. (A) mRNA expression levels of the pentose phosphate pathway-related genes *G6pdx* (A) and *Pgd* (B), the glycogen-related gene *Gbe1* (C), the gluconeogenesis-related gene *G6pc* (D) and the glucose reabsorption gene *Slc5a2* (SGLT2, E) in the kidneys of WT, *Nrf2*^*−/−*^, *Akita* and *Akita::Nrf2*^*−/−*^ mice. The expression level was normalized to that of *Hprt* and quantified as the fold increase relative to that in the WT mice, which was set as 1. The results are presented as the mean ± SD. Statistical analyses were performed using ANOVA followed by Fisher's LSD post hoc test. *P < 0.05, **P < 0.01 and ***P < 0.001.

Nrf2 is known to regulate the expression of metabolic genes and antioxidant genes [66]. We examined the expression of Nrf2 target metabolic genes, including the pentose phosphate pathway-related genes *G6pdx* (encoding glucose-6-phosphate dehydrogenase) and *Pgd* (encoding 6-phosphogluconate dehydrogenase) [28,67], the glycogen-related gene *Gbe1* (encoding glycogen branching enzyme) [25], the gluconeogenesis-related gene *G6pc* (encoding the glucose-6-phosphatase catalytic subunit) [23] and the glucose reabsorption-related gene *Slc5a2* (encoding sodium/glucose cotransporter 2, SGLT2) [68]. The expression levels of the genes *G6pdx*, *Pgd*, *Gbe1* and *G6pc* were comparable between WT and *Akita* mouse kidneys but were significantly lower in *Nrf2*^*−/−*^ and *Akita::Nrf2*^*−/−*^ mice than in WT and *Akita* mice (Fig. 8A–D).

We also determined the expression of the *Slc5a2* gene and found that the expression was comparable among these four genotypes (Fig. 8E). These results indicate that Nrf2 indeed regulates the expression of metabolic Nrf2 target genes in *Akita* mouse kidneys but that *Slc5a2*, encoding SGLT2, is not under the influence of Nrf2.

### 3.10 Detailed plasma metabolome analyses of *Akita::Nrf2*^*−/−*^ mice

Since *Nrf2* knockout in *Akita* mice affects the expression of metabolic enzyme genes, we analyzed detailed metabolic changes caused by the loss of Nrf2 in *Akita* mice. In the metabolome analyses described above, we measured the plasma levels of 624 metabolites, which enabled assessment of how Nrf2 influences metabolic regulation in *Akita* mice. In particular, we determined the total levels of triacylglycerol (TG), sphingomyelin (SM), cholesteryl ester (CE), and lysophosphatidylcholine (LysoPC).

**Fig. 9:**
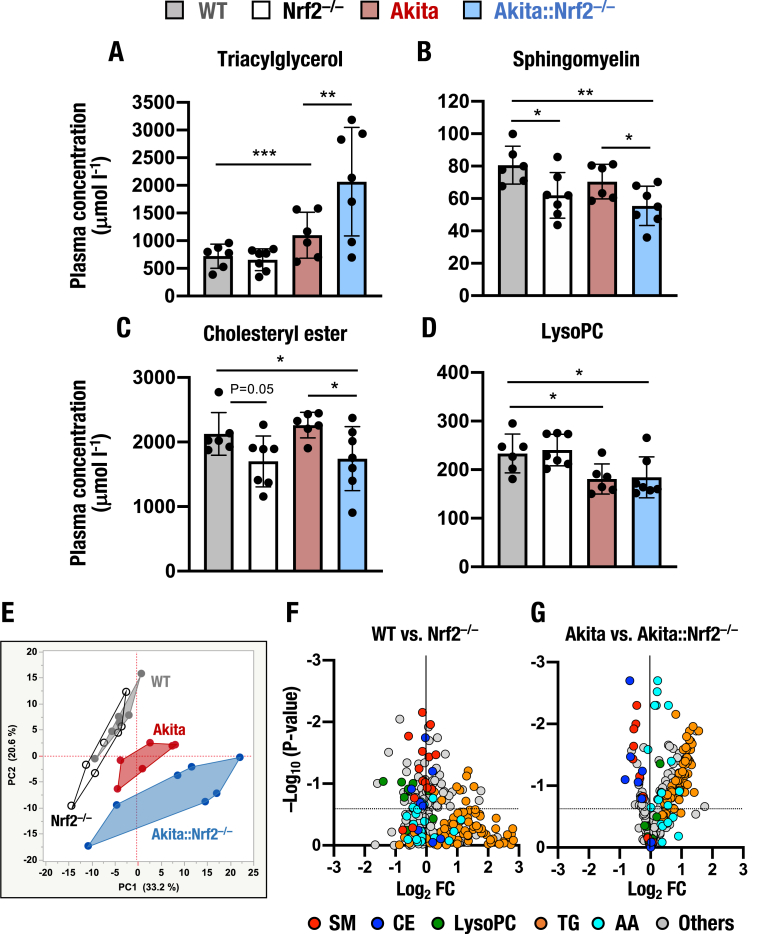
Metabolome analyses of *Akita::Nrf2*^*−/−*^ mice. (A–D) Plasma levels of triacylglycerol (TG, A), sphingomyelin (SM, B), cholesteryl ester (CE, C) and lysophosphatidylcholine (LysoPC, D). Metabolomics analyses were executed by using plasma samples from 18-week-old male WT, *Nrf2*^*−/−*^, *Akita* and *Akita::Nrf2*^*−/−*^ mice and an MxP Quant 500 Kit. The results are presented as the mean ± SD. Statistical analyses were performed using ANOVA followed by Fisher's LSD post hoc test. *P < 0.05, **P < 0.01 and ***P < 0.001. (E) Principal component analyses (PCAs) of the plasma metabolome. The 261 qualified metabolites were determined, and PCA of the plasma levels of these metabolites was performed. (F and G) Volcano plots. The X-axis is the log_2_ fold change (FC) of plasma levels, *Nrf2*^*−/−*^ compared to WT mice (G) and *Akita::Nrf2*^*−/−*^ mice compared to *Akita* mice (H). The Y-axis is the negative Log_10_ of the two-tailed test *P* value. Vertical dotted lines denote a linear fold change of one. Horizonal dotted lines indicate *P* = 0.05.

While the plasma level of TG was comparable between *Nrf2*^*−/−*^ and WT mice and mildly elevated in *Akita* mice, the TG level was significantly higher in *Akita::Nrf2*^*−/−*^ mice than in all the other genotype mice (Fig. 9A). In contrast, the plasma levels of SM and CE were decreased in *Nrf2*^*−/−*^ mice compared to WT mice (Fig. 9B and C). The plasma levels of SM and CE were lower in *Akita::Nrf2*^*−/−*^ mice than those in *Akita* mice. Furthermore, the plasma level of LysoPC was lower in *Akita* and *Akita::Nrf2*^*−/−*^ mice than in WT and *Nrf2*^*−/−*^mice, but the level was comparable between *Akita::Nrf2*^*−/−*^ and *Akita* mice (Fig. 9D).

Thus, *Nrf2* deletion alters plasma levels of TG under diabetic conditions, although it does not influence these levels under nondiabetic conditions. In contrast, *Nrf2* deletion alters the plasma levels of SM and CE in both diabetic and nondiabetic conditions. The plasma LysoPC level is influenced under diabetic conditions, and *Nrf2* deletion does not change the levels. These results demonstrate that *Nrf2* knockout elicits substantial changes in the plasma levels of various metabolites.

We then performed a multivariate analysis of the relationship between plasma levels of uremic toxins and 20 amino acids (AAs), total TG, total SM, total CE and total LysoPC in *Akita* and *Akita::Nrf2*^*−/−*^ mice. We found that plasma creatinine levels were positively correlated with the levels of 8 AAs: alanine, isoleucine, leucine, lysine, phenylalanine, proline, tyrosine, and valine (Table S3). The plasma TMAO level was positively correlated with levels of 3 branched-chain AAs (BCAAs): isoleucine, leucine and valine (Table S3). The plasma IAA level was positively correlated with the levels of 12 AAs: alanine, asparagine, glycine, histidine, lysine, methionine, phenylalanine, proline, serine, threonine, tyrosine and valine (Table S3). The plasma methionine sulfoxide level was correlated with the levels of 10 AAs, namely, alanine, arginine, asparagine, glycine, methionine, proline, serine, threonine, tyrosine, and valine, as well as with the total TG level (Table S3). These results indicate that plasma levels of uremic toxins and the oxidative stress marker methionine sulfoxide are highly correlated with those of AAs.

We then selected 261 qualified metabolites from 624 measured metabolites and executed principal component analyses (PCAs) of plasma levels of the 261 qualified metabolites in WT, *Nrf2*^*−/−*^, *Akita* and *Akita::Nrf2*^*−/−*^ mice. While components 1 and 2 (PC1 and PC2, respectively) did not separate WT and *Nrf2*^*−/−*^ mice, PC1 and PC2 of *Akita* mice were nicely separated from those of WT mice (Fig. 9E). In addition, PC2 of *Akita::Nrf2*^*−/−*^ mice was separated from that of *Akita* mice.

We next analyzed volcano plots between WT and *Nrf2*^*−/−*^ mice. The volcano plots revealed that compared to WT mice, *Nrf2*^*−/−*^ mice exhibited decreased plasma levels of SMs (Fig. 9F, red plots), CEs (blue plots) and LysoPCs (green plots). We also analyzed volcano plots between *Akita* and *Akita::Nrf2*^*−/−*^ mice, which revealed that plasma levels of SMs and CEs were potently decreased, but those of LysoPCs were not altered in *Akita::Nrf2*^*−/−*^ mice compared to *Akita* mice (Fig. 9G). Of note, the plasma levels of many TGs (orange plots) and AAs (cyan plots) were markedly higher in *Akita::Nrf2*^*−/−*^ mice than in *Akita* mice (Fig. 9G). The TGs and AAs did not change obviously in *Nrf2*^*−/−*^ mice compared to WT mice (Fig. 9F). These results indicate that *Nrf2* knockout more potently influences metabolic regulation under diabetic conditions than under nondiabetic conditions.

### 3.11 Metabolites altered plasma levels in *Akita::Nrf2*^*−/−*^ mice

**Fig. 10:**
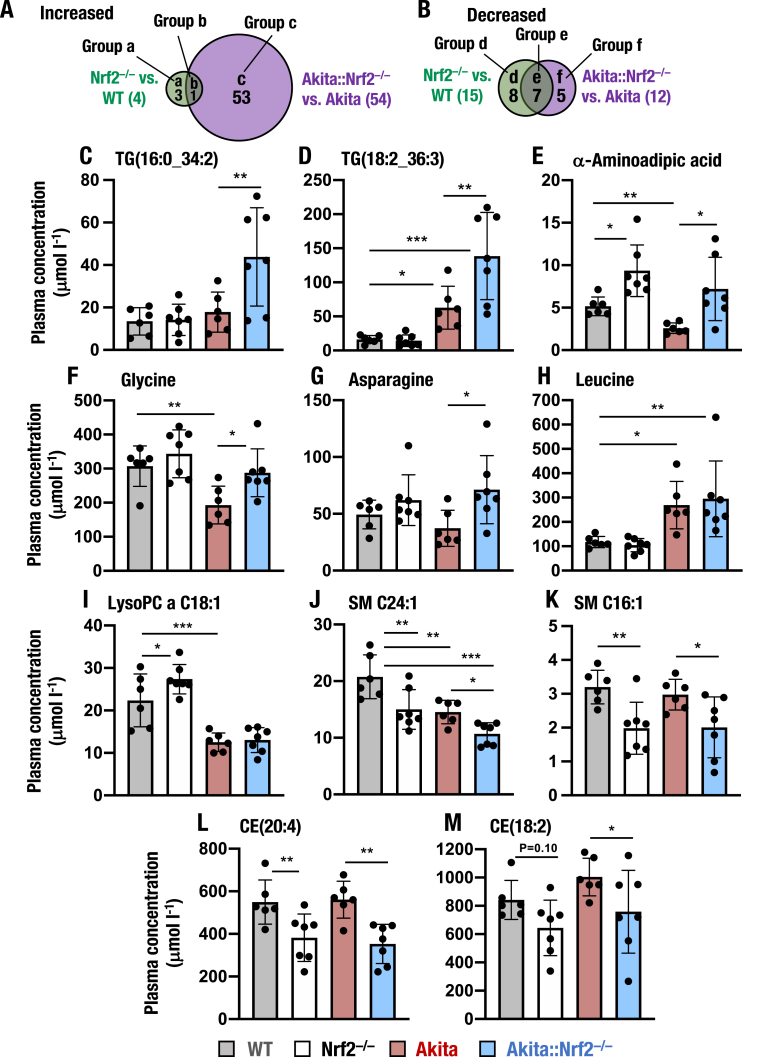
Metabolites showing altered plasma levels in *Akita::Nrf2*^*−/−*^ mice. (A and B) Numbers of metabolites whose levels in plasma are significantly increased (A) or decreased (B). Venn diagram of metabolites whose levels were altered in *Nrf2*^*−/−*^ mice compared to WT mice (green) and in *Akita::Nrf2*^*−/−*^ mice compared to *Akita* mice (purple). (C–M) Representative metabolites whose levels are changed in 18-week-old *Akita::Nrf2*^*−/−*^ mice. These metabolites include TG(16:0_34:2) (C), TG(18:2_36:3) (D), α-aminoadipic acid (E), glycine (F), asparagine (G), leucine (H), lysoPC a C18:1 (I), SM C24:1 (J), SM C16:1 (K), CE(20:4) (L) and CE(18:2) (M). The results are presented as the mean ± SD. Statistical analyses were performed using ANOVA followed by Fisher's LSD post hoc test. *P < 0.05, **P < 0.01 and ***P < 0.001. (For interpretation of the references to color in this figure legend, the reader is referred to the Web version of this article.)

To compare the effects of Nrf2 on metabolic regulation between diabetic and nondiabetic conditions, we performed comprehensive analyses of the plasma metabolite levels. To this end, we employed two-way ANOVA and a post hoc test to determine the *P* value between each genotype pair (Table S4). As we were interested in the metabolites whose levels were either increased or decreased by the loss of Nrf2, we first searched for such metabolites and expressed the results in a Venn diagram (Fig. 10A and B). We found that the plasma levels of 3 and 53 metabolites were significantly higher in *Nrf2*^*−/−*^ and *Akita::Nrf2*^*−/−*^ mice than in WT and *Akita* mice, respectively (Fig. 10A, green and purple). These metabolites were grouped into Group a and Group c, respectively. One metabolite belonged to both groups (Group b). We also found that the plasma levels of 8 and 5 metabolites were lower in *Nrf2*^*−/−*^ and *Akita::Nrf2*^*−/−*^ mice than in WT and *Akita* mice, respectively (Fig. 10B, green and purple). Seven metabolites belonged to both groups (Group e).

We then evaluated these metabolites individually. We started this inspection from triacylglycerol, which were heavily influenced by both diabetic situation and Nrf2 (categorized in Group c). While the plasma levels of TG(16:0_34:2) were comparable among WT, *Nrf2*^*−/−*^ and *Akita* mice, this triacylglycerol level was increased significantly in *Akita::Nrf2*^*−/−*^ mice compared to those of the other genotype mice (Fig. 10C). The plasma level of TG(18:2_36:3) showed similar tendency. While this triacylglycerol level was increased in *Akita* mice compared to WT mice, the elevation was significantly aggravated in *Akita::Nrf2*^*−/−*^ mice (Fig. 10D). These results showed very good coincidence with the observation that plasma levels of total TG were increased in *Akita::Nrf2*^*−/−*^ mice (Fig. 9A).

We found that α-aminoadipic acid is the only member of Group b. Plasma level of α-aminoadipic acid was markedly increased in both *Nrf2*^*−/−*^ and *Akita::Nrf2*^*−/−*^ mice compared to those in WT and *Akita* mice, respectively (Fig. 10E), indicating that *Nrf2* deficiency increases plasma α-aminoadipic acid levels in both diabetic and nondiabetic conditions.

We then examined AA levels, which showed pleiotropic responses to the diabetic and loss-of-Nrf2 situations. For example, the plasma level of glycine was lower in *Akita* mice than in WT mice, whereas the level was recovered in *Akita::Nrf2*^*−/−*^ mice (Fig. 10F). Similar to the case for TG, the plasma asparagine level was comparable among WT, *Nrf2*^*−/−*^ and *Akita* mice, but the level was increased in *Akita::Nrf2*^*−/−*^ mice (Fig. 10G). These results demonstrate that *Nrf2* deficiency drives increases in the levels of a set of AA levels in plasma, especially on the *Akita* background.

Since plasma levels of BCAAs are known to be associated with diabetes mellitus [69,70], we also evaluated BCAA levels. The plasma level of leucine was strongly increased in *Akita* mice compared to WT mice, while it was comparable between *Akita* and *Akita::Nrf2*^*−/−*^ mice and between WT and *Nrf2*^*−/−*^ mice (Fig. 10H). The plasma levels of the other BCAAs, *i.e.,* isoleucine and valine, showed similar changes (Table S4), indicating that plasma BCAA levels were increased under hyperglycemic conditions in *Akita* mice, but *Nrf2* deletion did not influence the BCAA levels.

In contrast to the metabolites examined above, plasma level of lysoPC a C18:1 was significantly decreased in *Akita* mice compared to WT mice and the levels were comparable between *Akita* and *Akita::Nrf2*^*−/−*^ mice (Fig. 10I). While this phospholipid level was increased in *Nrf2*^*−/−*^ mouse plasma, the increase was completely canceled in the *Akita* background. Similarly, plasma level of SM C24:1 was decreased in *Akita* mouse plasma compared to WT mice, and the level was further decreased in *Akita::Nrf2*^*−/−*^ mice compared to *Akita* mice (Fig. 10J). In contrast, plasma level of SM C16:1 was decreased in *Nrf2*^*−/−*^ mice compared to that of WT mice, while plasma level of this sphingomyelin did not change much in *Akita* mouse plasma compared to that of WT mice and in *Akita::Nrf2*^*−/−*^ mouse plasma compare to that of *Nrf2*^*−/−*^ mice (Fig. 10K). The plasma levels of CE(20:4) and CE(18:2) showed similar changes to that of SM C16:1 (Fig. 10L and M, respectively). These results thus demonstrate that *Nrf2* deficiency decreases plasma levels of SM C24:1, SM C16:1 and CE(20:4) in both diabetic and nondiabetic conditions (Group e), indicating that plasma levels of SMs and CEs are heavily influenced by the loss-of-Nrf2. Taken together, these results support our contention that Nrf2 acts as an important regulator of the metabolism in diabetic conditions of the Akita mouse.

### 3.12 Gene regulation in the kidneys of elderly *Akita* mice

One remaining question was whether the diabetic kidney damage in *Akita* mice becomes serious with age. To address this question, we determined the expression levels of oxidative damage-related genes in the kidneys of 40-week-old WT, *Akita* and *Akita::Nrf2*^*−/−*^ mice by qPCR and compared them with the levels in 18-week-old mice of the same genotypes.

**Fig. 11:**
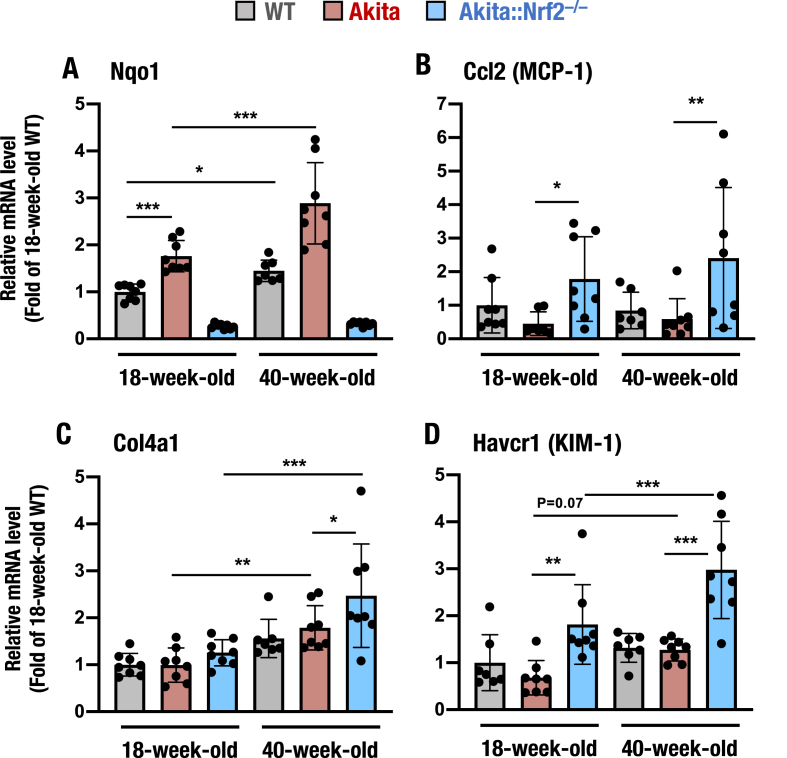
Gene expression in the kidneys of 18- and 40-week-old *Akita* and *Akita::Nrf2*^*−/−*^ mice. The mRNA expression levels of the Nrf2 target gene *Nqo1* (A), the inflammation-related gene *Ccl2* (MCP-1, B), the fibrosis-related gene *Col4a1* (C), and the kidney damage-related gene *Havcr1* (KIM-1, D) in the kidneys of WT, *Akita* and *Akita::Nrf2*^*−/−*^ mice were examined. The expression level was normalized to that of *Hprt* and quantified as the fold increase relative to that in the WT mice, which was set as 1. The results are presented as the mean ± SD. Statistical analyses were performed using ANOVA followed by Fisher's LSD post hoc test. *P < 0.05, **P < 0.01 and ***P < 0.001.

We first determined the expression of Nrf2 target genes and found that *Nqo1* expression was significantly higher in 40-week-old *Akita* mouse kidneys than in 18-week-old *Akita* mouse kidneys (Fig. 11A). Nrf2 target gene induction in *Akita* mouse kidneys compared to WT mouse kidneys nicely reproduced the results of the analysis of 18-week-old *Akita* mouse kidneys shown in Fig. 1B. This increase in *Nqo1* expression was abrogated in both 18- and 40-week-old *Akita::Nrf2*^*−/−*^ mice. These results indicate that diabetic kidney damage in *Akita* mice induces the expression of Nrf2 target genes and that the induction increases with age.

We also examined the expression of *Ccl2* (MCP-1) in the kidneys of elderly *Akita* mice. Since *Ccl2* gene expression is negatively regulated by Nrf2 and since Nrf2 expression is upregulated in *Akita* mouse kidneys, kidney *Ccl2* expression was reduced in *Akita* mice, while it was increased in both 18- and 40-week-old *Akita::Nrf2*^*−/−*^ mice (Fig. 11B). The magnitudes of suppression and induction in younger and older *Akita* mouse kidneys did not differ substantially. In contrast, the expression of the gene *Col4a1* was higher in 40-week-old *Akita* and *Akita::Nrf2*^*−/−*^ mouse kidneys than in 18-week-old *Akita* and *Akita::Nrf2*^*−/−*^ mouse kidneys (Fig. 11C).

We also measured the expression of the gene *Havcr1* (KIM-1) and found that it was elevated in both 18-week-old and 40-week-old *Akita::Nrf2*^*−/−*^ mice compared with *Akita* mice (Fig. 11D). *Havcr1* expression was markedly higher in 40-week-old *Akita::Nrf2*^*−/−*^ mice than in 18-week-old *Akita::Nrf2*^*−/−*^ mice.

To further assess the effects of *Nrf2* knockout on kidney damage with age, we analyzed the correlation between *Hrvcr1* and *Ccl2* gene expression in *Akita* and *Akita::Nrf2*^*−/−*^ mice at 18 weeks (Fig. S3A) and 40 weeks (Fig. S3B). We found that *Ccl2* expression was strongly correlated with *Hrvcr1* expression in 40-week-old *Akita::Nrf2*^*−/−*^ mice (Fig. S3B). In contrast, it was not correlated well in 18-week-old mice (Fig. S3A). The significant elevation in *Hrvcr1* gene expression in 40-week-old *Akita::Nrf2*^*−/−*^ mouse kidneys suggests that Nrf2 acts to protect kidneys against diabetic kidney damage. However, the increases in *Hrvcr1* and *Col4a1* expression in *Akita* mouse kidneys suggest that the magnitude of the increase in Nrf2 may not be enough to protect kidneys against severe diabetic damage.

### 3.13 *Nrf2* induction in elderly *Akita* mice

We finally decided to investigate whether the induction of Nrf2 has protective effects in response to diabetic assaults by using genetic induction of Nrf2 elicited by *Keap1* knockdown [40]. It has been shown that *Keap1*^*FA/–*^ mice display significant activation of Nrf2 signaling [40], and Nrf2 activation strongly suppresses the onset of diabetes mellitus in several models of diabetic mice, including the *db/db* model, nonobese diabetes model and high calorie diet-induced model [23,71]. Therefore, we prepared *Akita::Keap1*^*FA/–*^ and *Akita::Keap1*^*FA/+*^ mice. The blood glucose levels male *Akita::Keap1*^*FA/+*^ mice at 16-week-old were 741 ± 103 mg/dl (mean ± SD), while those of *Akita::Keap1*^*FA/–*^ mice were 370 ± 175 mg/dl, suggesting that the elevation of blood glucose levels can be ameliorated by the Nrf2 activation in *Akita::Keap1*^*FA/–*^ double mutant mice.

**Fig. 12:**
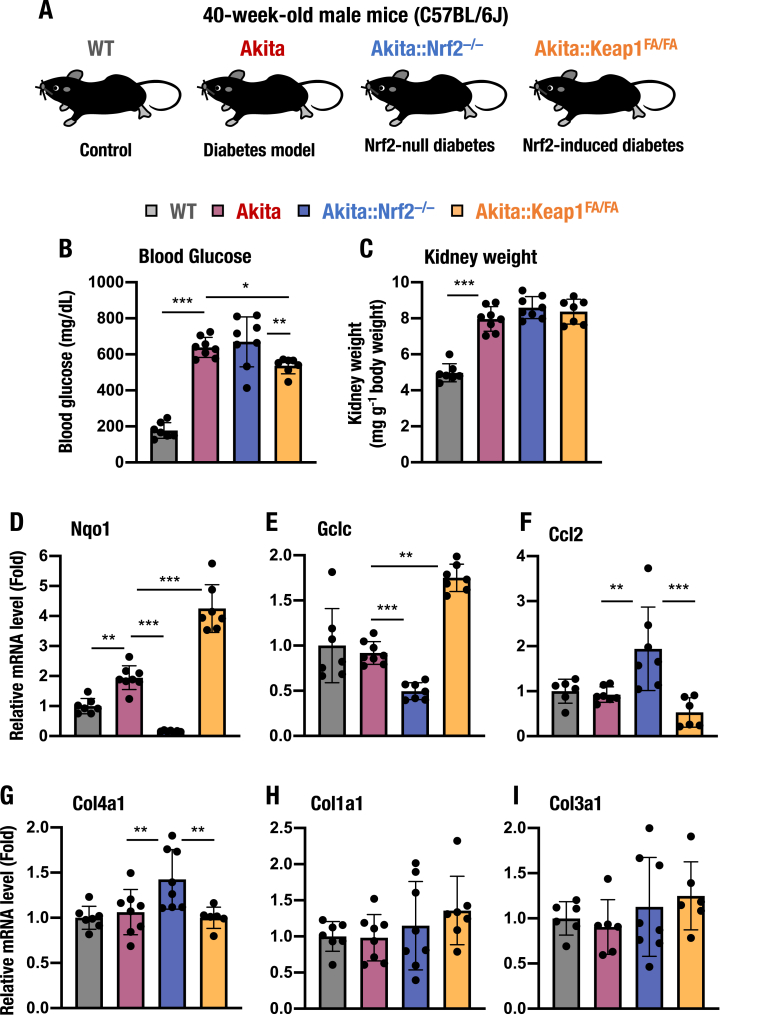
Forty-week-old *Akita::Keap1*^*FA/FA*^ and *Akita::Nrf2*^*−/−*^ mice. (A) Schematic presentation of 40-week-old WT, *Akita*, *Akita::Nrf2*^*−/−*^ and *Akita::Keap1*^*FA/FA*^ mice. (B and C) Blood glucose levels (B) and kidney weights normalized by body weight (C) for 40-week-old male WT, *Akita*, *Akita::Nrf2*^*−/−*^ and *Akita::Keap1*^*FA/FA*^ mice. (D–I) mRNA expression levels of the genes *Nqo1* (D), *Gclc* (E), *Ccl2* (MCP-1, F), *Col4a1* (G), *Col1a1* (H) and *Col3a1* (I) in the kidneys of 40-week-old male WT, *Akita*, *Akita::Nrf2*^*−/−*^ and *Akita::Keap1*^*FA/FA*^ mice. The expression level was normalized to *Hprt* expression and quantified as the fold increase relative to WT mice, which was set as 1. The results are presented as the mean ± SD. Statistical analyses were performed using ANOVA followed by Fisher's LSD post hoc test. *P < 0.05, **P < 0.01 and ***P < 0.001.

We next challenged the evaluation of the antidiabetic effects of Nrf2 by utilizing much milder and chronic activation conditions. To this end, we employed 40-week-old male mice and *Keap1*^*FA/FA*^ mice for this study, as the *Keap1*^*FA/FA*^ mice show much milder activation of Nrf2 than *Keap1*^*FA/–*^ mice [40]. We crossed *Keap1*^*FA/FA*^ mice on a C57BL/6J background with *Akita* mice to generate *Akita::Keap1*^*FA/FA*^ mice (Fig. 12A). We first examined blood glucose levels and found that, while the blood glucose level was severely elevated in the 40-week-old *Akita* mice, the level was significantly downregulated in the *Akita::Keap1*^*FA/FA*^ mice (Fig. 12B). The levels were comparable between *Akita* and *Akita::Nrf2*^*−/−*^ mice. These results support our observations that Nrf2 suppress the blood glucose level in diabetic model mice [10,[23], [24], [25],^66^,^72^]. The kidney weights were elevated in *Akita* mice, and the increases were comparable among *Akita*, *Akita::Nrf2*^*−/−*^ and *Akita::Keap1*^*FA/FA*^ mice (Fig. 12C).

Through the analyses of the *Akita::Keap1*^*FA/FA*^ mouse kidneys, we found that the expression level of *Nqo1* gene was significantly elevated in *Akita::Keap1*^*FA/FA*^ mouse kidneys compared to *Akita* mouse kidneys, while the expression level was strongly decreased in the kidneys of *Akita::Nrf2*^*−/−*^ mice (Fig. 12D). The expression level of *Gclc* gene was also significantly induced in the *Akita::Keap1*^*FA/FA*^ mouse kidneys, while the level was decreased in the *Akita::Nrf2*^*−/−*^ mouse kidneys (Fig. 12E). In contrast, the expression level of *Ccl2* gene was significantly increased in *Akita::Nrf2*^*−/−*^ mouse kidneys, while the level was comparable between WT, *Akita* and *Akita::Keap1*^*FA/FA*^ mouse kidneys (Fig. 12F). These results demonstrate that Nrf2 induces expressions of detoxifying enzyme genes, while suppresses proinflammatory cytokine and chemokine gene expressions in diabetic kidneys [20].

While we have observed that loss of Nrf2 induces the expression of fibrosis-related genes in diabetic *Akita* mouse kidneys (Fig. 6C–G, Fig. 11C), there is evidence that induction of Nrf2 increases fibrosis-related gene expression in three stress-inducible experimental kidney injury models, suggesting that Nrf2 induction exacerbates kidney damage [27]. To address this discrepancy, we examined the expression of fibrosis-related genes in the kidneys of 40-week-old diabetic mice. We found that *Col4a1* gene expression was higher in the kidneys of *Akita::Nrf2*^*−/−*^ mice than in those of *Akita* mice (Fig. 12G). In contrast, *Col4a1* expression was lower in *Akita::Keap1*^*FA/FA*^ mice than in *Akita::Nrf2*^*−/−*^ mice. We also evaluated *Col1a1* and *Col3a1* gene expression and found that it was comparable among these four genotype groups (Fig. 12H and I). These results demonstrate that Nrf2 does not contribute considerably to the progression of kidney fibrosis; rather, it contributes to the suppression of fibrosis-related gene expression in diabetic *Akita* mouse kidneys.

### 3.14 *Akita::Keap1*^*FA/FA*^ mice display improved tubular injury

**Fig. 13:**
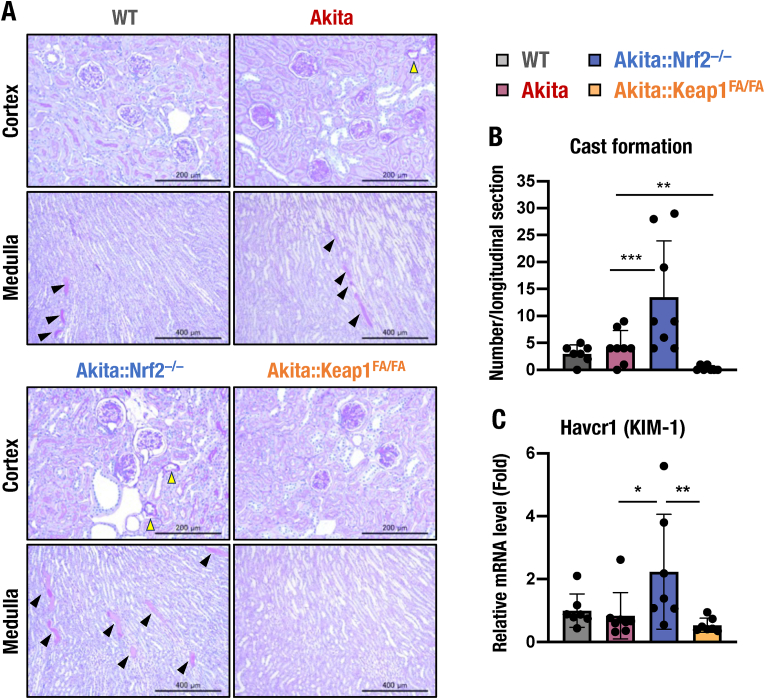
Tubulointerstitial changes in *Akita* mouse kidneys and Nrf2. (A) PAS-stained images of renal sections of 40-week-old male WT, *Akita*, *Akita::Nrf2*^*−/−*^ and *Akita::Keap1*^*FA/FA*^ mice. Yellow triangles indicate the abnormal tubules within the cortex (upper panel), and black triangles indicate the medullary cast (lower panel). *Bars*, 200 (upper) and 400 (lower) μm. (B) Numbers of quantified cast formations in each longitudinal section. (C) mRNA expression level of the *Havcr1* (KIM-1) gene in the kidneys of 40-week-old male WT, *Akita*, *Akita::Nrf2*^*−/−*^and *Akita::Keap1*^*FA/FA*^ mice. The expression level was normalized to *Hprt* expression and quantified as the fold increase relative to WT mice, which was set as 1. The results are presented as the mean ± SD. Statistical analyses were performed using ANOVA followed by Fisher's LSD post hoc test. *P < 0.05, **P < 0.01 and ***P < 0.001. (For interpretation of the references to color in this figure legend, the reader is referred to the Web version of this article.)

Histological examinations of the 40-week-old mouse kidneys revealed the presence of mesangial expansion, mesangiolysis and distal tubular dilation in the kidneys of *Akita::Nrf2*^*−/−*^ mice at 40 weeks of age (Fig. 13A, upper panels). The frequency of tubular dysmorphology increased following sustainable *Nrf2* deficiency, as shown by the increased number of injured tubules with thickened basement membranes together with the breakdown of tubular cells. However, tubular injury was rarely observed across the kidney sections of *Akita::Keap1*^*FA/FA*^ mice. Of note, there were obvious appearances of medullary hyaline casts in both *Akita* and *Akita::Nrf2*^*−/−*^ mouse kidneys (Fig. 13A, bottom panels; black arrowheads). The number of medullary hyaline casts was significantly increased in *Akita::Nrf2*^*−/−*^ mouse kidneys compared with *Akita* mouse kidneys. An important finding is that the medullary hyaline cast was eliminated in *Akita::Keap1*^*FA/FA*^ mouse kidneys (Fig. 13A and B).

As Nrf2 induction improved tubular interstitial changes in *Akita* mice, we also evaluated the mRNA expression level of the tubular injury marker *Havcr1*, which encodes KIM-1. We found that the expression of *Havcr1* was increased in the kidneys of *Akita::Nrf2*^*−/−*^ mice but decreased in the kidneys of *Akita::Keap1*^*FA/FA*^ mice (Fig. 13C). These results support our contention that Nrf2 contributes to the suppression of tubulointerstitial damage in *Akita* mouse kidneys under milder and chronic activation conditions.

## 4 Discussion

**Fig. 14:**
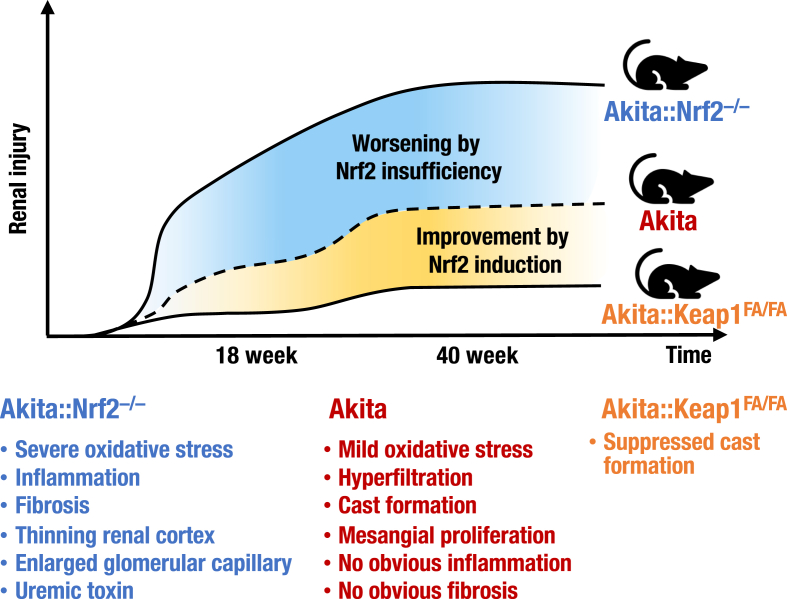
Relationship between Nrf2 levels and diabetic kidney disease symptoms in *Akita* diabetic model mice. *Akita* mice display mild oxidative stress, hyperfiltration, cast formation and modest mesangial proliferation but no obvious inflammation or fibrosis. *Akita::Nrf2*^*−/−*^ mice exhibit severe oxidative stress, inflammation, fibrosis, thinning renal cortex and modest mesangial proliferation. *Akita::Keap1*^*FA/FA*^ mice display suppressed cast formation.

To address whether Nrf2 influences the onset and/or progression of DKD, we exploited heterozygous *Akita* mice with a resistant background of C57BL/6J [73,74]. We evaluated the blood glucose levels of *Akita* mice with *Nrf2* knockout or *Keap1* knockdown alleles, along with histology, inflammation, oxidative stress, and fibrosis in the kidney. As summarized in Fig. 14, these analyses unequivocally indicate that the ablation of *Nrf2* from *Akita* mice exacerbates oxidative stress and inflammation in the kidneys, resulting in severe DKD with a thinning renal cortex, enlarged glomerular capillaries and fibrosis. In contrast, induction of Nrf2 activity in *Akita* mice by *Keap1* knockdown improves renal tubular injuries, including a decrease in cast formation. These results strongly support the conclusion that loss of Nrf2 activity accelerates, while gain of Nrf2 activity suppresses, the development of DKD. While *Nrf2* knockout in *Akita* mice has been reported to induce the expression of renin angiotensin system-related enzymes and decrease sodium-glucose cotransporters in the kidneys [68,75], to the best of our knowledge, this is the first study to show Nrf2 prevention of diabetic kidney damage in *Akita* mice.

Inflammation has long been suggested to be an important factor in the development of DKD [[76], [77], [78]], but clinical approaches targeting inflammation in DKD have not been well established. The inflammatory process in DKD seems to be driven by the accumulation of oxidative stressors, advanced glycation end products, and mechanical forces, *e.g.*, glomerular hyperfiltration and osmotic polyuria [53,62,79]. However, we did not find apparent inflammatory signs in the histological analyses of *Akita* mouse kidneys, which are on the C57BL/6J background resistant to the development of DKD [74,80]. In contrast, we found that *Nrf2*-deficient *Akita* mice display severe inflammation with increased infiltration of macrophages and increased chemokines and cytokines. These changes are fully normalized by the activation of Nrf2 signaling in *Akita::Keap1*^*FA/FA*^ mice, demonstrating that Nrf2 acts to suppress inflammation in DKD.

It has been reported that Nrf2 induction in *Keap1*^*FA/FA*^ mice exacerbates albuminuria in three models, *i.e.,* adriamycin, angiotensin II and protein overload models [27]. In the present study, we observed that the urine albumin/creatinine ratio was higher in 18-week-old *Akita* mice (54.9 ± 37.0 μg/mg creatinine, mean ± SD) than in WT mice (29.4 ± 10.1 μg/mg creatinine) and that the albumin/creatinine ratio of 18-week-old *Akita::Keap1*^*FA/FA*^ mice was 60.8 ± 10.3 μg/mg creatinine, which was comparable to that of *Akita* mice (*P* = 0.47, *Akita::Keap1*^*FA/FA*^ *vs*. *Akita* mice). In addition, while it has been demonstrated that Nrf2 induces *Col1a1* and *Col3a1* gene expression and reduces interstitial fibrosis in the kidneys of adriamycin model *Keap1*^*FA/FA*^ mice [27], we found in this study that the expression of *Col1a1* and *Col3a1* was not increased in 40-week-old diabetic *Akita::Keap1*^*FA/FA*^ mice. While we do not have plausible explanations for why albuminuria and fibrosis were aggravated in the adriamycin, angiotensin II and protein overload *Keap1*^*FA/FA*^ mice, the results of our analyses of *Akita::Keap1*^*FA/FA*^ mice support our contention that induction of Nrf2 ameliorates diabetic kidney injuries. Nrf2 induction has also been shown to protect against streptozotocin-induced diabetic kidney injuries [81] and ischemic kidney injuries [28].

Hyperglycemia provokes oxidative stress-induced tissue damage through ROS generation and antioxidant defense attenuation. GSH serves as an abundant and important antioxidant in this situation [82,83]. In *Akita* mouse kidneys, we detected a decrease in GSH levels, which deteriorated upon the depletion of Nrf2. This may be due to the downregulation of GSH synthetic or transferring enzymes, as the genes for these enzymes are intimately regulated by Nrf2 [48,49,66]. In fact, *Nrf2* deficiency predisposes *Akita* mice on a resistant background to oxidative stress-induced kidney injury, which is manifested by an increase in the oxidative stress marker 8-OHdG. In contrast, activation of Nrf2 signaling maintains the expression of GSH synthesis-related genes in *Akita* mouse kidneys. Indeed, dietary intake of *N*-acetylcysteine or the upregulation of the GSH level has been shown to improve DKD in experimental animal models [84,85], which has raised high interest in developing antioxidant adjuvant therapy for DKD. Consistently, Nrf2-inducing chemicals, such as bardoxolone methyl, appear to alleviate DKD injury, perhaps by raising the efficiency of the antioxidant system.

As glomerular changes, including mesangial expansion, characterize the early features of DKD [61], we examined how Nrf2 protects kidneys from such structural changes. We found that a dilation of capillary loops is the most prominent change in the glomeruli of *Akita::Nrf2*^*−/−*^ mice. Mesangiolysis is a vicious process recognized by attenuated mesangium and dilated capillary lumina in various glomerular diseases [60,65]. Our present results revealed that enhanced mesangiolysis occurs upon *Nrf2* ablation. As the 8-OHdG-positive cells are increased in the glomerular tuft of the *Akita::Nrf2*^*−/−*^ mice, the enhanced mesangiolysis may result from persistent oxidative stress-mediated damage in mesangial and/or endothelial cells. The xanthine-oxidase/xanthine mixture is reported to contribute to the production of massive radical superoxide anions, which promote mesangiolysis in the rabbit kidney, indicating that ROS contribute to the pathogenesis of mesangiolysis [86]. Oxidative stress-mediated mesangiolysis has also been reported in other mouse models, including endothelial nitric oxide synthase knockout mice and endothelial cell-specific autophagy-deficient mice [[87], [88], [89]]. In DKD patients, the presence of mesangiolysis serves as an independent risk factor for poor renal outcome [90]. Therefore, protection of the glomerulus against mesangiolysis is an important process for preventing DKD progression.

Another prominent histological finding in *Nrf2*-deficient *Akita* mice is distal tubular dilation in kidney sections. One plausible explanation of the dilation is the hyperglycemia-induced osmotic polyuria with increased tubular fluid pressure, which has been reported to trigger the change in DKD [62,91]. It appeared that polyuria was more pronounced in *Akita::Nrf2*^*−/−*^ mice, which displayed higher blood glucose levels, larger urine output, and lower urine osmolality than *Akita* mice. In addition, dilated distal tubules have been observed in the kidneys of manganese superoxide dismutase knockout mice, which contributes to scavenging mitochondria-derived superoxide radicals and defending against oxidative stress [63]. Nitrotyrosine-positive staining is localized in the regions that display deficiency of manganese superoxide dismutase activity, indicating overwhelmed oxidative stress in these tubules. We found in this study that an increased number of 8-OHdG-positive epithelial cells resides in the abnormal distal tubules of *Akita::Nrf2*^*−/−*^ mouse kidneys. In addition, an increase in the expression of *Ccl2* and *Il6* has also been observed, showing very good agreement with the observation that the expression of chemokine MCP-1 (*Ccl2*) is increased in the dilated distal tubules of diabetic mice [[92], [93], [94], [95]]. These results suggest that the dilated distal tubules contribute to the development of inflammation in *Nrf2* ablation.

Recently, it was reported that *Nrf2* depletion in *Akita* mice decreases SGLT2 expression in renal proximal tubular cells and increases fractional excretion of glucose, resulting in a decrease in the serum glucose level in *Akita::Nrf2*^*−/−*^ mice [68]. In contrast, we found in this study that the expression of *Slc5a2* (encoding SGLT2) was not altered significantly in the kidneys of *Akita::Nrf2*^*−/−*^ mice and that fractional excretion of glucose was lower in *Akita::Nrf2*^*−/−*^ mice than in *Akita* mice. We also found that blood glucose levels were elevated in *Akita::Nrf2*^*−/−*^ mice compared with *Akita* mice. We do not have clear explanations for these discrepancies, but we surmise that several points may be pertinent. We evaluated the expression of *Slc5a2* and levels of whole blood glucose using whole kidney samples and a glucometer (*i.e.,* the enzyme electrode method), respectively, and we exploited a *Nrf2*-knockout *Nfe2l2*^*tm1Mym*^ mouse line established by our laboratory [19]. In contrast, a previous study determined the expression of *Slc5a2* and the levels of serum glucose with renal proximal tubular cells and the colorimetric assay method, respectively. Additionally, the study utilized a distinct *Nrf2*-knockout *Nfe2l2*^*tm1Ywk*^ mouse line [68,75]. We surmise that these differences in experimental conditions may have resulted in the discrepancies in phenotypes. Whether the *Slc5a2* gene is under the influence of Nrf2 is an important question in kidney physiology; thus, this question should be addressed through multifaceted approaches.

In this study, we found that both CE(18:2) and CE(20:4) levels were high in mouse plasma but were decreased in the plasma of *Nrf2*^*−/−*^ and *Akita::Nrf2*^*−/−*^ mice. Both CE(18:2) and CE(20:4) are detected at high levels in LDL particles [96], and high serum LDL levels are known to accelerate albuminuria [97] and elevations in serum creatinine levels [98]. Therefore, the declines in plasma CE(18:2) and CE(20:4) levels may have resulted in the downregulation of plasma LDL levels and alleviation of kidney pathology in *Nrf2*^*−/−*^ and *Akita::Nrf2*^*−/−*^ mice. However, we observed that kidney function deteriorated in *Akita::Nrf2*^*−/−*^ mice. Interestingly, an increase in serum TG levels has been found to accelerate the decline in GFR [99] and to be a risk factor for diabetic nephropathy [100]. We found in our metabolome analysis that plasma TG levels were markedly elevated in *Akita::Nrf2*^*−/−*^ mice. Therefore, high TG levels may have overridden the declines in CE(18:2) and CE(20:4) levels and aggravated DKD in *Akita::Nrf2*^*−/−*^ mice.

## 5 Conclusion

This study demonstrates the protective effect of Nrf2 in the kidneys of *Akita* diabetic model mice, which is executed through the elaborate anti-inflammatory and antioxidative actions of Nrf2, enlightening the possibility of the clinical use of Nrf2 inducers for treating DKD.

## Authors’ contributions

Y.L. was responsible for conceptualization, investigation, methodology, and writing the original draft. A.U. was responsible for the project design, methodology, investigation, writing, review and editing. R.S., N.M., E.H. and D.S. were responsible for the investigation and validation. H.L. was responsible for project design and supervision. M.Y. was responsible for funding acquisition, supervision, project design and administration, and writing review and editing.

## Funding

This research was supported by the Platform Project for Supporting Drug Discovery and Life Science Research [Basis for Supporting Innovative Drug Discovery and Life Science Research (BINDS)] from the 10.13039/100009619Japan Agency for Medical Research and Development (10.13039/100009619AMED), Japan, grant number JP21am0101001 (MY); the Tohoku Medical Megabank Project from the 10.13039/501100001700Ministry of Education, Culture, Sports, Science and Technology (10.13039/501100001700MEXT) and the 10.13039/100009619AMED, Japan, grant number JP20km0105001 and JP20km0105002 (MY); Grants-in-Aid for Scientific Research from the 10.13039/501100001691Japan Society for the Promotion of Science (10.13039/501100001691JSPS), Japan, grant numbers 19H05649 (MY), 20K07352 (AU); the Takeda Foundation, Japan (MY) and the 10.13039/100007428Naito Foundation, Japan (MY); and 10.13039/501100004543China Scholarship Council, China, grant number 201806370095 (YL).

## Ethics approval and consent to participate

All experimental procedures conformed to the “Regulations for Animal Experiments and Related Activities at Tohoku University” and were reviewed by the Institutional Laboratory Animal Care and Use Committee of Tohoku University.

## Declaration of competing interest

The authors declare that they have no known competing financial interests or personal relationships that could have appeared to influence the work reported in this paper.

## Data Availability

No data was used for the research described in the article.
