# Supplementary material for: Nrf2 deficiency deteriorates diabetic kidney disease in Akita model mice
Source: Redox Biol. 2022 Oct 28;58:102525. doi: 10.1016/j.redox.2022.102525 (PMC9641024; doi:10.1016/j.redox.2022.102525)
Supplement: Multimedia component 1 [file mmc1.pdf]

**Supplementary Information**

# *Nrf2* deficiency deteriorates diabetic kidney disease in *Akita* model mice

**Yexin Liu, Akira Uruno, Ritsumi Saito, Naomi Matsukawa,**

**Eiji Hishinuma, Daisuke Saigusa, Hong Liu, Masayuki Yamamoto**

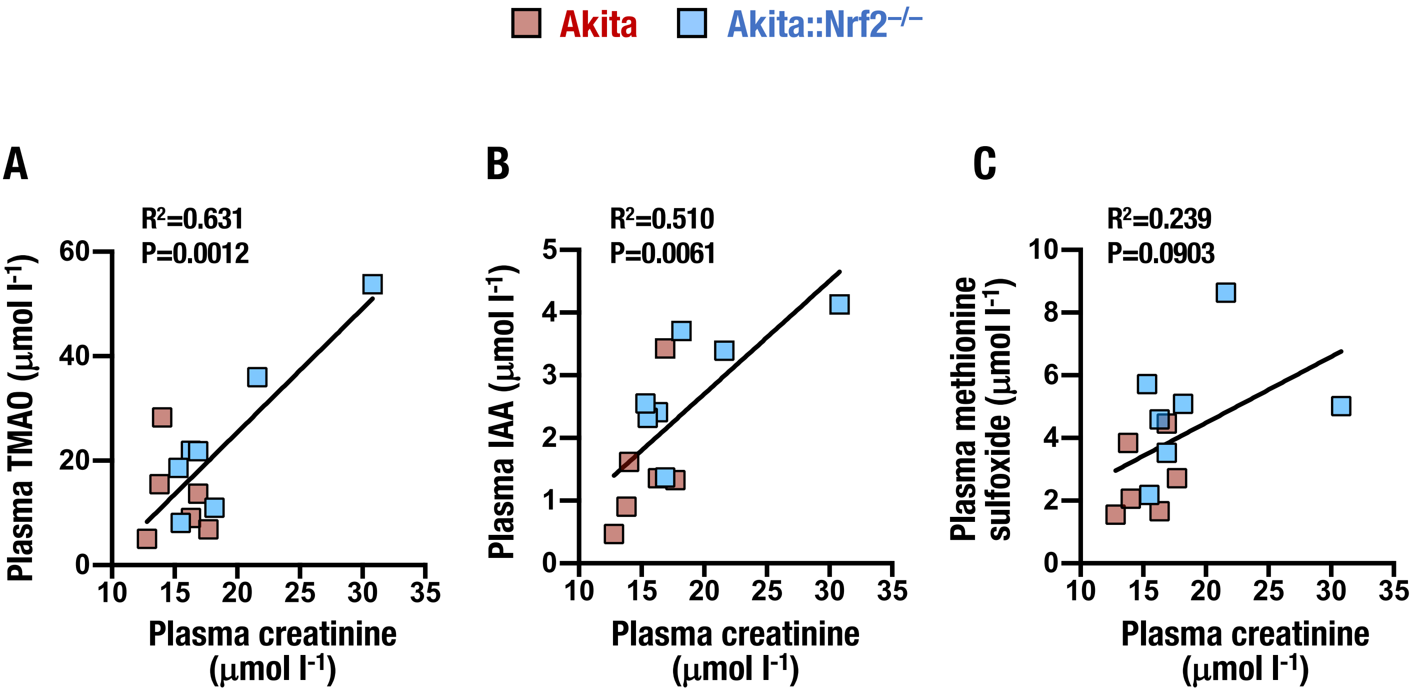


**Supplementary Figure 1.** Relationships between the plasma creatinine level and uremic toxins. The plots show the levels of plasma creatinine levels (A-C), the uremic toxins TMAO (A) and IAA (B) and the oxidative stress-related metabolite methionine sulfoxide (C) in *Akita* and *Akita::Nrf2*$^{-/-}$ mice. *P* values and Pearson's correlation coefficient $R^2$ are displayed.

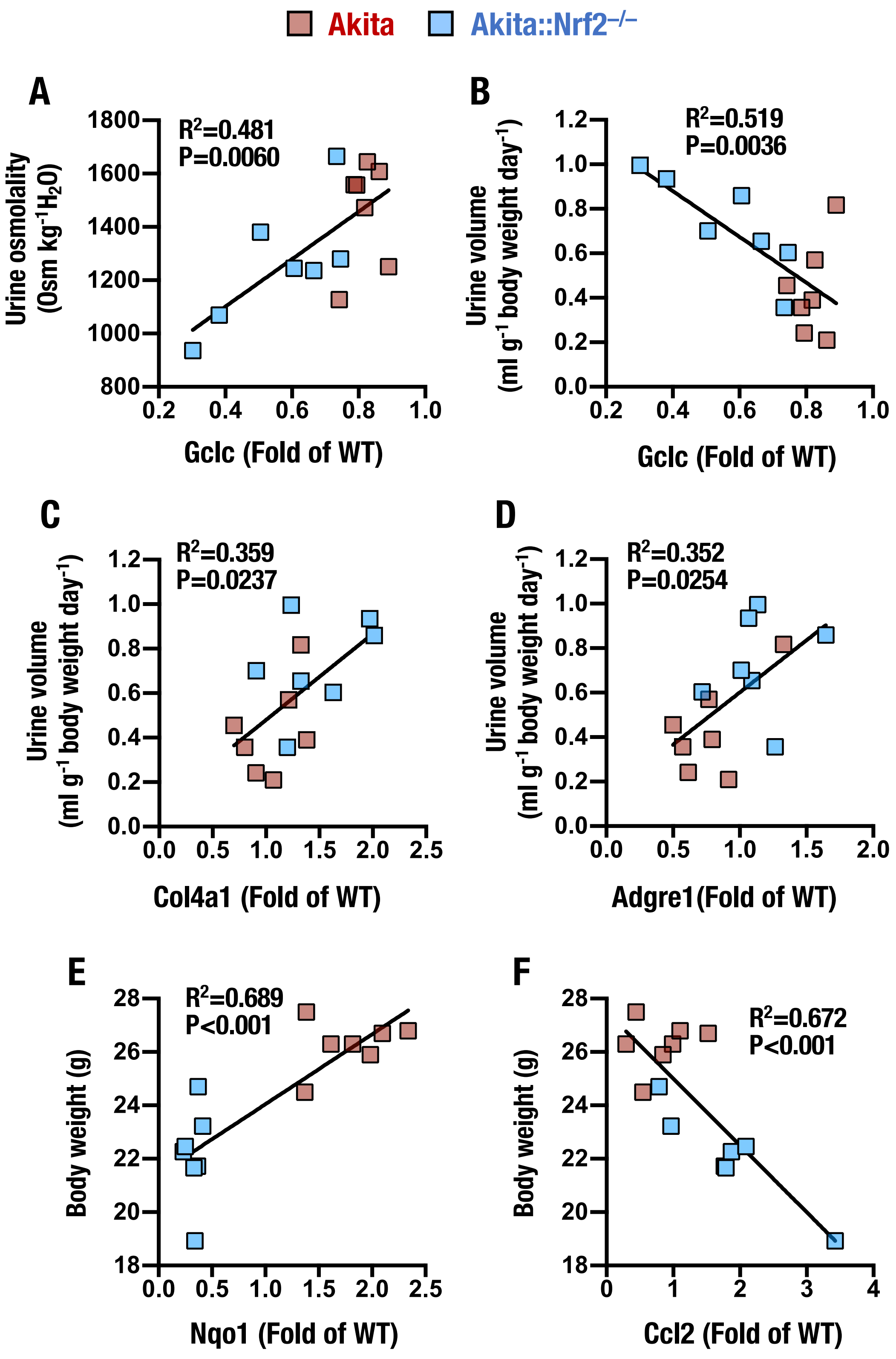


**Supplementary Figure 2.** Relationship between gene expression in kidneys and DKD-related phenotypes. (A-F) Plots of the expression of genes such as *Gclc* (A and B), *Col4a1* (C), *Adgre1* (F4/80, D), *Nqo1* (E), and *Ccl2* (MCP-1, F) in the kidneys and DKD-related phenotypic parameters such as urine osmolarity (A), urine volume normalized to body weight (B-D) and body weight (E and F) in *Akita* and *Akita::Nrf2*$^{-/-}$ mice. The *P* values and Pearson's correlation coefficient $R^2$ are displayed.

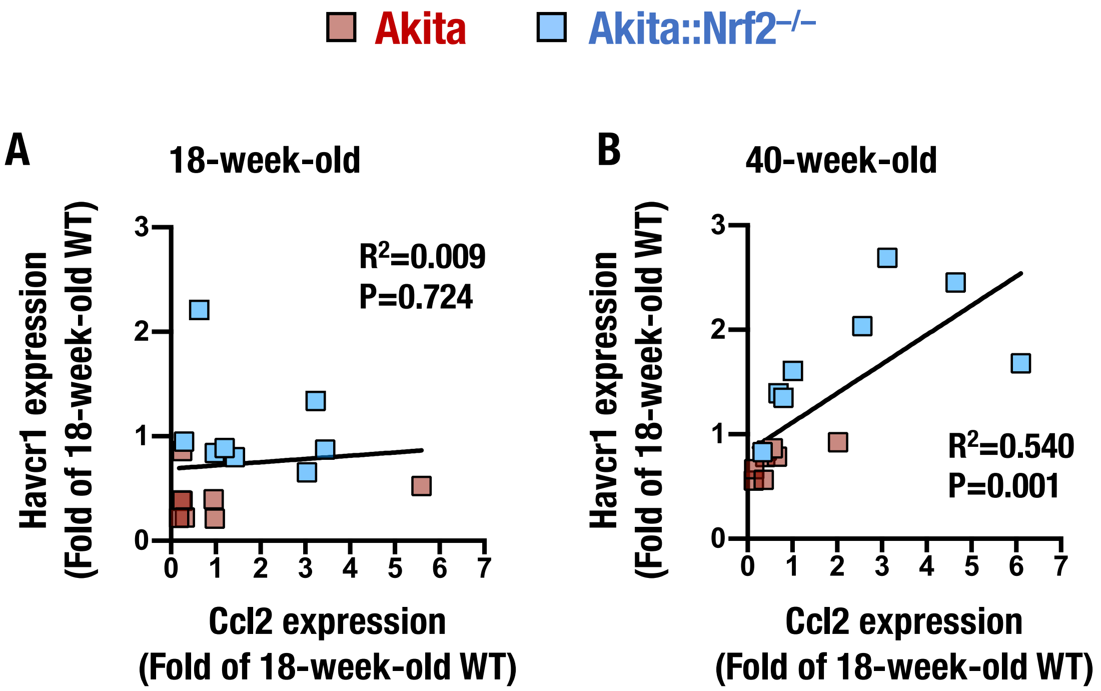


**Supplementary Figure 3.** Comparison between 18- and 40-week-old *Akita* mice of susceptibility to *Nrf2* depletion. The plots show the expression of the *Hrvcr1* gene and the *Ccl2* gene in the kidneys of 18-week-old (A) and 40-week-old (B) *Akita* and *Akita::Nrf2*$^{-/-}$ mice. The *P* values and Pearson's correlation coefficient $R^2$ are displayed.

**Table S1.** Primers for qRT-PCR.

| Gene Symbol | Forward/Reverse/ Probe | Sequence |
|---|---|---|
| *Hprt* | Forward | 5'-CTGGTGAAAAGGACCTCTCG-3' |
| | Reverse | 5'-TGAAGTACTCATTATAGTCAAGGG-3' |
| *Nqo1* | Forward | 5'-AGCTGGAAGCTGCAGACCTG-3' |
| | Reverse | 5'-CCTTTCAGAATGGCTGGCA-3' |
| | Probe | 5'-ATTTCAGTTCCCATTGCAGTGGTTTGGG-3' |
| *Hmox1* | Forward | 5'-GTGATGGAGCGTCCACAGC-3' |
| | Reverse | 5'-TTGGTGGCCTCCTTCAAGG-3' |
| | Probe | 5'-CGACAGCATGCCCCAGGATTTGTC-3' |
| *Gclc* | Forward | 5'-ATCTGCAAAGGCGGCAAC-3' |
| | Reverse | 5'-ACTCCTCTGCAGCTGGCTC-3' |
| | Probe | 5'-ACGGGTGCAGCAAGGCCCA-3' |
| *Gclm* | Forward | 5'-TGACTCACAATGACCCGAAA-3' |
| | Reverse | 5'-GATGCTTTCTTGAAGAGCTTCCT-3' |
| | Probe | 5'-ACTGCTCTCTGAGGCAAGTTTC-3' |
| *Gsr* | Forward | 5'-GGCGTGGAGGTGTTGAAG-3' |
| | Reverse | 5'-GCACGGAAGTCACCACTTG-3' |
| *Havcr1* (KIM-1) | Forward | 5'-ACATATCGTGGAATCACAACGAC-3' |
| | Reverse | 5'-ACTGCTCTTCTGATAGGTGACA-3' |
| *Lcn2* (NGAL) | Forward | 5'-GCAGGTGGTACGTTGTGGG-3' |
| | Reverse | 5'-CTCTTGTAGCTCATAGATGGTGC-3' |
| *Ccl2* (MCP-1) | Forward | 5'-GGCTCAGCCAGATGCAGTTAA-3' |
| | Reverse | 5'-CCTACTCATTGGGATCATCTTGCT-3' |
| *Il1b* | Forward | 5'-TGTAATGAAAGACGGCACACC-3' |
| | Reverse | 5'-TCTTCTTTGGGTATTGCTTGG-3' |
| *Il6* | Forward | 5'-GCTACCAAACTGGATATAATCAGGA-3' |
| | Reverse | 5'-CCAGGTAGCTATGGTACTCCAGAA-3' |
| *Adgre1* (F4/80) | Forward | 5'-GCCTGGACGAATCCTGTGAA-3' |
| | Reverse | 5'-TCCCAGAGTGTTGATGCAAA-3' |
| *Tgfb1* (TGF-β1) | Forward | 5'-TGGAGCAACATGTGGAACTC-3' |
| | Reverse | 5'-CAGCAGCCGGTTACCAAG-3' |
| *Fn1* | Forward | 5'-GATGCACCGATTGTCAACAG-3' |
| | Reverse | 5'-ACTCTGATCAGCATGGACCA-3' |
| *Col1a1* | Forward | 5'-ATGTTCAGCTTTGTGGACCTC-3' |
| | Reverse | 5'-TCCCTCGACTCCTACATCTTC-3' |
| *Col3a1* | Forward | 5'-TCCCCTGGAATCTGTGAATC-3' |
| | Reverse | 5'-TGAGTCGAATTGGGGAGAAT-3' |
| *Col4a1* | Forward | 5'-GTCTGGCTTCTGCTGCTCTT-3' |
| | Reverse | 5'-CACATTTTCCACAGCCAGAG-3' |

**Table S2.** Correlation between gene expression in kidneys and DKD-related phenotypic parameters of Nrf2–/– and Akita::Nrf2–/– mice.

| Gene expression | vs. Urine osmotality | | | | vs. Urine volume | | | | vs. Kidney weight | | | | vs. Body Weight | | | |
|---|---|---|---|---|---|---|---|---|---|---|---|---|---|---|---|---|
| | Correlation | 95% CI | | *P* value | Correlation | 95% CI | | *P* value | Correlation | 95% CI | | *P* value | Correlation | 95% CI | | *P* value |
| | | Upper | Lower | | | Upper | Lower | | | Upper | Lower | | | Upper | Lower | |
| *Nqo1* | 0.5043 | -0.0359 | 0.8164 | 0.0659 | -0.5677 | -0.8441 | -0.0532 | 0.0342* | 0.1354 | -0.4257 | 0.6214 | 0.6443 | 0.8301 | 0.5354 | 0.9446 | 0.0002*** |
| *Hmox1* | 0.4014 | -0.1641 | 0.7683 | 0.1549 | -0.5102 | -0.8191 | 0.0279 | 0.0623 | -0.1033 | -0.6010 | 0.452 | 0.7252 | 0.5206 | -0.0138 | 0.8237 | 0.0563 |
| *Gclc* | 0.6934 | 0.2576 | 0.8948 | 0.0060** | -0.7206 | -0.9051 | -0.3077 | 0.0036** | -0.3517 | -0.7435 | 0.22 | 0.2176 | 0.4943 | -0.0492 | 0.8119 | 0.0724 |
| *Gclm* | -0.0430 | -0.5608 | 0.499 | 0.8841 | 0.338 | -0.2347 | 0.7365 | 0.2372 | 0.0787 | -0.4716 | 0.5848 | 0.7892 | 0.1007 | -0.4542 | 0.5992 | 0.732 |
| *Gsr* | -0.0673 | -0.5773 | 0.4804 | 0.8191 | -0.0695 | -0.5788 | 0.4787 | 0.8132 | 0.3336 | -0.2394 | 0.7342 | 0.2438 | 0.57 | 0.0566 | 0.845 | 0.0333* |
| *Il6* | -0.0544 | -0.5686 | 0.4903 | 0.8534 | 0.2751 | -0.2992 | 0.703 | 0.3412 | -0.5121 | -0.8199 | 0.0254 | 0.0612 | -0.5163 | -0.8218 | 0.0197 | 0.0587 |
| *Il1b* | 0.0293 | -0.5092 | 0.5513 | 0.9208 | 0.2065 | -0.3640 | 0.6643 | 0.4788 | -0.2780 | -0.7046 | 0.2963 | 0.336 | -0.7067 | -0.8998 | -0.2817 | 0.0047* |
| *Ccl2* (MCP-1) | -0.1359 | -0.6217 | 0.4253 | 0.6431 | 0.2997 | -0.2746 | 0.7163 | 0.2979 | -0.3745 | -0.7550 | 0.1948 | 0.1871 | -0.8194 | -0.9409 | -0.5110 | 0.0003*** |
| *Adgre1* (F4/80) | -0.2546 | -0.6917 | 0.3191 | 0.3797 | 0.593 | 0.0911 | 0.8547 | 0.0254* | -0.0854 | -0.5893 | 0.4663 | 0.7715 | -0.4521 | -0.7926 | 0.1033 | 0.1046 |
| *Fn1* | 0.2635 | -0.3105 | 0.6967 | 0.3626 | 0.037 | -0.5035 | 0.5566 | 0.9001 | -0.1099 | -0.6052 | 0.4467 | 0.7084 | -0.1273 | -0.6162 | 0.4325 | 0.6646 |
| *Col1a1* | -0.0140 | -0.5406 | 0.5204 | 0.9621 | 0.3514 | -0.2202 | 0.7434 | 0.2179 | -0.3509 | -0.7431 | 0.2208 | 0.2187 | -0.3570 | -0.7462 | 0.2141 | 0.2102 |
| *Col3a1* | 0.025 | -0.5124 | 0.5483 | 0.9324 | 0.3005 | -0.2737 | 0.7168 | 0.2965 | -0.3051 | -0.7193 | 0.269 | 0.2888 | -0.3378 | -0.7364 | 0.2348 | 0.2375 |
| *Col4a1* | -0.3625 | -0.7490 | 0.2081 | 0.2028 | 0.5987 | 0.0999 | 0.857 | 0.0237* | -0.1936 | -0.6567 | 0.3755 | 0.5072 | -0.3689 | -0.7523 | 0.201 | 0.1943 |
| *Tgfb1* | -0.1264 | -0.6157 | 0.4332 | 0.6668 | 0.431 | -0.1291 | 0.7826 | 0.1239 | 0.3801 | -0.1885 | 0.7578 | 0.1801 | -0.4873 | -0.8088 | 0.0584 | 0.0772 |
| *Havcr1* (KIM-1) | -0.1158 | -0.6090 | 0.4419 | 0.6934 | 0.172 | -0.3946 | 0.6439 | 0.5564 | -0.1788 | -0.6479 | 0.3886 | 0.5407 | -0.3015 | -0.7173 | 0.2727 | 0.2948 |
| *Lcn2* (NGAL) | 0.1821 | -0.3857 | 0.6499 | 0.5332 | -0.0750 | -0.5824 | 0.4744 | 0.7988 | -0.3442 | -0.7397 | 0.228 | 0.2281 | -0.1957 | -0.6579 | 0.3737 | 0.5026 |

*$P<0.05$, **$P<0.01$ and ***$P<0.001$.

**Table S3.** Correlation between uremic toxin/oxidation-related metabolites and other metabolites such as amino acids and lipids in the plasma of Nrf2–/– and Akita::Nrf2–/– mice.

| Metabolite | *vs.* Creatinine | | | | *vs.* TMAO | | | | *vs.* IAA | | | | *vs.* Methionine sulfoxide | | | |
|---|---|---|---|---|---|---|---|---|---|---|---|---|---|---|---|---|
| | Correlation | 95% CI | | *P* value | Correlation | 95% CI | | *P* value | Correlation | 95% CI | | *P* value | Correlation | 95% CI | | *P* value |
| | | Upper | Lower | | | Upper | Lower | | | Upper | Lower | | | Upper | Lower | |
| Ala | 0.5645 | 0.0196 | 0.8508 | 0.0444* | 0.449 | -0.1355 | 0.8017 | 0.1238 | 0.8259 | 0.5045 | 0.9463 | 0.0005*** | 0.8434 | 0.5462 | 0.952 | 0.0003** |
| Arg | 0.2361 | -0.3620 | 0.6965 | 0.4375 | 0.2539 | -0.3454 | 0.7061 | 0.4026 | 0.4923 | -0.0806 | 0.8207 | 0.0875 | 0.625 | 0.1128 | 0.8747 | 0.0224* |
| Asn | 0.4278 | -0.1612 | 0.7921 | 0.1448 | 0.1696 | -0.4207 | 0.659 | 0.5797 | 0.8033 | 0.4526 | 0.9388 | 0.0009*** | 0.6328 | 0.1257 | 0.8778 | 0.0203* |
| Asp | 0.2539 | -0.3454 | 0.7061 | 0.4026 | 0.163 | -0.4262 | 0.6552 | 0.5946 | 0.3759 | -0.2208 | 0.7678 | 0.2056 | 0.2221 | -0.3747 | 0.6888 | 0.4658 |
| Cys | 0.0535 | -0.5126 | 0.5871 | 0.8623 | 0.2823 | -0.3182 | 0.7211 | 0.3501 | -0.0854 | -0.6078 | 0.4886 | 0.7815 | 0.3904 | -0.2046 | 0.7747 | 0.1872 |
| Gln | -0.0363 | -0.5758 | 0.5252 | 0.9062 | -0.1187 | -0.6286 | 0.4625 | 0.6993 | 0.3775 | -0.2190 | 0.7686 | 0.2035 | -0.0017 | -0.5522 | 0.5498 | 0.9956 |
| Glu | 0.0611 | -0.5070 | 0.5921 | 0.8429 | -0.2737 | -0.7166 | 0.3265 | 0.3655 | 0.3763 | -0.2204 | 0.768 | 0.205 | 0.0204 | -0.5366 | 0.565 | 0.9472 |
| Gly | 0.3506 | -0.2483 | 0.7556 | 0.2402 | 0.0749 | -0.4966 | 0.6011 | 0.8078 | 0.7551 | 0.3496 | 0.9224 | 0.0028** | 0.6157 | 0.098 | 0.8712 | 0.0251* |
| His | 0.1616 | -0.4275 | 0.6543 | 0.598 | 0.0191 | -0.5376 | 0.5641 | 0.9507 | 0.6179 | 0.1015 | 0.872 | 0.0244* | 0.4896 | -0.0841 | 0.8195 | 0.0895 |
| Ile | 0.7744 | 0.3898 | 0.929 | 0.0019** | 0.7814 | 0.4047 | 0.9314 | 0.0016** | 0.5351 | -0.0225 | 0.8388 | 0.0595 | 0.4709 | -0.1081 | 0.8114 | 0.1043 |
| Leu | 0.7442 | 0.3275 | 0.9185 | 0.0035** | 0.7477 | 0.3345 | 0.9198 | 0.0033** | 0.4666 | -0.1136 | 0.8095 | 0.108 | 0.3932 | -0.2014 | 0.7761 | 0.1838 |
| Lys | 0.5753 | 0.0356 | 0.8552 | 0.0397* | 0.4831 | -0.0925 | 0.8167 | 0.0944 | 0.7187 | 0.2777 | 0.9095 | 0.0056** | 0.539 | -0.0171 | 0.8404 | 0.0574 |
| Met | 0.5101 | -0.0569 | 0.8283 | 0.0749 | 0.3688 | -0.2287 | 0.7644 | 0.215 | 0.8418 | 0.5425 | 0.9515 | 0.0003** | 0.8614 | 0.5909 | 0.9578 | 0.0002** |
| Phe | 0.654 | 0.1611 | 0.8858 | 0.0153* | 0.476 | -0.1017 | 0.8136 | 0.1002 | 0.7485 | 0.3361 | 0.92 | 0.0032** | 0.4993 | -0.0713 | 0.8237 | 0.0823 |
| Pro | 0.6078 | 0.0854 | 0.8681 | 0.0276* | 0.4762 | -0.1014 | 0.8137 | 0.1 | 0.865 | 0.6 | 0.959 | 0.0001*** | 0.8978 | 0.6864 | 0.9693 | <0.0001*** |
| Ser | 0.403 | -0.1902 | 0.7807 | 0.1721 | 0.2371 | -0.3611 | 0.697 | 0.4355 | 0.8164 | 0.4823 | 0.9431 | 0.0007*** | 0.7612 | 0.3621 | 0.9245 | 0.0025** |
| Thr | 0.5432 | -0.0112 | 0.8421 | 0.0551 | 0.5519 | 0.0013 | 0.8457 | 0.0505 | 0.7083 | 0.2581 | 0.9058 | 0.0067** | 0.9261 | 0.766 | 0.978 | <0.0001*** |
| Trp | -0.1272 | -0.6338 | 0.4557 | 0.6788 | -0.1832 | -0.6669 | 0.4091 | 0.5492 | -0.1106 | -0.6236 | 0.4689 | 0.719 | 0.0805 | -0.4923 | 0.6047 | 0.7937 |
| Tyr | 0.7422 | 0.3235 | 0.9178 | 0.0037** | 0.5008 | -0.0693 | 0.8243 | 0.0813 | 0.8658 | 0.602 | 0.9592 | 0.0001*** | 0.6539 | 0.1609 | 0.8858 | 0.0153* |
| Val | 0.7335 | 0.3063 | 0.9148 | 0.0043** | 0.7812 | 0.4042 | 0.9313 | 0.0016** | 0.59 | 0.0578 | 0.8611 | 0.0338* | 0.5907 | 0.0589 | 0.8614 | 0.0335* |
| Total TG | 0.1666 | -0.4232 | 0.6573 | 0.5864 | 0.5081 | -0.0596 | 0.8274 | 0.0763 | 0.1576 | -0.4308 | 0.652 | 0.6072 | 0.6112 | 0.0908 | 0.8694 | 0.0265* |
| Total SM | -0.1107 | -0.6236 | 0.4689 | 0.7189 | 0.2028 | -0.3920 | 0.678 | 0.5064 | -0.4825 | -0.8164 | 0.0933 | 0.095 | -0.3740 | -0.7669 | 0.2229 | 0.208 |
| Total CE | 0.0259 | -0.5327 | 0.5688 | 0.933 | 0.2163 | -0.3800 | 0.6856 | 0.4779 | -0.5094 | -0.8280 | 0.0578 | 0.0754 | -0.3745 | -0.7672 | 0.2224 | 0.2075 |
| Total LysoPC | -0.2018 | -0.6774 | 0.3929 | 0.5086 | 0.1161 | -0.4646 | 0.6269 | 0.7058 | -0.4134 | -0.7855 | 0.1782 | 0.1603 | -0.3095 | -0.7351 | 0.2911 | 0.3034 |

**P* <0.05, ***P* <0.01 and ****P* <0.001.

**Table S4.** Statistical parameters of 261 qualified metabolites (M261).

| M261 ID | Metabolite | Plasma concentration (µmol l$^{-1}$, mean±SD) | | | | Increased or decreased (*P*<0.05; *ns*, not significant.) | | | | | Fold change | | | | | Group |
|---|---|---|---|---|---|---|---|---|---|---|---|---|---|---|---|---|
| | | WT | *Nrf2*$^{-/-}$ | *Akita* | *Akita::Nrf2*$^{-/-}$ | *Nrf2*$^{-/-}$ vs. WT | *Akita* vs. WT | *Akita::Nrf2*$^{-/-}$ vs. WT | *Akita::Nrf2*$^{-/-}$ vs. *Nrf2*$^{-/-}$ | *Akita::Nrf2*$^{-/-}$ vs. *Akita* | *Nrf2*$^{-/-}$ vs. WT | *Akita* vs. WT | *Akita::Nrf2*$^{-/-}$ vs. WT | *Akita::Nrf2*$^{-/-}$ vs. *Nrf2*$^{-/-}$ | *Akita::Nrf2*$^{-/-}$ vs. *Akita* | |
| 1 | Trigonelline | 1.77 ± 0.50 | 1.81 ± 0.76 | 3.73 ± 1.36 | 4.91 ± 2.48 | ns | ns | Increased | Increased | ns | 1.022 | 2.106 | 2.769 | 2.710 | 1.315 | |
| 2 | TMAO | 5.03 ± 1.54 | 5.16 ± 2.05 | 13.08 ± 7.72 | 24.45 ± 14.6 | ns | ns | Increased | Increased | Increased | 1.027 | 2.603 | 4.867 | 4.737 | 1.870 | c |
| 3 | Ala | 661.5 ± 124.42 | 633 ± 181.75 | 557.83 ± 200.81 | 926 ± 261.19 | ns | ns | ns | ns | ns | 0.957 | 0.843 | 1.400 | 1.463 | 1.660 | |
| 4 | Arg | 101.62 ± 38.39 | 120.56 ± 34.65 | 94.15 ± 25.49 | 121.93 ± 43.33 | ns | ns | ns | ns | ns | 1.186 | 0.927 | 1.200 | 1.011 | 1.295 | |
| 5 | Asn | 49.33 ± 11.52 | 62.06 ± 20.66 | 37.25 ± 14.54 | 71.26 ± 27.73 | ns | ns | ns | ns | Increased | 1.258 | 0.755 | 1.444 | 1.148 | 1.913 | c |
| 6 | Asp | 12.44 ± 3.32 | 13.58 ± 3.5 | 11.16 ± 2.4 | 14.07 ± 3.8 | ns | ns | ns | ns | ns | 1.092 | 0.898 | 1.131 | 1.036 | 1.261 | |
| 7 | Cys | 65.18 ± 5.08 | 79.87 ± 25 | 61.8 ± 2.28 | 62.24 ± 10.38 | ns | ns | ns | ns | ns | 1.225 | 0.948 | 0.955 | 0.779 | 1.007 | |
| 8 | Gln | 746.5 ± 82.18 | 799.43 ± 81.94 | 585.17 ± 72.63 | 665.29 ± 151.46 | ns | Decreased | ns | ns | ns | 1.071 | 0.784 | 0.891 | 0.832 | 1.137 | |
| 9 | Glu | 52 ± 12.69 | 60.83 ± 12 | 44.77 ± 6.98 | 53.39 ± 19.51 | ns | ns | ns | ns | ns | 1.170 | 0.861 | 1.027 | 0.878 | 1.193 | |
| 10 | Gly | 307.17 ± 53.94 | 343.71 ± 64.97 | 193 ± 50.55 | 287.71 ± 65.09 | ns | Decreased | ns | ns | Increased | 1.119 | 0.628 | 0.937 | 0.837 | 1.491 | c |
| 11 | His | 68.8 ± 6.05 | 71.9 ± 18.58 | 61.35 ± 6.34 | 77.83 ± 22.42 | ns | ns | ns | ns | ns | 1.045 | 0.892 | 1.131 | 1.082 | 1.269 | |
| 12 | Ile | 98.3 ± 12.17 | 104.64 ± 23.33 | 185.5 ± 42.62 | 206.71 ± 76.61 | ns | Increased | Increased | Increased | ns | 1.065 | 1.887 | 2.103 | 1.975 | 1.114 | |
| 13 | Leu | 117.6 ± 21 | 104.74 ± 25.39 | 268.83 ± 88.81 | 295 ± 143.91 | ns | Increased | Increased | Increased | ns | 0.891 | 2.286 | 2.509 | 2.816 | 1.097 | |
| 14 | Lys | 253.5 ± 24.58 | 272.57 ± 50.19 | 181.67 ± 22.65 | 212.43 ± 41.73 | ns | Decreased | ns | Decreased | ns | 1.075 | 0.717 | 0.838 | 0.779 | 1.169 | |
| 15 | Met | 52.58 ± 9.94 | 50.81 ± 12.43 | 35.28 ± 15.85 | 56.19 ± 17.09 | ns | ns | ns | ns | ns | 0.966 | 0.671 | 1.069 | 1.106 | 1.592 | |
| 16 | Phe | 80.97 ± 9.81 | 84.91 ± 25.94 | 90.72 ± 13.77 | 116.66 ± 38.84 | ns | ns | ns | ns | ns | 1.049 | 1.120 | 1.441 | 1.374 | 1.286 | |
| 17 | Pro | 110.53 ± 24.45 | 114.19 ± 39.38 | 98.55 ± 59.17 | 184.29 ± 61.82 | ns | ns | ns | ns | ns | 1.033 | 0.892 | 1.667 | 1.614 | 1.870 | |
| 18 | Ser | 135.17 ± 22.81 | 138.14 ± 36.99 | 111.78 ± 38.16 | 164.86 ± 46.06 | ns | ns | ns | ns | ns | 1.022 | 0.827 | 1.220 | 1.193 | 1.475 | |
| 19 | Thr | 145.33 ± 29.58 | 117.57 ± 36.25 | 132 ± 43.73 | 172.33 ± 56.9 | ns | ns | ns | ns | ns | 0.809 | 0.908 | 1.186 | 1.466 | 1.306 | |
| 20 | Trp | 76.32 ± 12.15 | 74.7 ± 8.34 | 62.7 ± 6.43 | 59.21 ± 9.04 | ns | Decreased | Decreased | Decreased | ns | 0.979 | 0.822 | 0.776 | 0.793 | 0.944 | |
| 21 | Tyr | 64.52 ± 8.23 | 79 ± 21.01 | 71.78 ± 26.21 | 105.80 ± 45.79 | ns | ns | ns | ns | ns | 1.224 | 1.113 | 1.640 | 1.339 | 1.474 | |
| 22 | Val | 229.00 ± 22.19 | 223.43 ± 57.49 | 395.33 ± 89.91 | 459.29 ± 128.14 | ns | Increased | Increased | Increased | ns | 0.976 | 1.726 | 2.006 | 2.056 | 1.162 | |
| 23 | 1-Met-His | 11.08 ± 1.35 | 9.79 ± 2.23 | 8.18 ± 1.13 | 8.09 ± 2.26 | ns | Decreased | Decreased | ns | ns | 0.884 | 0.738 | 0.730 | 0.827 | 0.990 | |
| 24 | 3-Met-His | 2.72 ± 0.33 | 3.67 ± 0.71 | 3.59 ± 0.72 | 4.21 ± 1.23 | ns | ns | Increased | ns | ns | 1.352 | 1.321 | 1.550 | 1.147 | 1.173 | |
| 25 | 5-AVA | 0.35 ± 0.25 | 0.91 ± 1.86 | 0.33 ± 0.25 | 0.28 ± 0.26 | ns | ns | ns | ns | ns | 2.607 | 0.954 | 0.801 | 0.307 | 0.840 | |
| 26 | AABA | 3.67 ± 0.59 | 3.28 ± 0.8 | 3.84 ± 0.76 | 5.04 ± 1.69 | ns | ns | ns | ns | ns | 0.894 | 1.046 | 1.371 | 1.533 | 1.310 | |
| 27 | ADMA | 0.72 ± 0.11 | 0.94 ± 0.12 | 0.76 ± 0.18 | 0.85 ± 0.12 | ns | Increased | ns | ns | ns | 1.299 | 1.047 | 1.173 | 0.903 | 1.121 | |
| 28 | α-Aminoadipic acid | 5.16 ± 1.01 | 9.35 ± 2.81 | 2.59 ± 0.56 | 7.2 ± 3.45 | Increased | ns | ns | ns | Increased | 1.811 | 0.501 | 1.396 | 0.771 | 2.783 | b |
| 29 | Anserine | 1.2 ± 0.45 | 1.58 ± 0.86 | 0.81 ± 0.4 | 1.18 ± 0.45 | ns | ns | ns | ns | ns | 1.316 | 0.678 | 0.982 | 0.747 | 1.449 | |
| 30 | BABA | 0.07 ± 0.03 | 0.05 ± 0.01 | 0.07 ± 0.02 | 0.08 ± 0.06 | ns | ns | ns | ns | ns | 0.748 | 1.109 | 1.290 | 1.724 | 1.163 | |
| 31 | Betaine | 165.87 ± 44.22 | 159.03 ± 81.25 | 124.17 ± 62.67 | 201.14 ± 66.22 | ns | ns | ns | ns | ns | 0.959 | 0.749 | 1.213 | 1.265 | 1.620 | |
| 32 | Carnosine | 1.44 ± 0.55 | 1.98 ± 1.04 | 0.93 ± 0.46 | 1.05 ± 0.48 | ns | ns | ns | Increased | ns | 1.376 | 0.645 | 0.727 | 0.528 | 1.126 | |
| 33 | Cit | 54.92 ± 8.68 | 64.53 ± 9.05 | 74.72 ± 23.75 | 98.53 ± 23.8 | ns | ns | Increased | Increased | Increased | 1.175 | 1.361 | 1.794 | 1.527 | 1.319 | c |
| 34 | Creatinine | 15.29 ± 3.66 | 15.86 ± 2.96 | 15.25 ± 1.8 | 19.23 ± 5.13 | ns | ns | ns | ns | ns | 1.038 | 0.997 | 1.258 | 1.212 | 1.261 | |
| 35 | Cystine | 10.43 ± 4.84 | 16.77 ± 19.97 | 6.49 ± 3.32 | 8.03 ± 5.74 | ns | ns | ns | ns | ns | 1.608 | 0.623 | 0.770 | 0.479 | 1.236 | |
| 36 | HArg | 0.30 ± 0.06 | 0.32 ± 0.07 | 0.24 ± 0.07 | 0.22 ± 0.07 | ns | ns | Decreased | Increased | ns | 1.044 | 0.796 | 0.725 | 0.695 | 0.911 | |
| 37 | HCys | 14.74 ± 5.65 | 15.76 ± 4.94 | 12.04 ± 4.15 | 16.98 ± 5.67 | ns | ns | ns | ns | ns | 1.069 | 0.817 | 1.152 | 1.078 | 1.411 | |
| 38 | Kynurenine | 1.69 ± 0.1 | 1.88 ± 0.24 | 1.7 ± 0.28 | 2.06 ± 0.22 | ns | ns | Increased | ns | Increased | 1.117 | 1.009 | 1.220 | 1.092 | 1.209 | c |
| 39 | Met-sulfoxide | 2.89 ± 0.84 | 2.60 ± 0.92 | 2.72 ± 1.09 | 4.97 ± 1.85 | ns | ns | Increased | Increased | Increased | 0.901 | 0.941 | 1.719 | 1.909 | 1.828 | c |
| 40 | Orn | 93.65 ± 30.04 | 83.41 ± 30 | 63.63 ± 17.72 | 97.39 ± 23.61 | ns | ns | ns | ns | ns | 0.891 | 0.679 | 1.040 | 1.167 | 1.530 | |
| 41 | PAG | 2.71 ± 2.01 | 1.45 ± 0.75 | 4.1 ± 1.51 | 3.17 ± 2.53 | ns | ns | ns | ns | ns | 0.537 | 1.513 | 1.170 | 2.181 | 0.773 | |
| 42 | ProBetaine | 3.77 ± 0.34 | 3.7 ± 0.33 | 4.78 ± 0.38 | 5.88 ± 1.43 | ns | ns | Increased | Increased | Increased | 0.981 | 1.269 | 1.561 | 1.591 | 1.230 | c |
| 43 | Sarcosine | 2.52 ± 0.92 | 1.85 ± 0.84 | 3.14 ± 1.41 | 3.65 ± 1.58 | ns | ns | ns | ns | ns | 0.737 | 1.248 | 1.452 | 1.971 | 1.164 | |
| 44 | SDMA | 0.27 ± 0.03 | 0.28 ± 0.03 | 0.29 ± 0.04 | 0.29 ± 0.07 | ns | ns | ns | ns | ns | 1.043 | 1.084 | 1.096 | 1.051 | 1.011 | |
| 45 | t4-OH-Pro | 23.7 ± 5.41 | 16.73 ± 3.9 | 10.36 ± 3.47 | 14.14 ± 4.45 | Decreased | Decreased | Decreased | ns | ns | 0.706 | 0.437 | 0.596 | 0.845 | 1.365 | d |
| 46 | Taurine | 444.5 ± 182.17 | 682.14 ± 168.17 | 285.67 ± 142 | 473.14 ± 131.87 | Increased | ns | ns | Decreased | ns | 1.535 | 0.643 | 1.064 | 0.694 | 1.656 | a |
| 47 | CA | 0.19 ± 0.14 | 2.46 ± 3.63 | 0.58 ± 0.31 | 1.61 ± 2.05 | ns | ns | ns | ns | ns | 12.798 | 3.030 | 8.368 | 0.654 | 2.762 | |
| 48 | DCA | 0.19 ± 0.12 | 1.55 ± 1.54 | 0.21 ± 0.09 | 2.4 ± 3.44 | ns | ns | ns | ns | ns | 8.302 | 1.120 | 12.873 | 1.551 | 11.494 | |
| 49 | TCA | 0.29 ± 0.18 | 8.81 ± 11.77 | 0.83 ± 0.6 | 10.65 ± 13.35 | ns | ns | ns | ns | ns | 30.471 | 2.885 | 36.829 | 1.209 | 12.767 | |
| 50 | TDCA | 0.05 ± 0.03 | 0.88 ± 1.16 | 0.07 ± 0.04 | 1.3 ± 1.87 | ns | ns | ns | ns | ns | 18.845 | 1.566 | 27.886 | 1.480 | 17.804 | |
| 51 | TMCA | 0.24 ± 0.19 | 5.76 ± 7.34 | 0.24 ± 0.14 | 7.57 ± 11.10 | ns | ns | ns | ns | ns | 24.498 | 1.001 | 32.169 | 1.313 | 32.123 | |
| 52 | beta-Ala | 1.91 ± 0.31 | 2.11 ± 0.8 | 0.91 ± 0.18 | 1.51 ± 0.71 | ns | Decreased | ns | ns | ns | 1.105 | 0.474 | 0.791 | 0.716 | 1.667 | |
| 53 | GABA | 0.33 ± 0.07 | 0.82 ± 0.81 | 0.33 ± 0.14 | 1.12 ± 1.36 | ns | ns | ns | ns | ns | 2.493 | 1.007 | 3.404 | 1.365 | 3.380 | |
| 54 | Putrescine | 0.95 ± 0.25 | 0.75 ± 0.16 | 0.73 ± 0.09 | 0.99 ± 0.34 | ns | ns | ns | ns | ns | 0.793 | 0.773 | 1.044 | 1.317 | 1.351 | |
| 55 | Spermidine | 1.04 ± 0.37 | 1.10 ± 0.18 | 2.27 ± 1.45 | 2.50 ± 2.39 | ns | ns | ns | ns | ns | 1.063 | 2.195 | 2.418 | 2.275 | 1.102 | |
| 56 | AconAcid | 16.84 ± 7.15 | 17.51 ± 4.15 | 15.5 ± 3.98 | 19.47 ± 5.64 | ns | ns | ns | ns | ns | 1.040 | 0.920 | 1.156 | 1.112 | 1.256 | |
| 57 | HipAcid | 7.66 ± 2.87 | 7.00 ± 3.58 | 37.43 ± 16.41 | 39.73 ± 23.59 | ns | Increased | Increased | Increased | ns | 0.914 | 4.887 | 5.186 | 5.676 | 1.061 | |
| 58 | Lac | 9610 ± 2878.91 | 7950 ± 1638.9 | 9415.5 ± 1030.26 | 13159.71 ± 4318.48 | ns | ns | ns | ns | ns | 0.827 | 0.980 | 1.369 | 1.655 | 1.398 | |
| 59 | OH-GlutAcid | 5.83 ± 0.84 | 6.97 ± 1.2 | 6.95 ± 2.96 | 9.33 ± 3.6 | ns | ns | ns | ns | ns | 1.194 | 1.192 | 1.599 | 1.339 | 1.342 | |
| 60 | Suc | 97.13 ± 114.63 | 54.76 ± 43.89 | 62.57 ± 22.75 | 96.07 ± 88.43 | ns | ns | ns | ns | ns | 0.564 | 0.644 | 0.989 | 1.755 | 1.536 | |
| 61 | p-Cresol-SO4 | 8.87 ± 1.12 | 9.45 ± 3.01 | 12.11 ± 7.29 | 14.98 ± 9.15 | ns | ns | ns | ns | ns | 1.066 | 1.365 | 1.689 | 1.584 | 1.237 | |
| 62 | AA | 4.71 ± 0.91 | 5.14 ± 1.79 | 6.45 ± 2.14 | 6.13 ± 1.87 | ns | ns | ns | ns | ns | 1.092 | 1.370 | 1.302 | 1.193 | 0.950 | |
| 63 | DHA | 20.12 ± 5.21 | 28.16 ± 10.1 | 30.08 ± 5.72 | 28.79 ± 10.43 | ns | ns | ns | ns | ns | 1.400 | 1.495 | 1.431 | 1.022 | 0.957 | |
| 64 | EPA | 1.40 ± 0.54 | 1.67 ± 0.79 | 2.42 ± 0.43 | 2.33 ± 0.72 | ns | Increased | Increased | ns | ns | 1.191 | 1.727 | 1.660 | 1.393 | 0.961 | |
| 65 | FA(12:0) | 194.1 ± 69.72 | 123.26 ± 42.65 | 122.32 ± 38.27 | 146.29 ± 50.57 | ns | ns | ns | ns | ns | 0.635 | 0.630 | 0.754 | 1.187 | 1.196 | |
| 66 | FA(18:1) | 207.17 ± 14.24 | 217.86 ± 16.57 | 208.67 ± 27.29 | 220.71 ± 29.14 | ns | ns | ns | ns | ns | 1.052 | 1.007 | 1.065 | 1.013 | 1.058 | |
| 67 | FA(18:2) | 185.03 ± 69 | 239.71 ± 73.79 | 317 ± 64.86 | 383.29 ± 118.23 | ns | Increased | Increased | Increased | ns | 1.296 | 1.713 | 2.071 | 1.599 | 1.209 | |
| 68 | FA(20:1) | 13.2 ± 8.75 | 13.81 ± 7.58 | 10.77 ± 5.93 | 15.3 ± 12.76 | ns | ns | ns | ns | ns | 1.046 | 0.816 | 1.159 | 1.108 | 1.420 | |
| 69 | FA(20:3) | 1.67 ± 0.8 | 1.98 ± 0.75 | 2.9 ± 1.21 | 2.92 ± 1.64 | ns | ns | ns | ns | ns | 1.181 | 1.732 | 1.741 | 1.475 | 1.006 | |
| 70 | 3-IPA | 8.11 ± 5.55 | 17.61 ± 11.83 | 17.27 ± 14.02 | 24.81 ± 14.86 | ns | ns | ns | ns | ns | 2.171 | 2.129 | 3.059 | 1.409 | 1.437 | |
| 71 | Ind-SO4 | 10.36 ± 3.59 | 11.38 ± 4.3 | 29.6 ± 12.54 | 33.36 ± 9.17 | ns | Increased | Increased | Increased | ns | 1.099 | 2.858 | 3.220 | 2.930 | 1.127 | |
| 72 | Choline | 16.71 ± 8.33 | 16.48 ± 5.73 | 19.35 ± 6.66 | 22.74 ± 8.51 | ns | ns | ns | ns | ns | 0.986 | 1.158 | 1.361 | 1.380 | 1.175 | |
| 73 | C2 | 14.08 ± 2.85 | 14.8 ± 3.77 | 17.5 ± 3.94 | 18.03 ± 3.29 | ns | ns | ns | ns | ns | 1.051 | 1.243 | 1.280 | 1.218 | 1.030 | |
| 74 | C3 | 0.97 ± 0.33 | 0.83 ± 0.25 | 0.93 ± 0.25 | 1.34 ± 0.45 | ns | ns | ns | ns | ns | 0.854 | 0.961 | 1.386 | 1.624 | 1.442 | |
| 75 | C4 | 0.63 ± 0.50 | 0.47 ± 0.23 | 1.05 ± 1.03 | 0.91 ± 0.47 | ns | ns | ns | ns | ns | 0.754 | 1.675 | 1.452 | 1.927 | 0.867 | |
| 76 | C5-DC (C6-OH) | 0.71 ± 0.55 | 0.3 ± 0.05 | 0.24 ± 0.04 | 0.23 ± 0.02 | Decreased | Decreased | Decreased | ns | ns | 0.420 | 0.341 | 0.323 | 0.770 | 0.947 | d |
| 77 | Cer(d18:1/22:0) | 0.64 ± 0.17 | 0.45 ± 0.22 | 0.38 ± 0.1 | 0.25 ± 0.08 | Decreased | Decreased | Decreased | Decreased | ns | 0.701 | 0.600 | 0.392 | 0.560 | 0.653 | d |
| 78 | Cer(d18:1/23:0) | 0.32 ± 0.07 | 0.25 ± 0.08 | 0.3 ± 0.05 | 0.3 ± 0.09 | ns | ns | ns | ns | ns | 0.777 | 0.940 | 0.941 | 1.210 | 1.001 | |
| 79 | Cer(d18:1/24:0) | 0.62 ± 0.18 | 0.41 ± 0.14 | 0.5 ± 0.08 | 0.45 ± 0.15 | ns | ns | ns | ns | ns | 0.652 | 0.805 | 0.725 | 1.112 | 0.901 | |
| 80 | Cer(d18:1/24:1) | 0.86 ± 0.17 | 0.8 ± 0.1 | 0.52 ± 0.07 | 0.49 ± 0.08 | ns | Decreased | Decreased | Decreased | ns | 0.931 | 0.598 | 0.572 | 0.614 | 0.957 | |
| 81 | CE(18:1) | 114.17 ± 29.36 | 95.36 ± 15.26 | 97.28 ± 5.9 | 97.26 ± 35.49 | ns | ns | ns | ns | ns | 0.835 | 0.852 | 0.852 | 1.020 | 1.000 | |
| 82 | CE(18:2) | 842 ± 125.5 | 644.43 ± 181.19 | 1004 ± 121.82 | 758.43 ± 271.62 | ns | ns | ns | ns | Decreased | 0.765 | 1.192 | 0.901 | 1.177 | 0.755 | f |
| 83 | CE(18:3) | 49.35 ± 3.71 | 49.94 ± 9.01 | 57.73 ± 2.54 | 58.7 ± 9.35 | ns | ns | Increased | Increased | ns | 1.012 | 1.170 | 1.189 | 1.175 | 1.017 | |
| 84 | CE(20:1) | 8.13 ± 5.14 | 6.84 ± 0.95 | 7.14 ± 0.96 | 4.03 ± 3.52 | ns | ns | ns | ns | ns | 0.841 | 0.879 | 0.496 | 0.589 | 0.564 | |
| 85 | CE(20:3) | 43.85 ± 7.69 | 39.04 ± 4.69 | 41.78 ± 6.84 | 34.77 ± 3.93 | ns | ns | Decreased | ns | ns | 0.890 | 0.953 | 0.793 | 0.891 | 0.832 | |
| 86 | CE(20:4) | 549.67 ± 94.82 | 382 ± 103.41 | 561 ± 79.11 | 353 ± 85.55 | Decreased | ns | Decreased | ns | Decreased | 0.695 | 1.021 | 0.642 | 0.924 | 0.629 | e |
| 87 | CE(20:5) | 78.2 ± 17.06 | 74.73 ± 22.86 | 106.08 ± 6.63 | 86.87 ± 28.19 | ns | ns | ns | ns | ns | 0.956 | 1.357 | 1.111 | 1.162 | 0.819 | |
| 88 | CE(22:6) | 179.83 ± 37.5 | 137.7 ± 44.76 | 135 ± 20.63 | 86.03 ± 41.47 | ns | ns | Decreased | Decreased | Decreased | 0.766 | 0.751 | 0.478 | 0.625 | 0.637 | f |
| 89 | DG(17:0_18:1) | 2.1 ± 0.42 | 1.69 ± 0.41 | 1.33 ± 0.12 | 1.29 ± 0.43 | ns | Decreased | Decreased | ns | ns | 0.805 | 0.634 | 0.612 | 0.760 | 0.966 | |

**Table S4.** Statistical parameters of 261 qualified metabolites (M261).

| M261 ID | Metabolite | Plasma concentration (µmol $l^{-1}$, mean±SD) | | | | Increased or decreased (*P*<0.05; *ns*, not significant.) | | | | | Fold change | | | | | Group |
|---|---|---|---|---|---|---|---|---|---|---|---|---|---|---|---|---|
| | | WT | *Nrf2*$^{-/-}$ | *Akita* | *Akita::Nrf2*$^{-/-}$ | *Nrf2*$^{-/-}$ *vs.* WT | *Akita vs.* WT | *Akita::Nrf2*$^{-/-}$ *vs.* WT | *Akita::Nrf2*$^{-/-}$ *vs. Nrf2*$^{-/-}$ | *Akita::Nrf2*$^{-/-}$ *vs. Akita* | *Nrf2*$^{-/-}$ *vs.* WT | *Akita vs.* WT | *Akita::Nrf2*$^{-/-}$ *vs.* WT | *Akita::Nrf2*$^{-/-}$ *vs. Nrf2*$^{-/-}$ | *Akita::Nrf2*$^{-/-}$ *vs. Akita* | |
| 90 | DG(18:1_20:2) | 1.29 ± 0.61 | 0.96 ± 0.46 | 1 ± 0.09 | 0.71 ± 0.48 | ns | ns | ns | ns | ns | 0.741 | 0.777 | 0.550 | 0.743 | 0.708 | |
| 91 | lysoPC a C14:0 | 1.32 ± 0.21 | 1.5 ± 0.15 | 0.9 ± 0.14 | 0.9 ± 0.1 | ns | Decreased | Decreased | Decreased | ns | 1.137 | 0.678 | 0.683 | 0.601 | 1.008 | |
| 92 | lysoPC a C16:0 | 94.55 ± 15.55 | 88.67 ± 17.3 | 58.92 ± 8.21 | 56.46 ± 16 | ns | Decreased | Decreased | Decreased | ns | 0.938 | 0.623 | 0.597 | 0.637 | 0.958 | |
| 93 | lysoPC a C16:1 | 2.65 ± 1.08 | 3.57 ± 0.51 | 0.95 ± 0.15 | 1.09 ± 0.27 | Increased | Decreased | Decreased | Decreased | ns | 1.346 | 0.358 | 0.411 | 0.305 | 1.150 | a |
| 94 | lysoPC a C17:0 | 1.84 ± 0.25 | 1.55 ± 0.33 | 1.87 ± 0.15 | 1.72 ± 0.43 | ns | ns | ns | ns | ns | 0.842 | 1.016 | 0.933 | 1.109 | 0.918 | |
| 95 | lysoPC a C18:0 | 40.83 ± 4.51 | 36.8 ± 8.71 | 46.02 ± 8.16 | 40.17 ± 14.67 | ns | ns | ns | ns | ns | 0.901 | 1.127 | 0.984 | 1.092 | 0.873 | |
| 96 | lysoPC a C18:1 | 22.37 ± 5.67 | 27.37 ± 3.2 | 12.52 ± 2.01 | 13.04 ± 2.71 | Increased | Decreased | Decreased | Decreased | ns | 1.224 | 0.560 | 0.583 | 0.477 | 1.042 | a |
| 97 | lysoPC a C18:2 | 51.7 ± 8.42 | 59.61 ± 5.5 | 46.97 ± 7.34 | 58.07 ± 8.63 | ns | ns | ns | ns | Increased | 1.153 | 0.908 | 1.123 | 0.974 | 1.236 | c |
| 98 | lysoPC a C20:3 | 3.58 ± 0.96 | 4.35 ± 0.76 | 2.16 ± 0.72 | 2.01 ± 0.32 | ns | Decreased | Decreased | Decreased | ns | 1.215 | 0.603 | 0.562 | 0.463 | 0.933 | |
| 99 | lysoPC a C20:4 | 13.78 ± 2.18 | 16.61 ± 2.9 | 10.13 ± 3.04 | 10.22 ± 3.05 | ns | ns | Decreased | Decreased | ns | 1.205 | 0.735 | 0.742 | 0.615 | 1.009 | |
| 100 | lysoPC a C24:0 | 0.38 ± 0.07 | 0.31 ± 0.08 | 0.25 ± 0.04 | 0.22 ± 0.04 | Decreased | Decreased | Decreased | Decreased | ns | 0.804 | 0.660 | 0.565 | 0.703 | 0.855 | d |
| 101 | PC aa C28:1 | 0.12 ± 0.02 | 0.1 ± 0.01 | 0.11 ± 0.01 | 0.1 ± 0.02 | ns | ns | ns | ns | ns | 0.889 | 0.970 | 0.874 | 0.983 | 0.902 | |
| 102 | PC aa C30:0 | 0.77 ± 0.09 | 0.71 ± 0.04 | 0.56 ± 0.07 | 0.59 ± 0.05 | ns | Decreased | Decreased | Decreased | ns | 0.933 | 0.735 | 0.773 | 0.828 | 1.052 | |
| 103 | PC aa C32:0 | 10.79 ± 1.41 | 9.09 ± 1.32 | 6.84 ± 0.96 | 6.76 ± 0.75 | Decreased | Decreased | Decreased | Decreased | ns | 0.843 | 0.634 | 0.626 | 0.743 | 0.988 | d |
| 104 | PC aa C32:1 | 4.51 ± 1.69 | 4.54 ± 1.07 | 1.35 ± 0.09 | 1.45 ± 0.53 | ns | Decreased | Decreased | Decreased | ns | 1.006 | 0.298 | 0.321 | 0.319 | 1.076 | |
| 105 | PC aa C32:2 | 1.36 ± 0.35 | 1.26 ± 0.14 | 0.92 ± 0.14 | 1.04 ± 0.23 | ns | Decreased | Decreased | ns | ns | 0.921 | 0.671 | 0.765 | 0.830 | 1.140 | |
| 106 | PC aa C32:3 | 0.18 ± 0.03 | 0.16 ± 0.03 | 0.19 ± 0.03 | 0.18 ± 0.04 | ns | ns | ns | ns | ns | 0.846 | 1.042 | 0.999 | 1.181 | 0.958 | |
| 107 | PC aa C34:1 | 97.2 ± 23.45 | 97.07 ± 24.79 | 46.47 ± 8.85 | 39.27 ± 9.8 | ns | Decreased | Decreased | Decreased | ns | 0.999 | 0.478 | 0.404 | 0.405 | 0.845 | |
| 108 | PC aa C34:2 | 410 ± 58.07 | 319.57 ± 93.24 | 374.17 ± 65.6 | 381 ± 121.64 | ns | ns | ns | ns | ns | 0.779 | 0.913 | 0.929 | 1.192 | 1.018 | |
| 109 | PC aa C34:3 | 7.77 ± 1.59 | 7.31 ± 1.93 | 5.43 ± 0.6 | 5.55 ± 1.4 | ns | Decreased | Decreased | ns | ns | 0.941 | 0.699 | 0.715 | 0.760 | 1.022 | |
| 110 | PC aa C34:4 | 0.34 ± 0.07 | 0.34 ± 0.06 | 0.29 ± 0.03 | 0.33 ± 0.09 | ns | ns | ns | ns | ns | 0.993 | 0.852 | 0.960 | 0.967 | 1.128 | |
| 111 | PC aa C36:0 | 2.24 ± 0.44 | 1.85 ± 0.46 | 2.43 ± 0.34 | 2.09 ± 0.6 | ns | ns | ns | ns | ns | 0.825 | 1.081 | 0.934 | 1.132 | 0.864 | |
| 112 | PC aa C36:1 | 15.1 ± 2.42 | 16.27 ± 3.95 | 13.47 ± 2.16 | 11.16 ± 2.42 | ns | ns | Decreased | Decreased | ns | 1.078 | 0.892 | 0.739 | 0.686 | 0.829 | |
| 113 | PC aa C36:2 | 167 ± 16.6 | 146.43 ± 26.4 | 263.33 ± 50.17 | 258.57 ± 75.17 | ns | Increased | Increased | Increased | ns | 0.877 | 1.577 | 1.548 | 1.766 | 0.982 | |
| 114 | PC aa C36:3 | 66.83 ± 9.03 | 61.16 ± 4.77 | 63.67 ± 5.98 | 67 ± 17.36 | ns | ns | ns | ns | ns | 0.915 | 0.953 | 1.002 | 1.096 | 1.052 | |
| 115 | PC aa C36:4 | 111.3 ± 14.65 | 88.57 ± 22.57 | 88.38 ± 14.79 | 84.26 ± 20.57 | ns | ns | ns | ns | ns | 0.796 | 0.794 | 0.757 | 0.951 | 0.953 | |
| 116 | PC aa C36:5 | 7.17 ± 1.06 | 6.95 ± 1.32 | 7.21 ± 0.79 | 8.48 ± 2.23 | ns | ns | ns | ns | ns | 0.970 | 1.007 | 1.184 | 1.220 | 1.176 | |
| 117 | PC aa C36:6 | 0.25 ± 0.07 | 0.25 ± 0.04 | 0.15 ± 0.01 | 0.2 ± 0.06 | ns | Decreased | ns | ns | ns | 0.999 | 0.611 | 0.800 | 0.801 | 1.309 | |
| 118 | PC aa C38:0 | 1.43 ± 0.2 | 1.26 ± 0.3 | 1.53 ± 0.26 | 1.3 ± 0.34 | ns | ns | ns | ns | ns | 0.883 | 1.073 | 0.914 | 1.035 | 0.852 | |
| 119 | PC aa C38:3 | 23.23 ± 2.87 | 21.26 ± 3.73 | 24.2 ± 4.53 | 20.59 ± 4.68 | ns | ns | ns | ns | ns | 0.915 | 1.042 | 0.886 | 0.968 | 0.851 | |
| 120 | PC aa C38:4 | 61.05 ± 8.85 | 50.76 ± 12.02 | 87.33 ± 21.81 | 66.94 ± 15.11 | ns | Increased | ns | ns | Decreased | 0.831 | 1.431 | 1.097 | 1.319 | 0.767 | f |
| 121 | PC aa C38:5 | 20.95 ± 3.25 | 20.21 ± 2.06 | 18.85 ± 1.91 | 17.43 ± 4.18 | ns | ns | ns | ns | ns | 0.965 | 0.900 | 0.832 | 0.862 | 0.925 | |
| 122 | PC aa C38:6 | 104.35 ± 16.77 | 92.54 ± 29.13 | 67.22 ± 12.36 | 51.87 ± 14.45 | ns | Decreased | Decreased | Decreased | ns | 0.887 | 0.644 | 0.497 | 0.561 | 0.772 | |
| 123 | PC aa C40:1 | 0.44 ± 0.02 | 0.44 ± 0.03 | 0.42 ± 0.02 | 0.43 ± 0.04 | ns | ns | ns | ns | ns | 1.006 | 0.949 | 0.990 | 0.984 | 1.042 | |
| 124 | PC aa C40:2 | 0.42 ± 0.06 | 0.39 ± 0.11 | 0.33 ± 0.05 | 0.32 ± 0.06 | Decreased | ns | ns | ns | ns | 0.918 | 0.768 | 0.758 | 0.826 | 0.987 | d |
| 125 | PC aa C40:3 | 0.78 ± 0.12 | 0.69 ± 0.18 | 0.54 ± 0.07 | 0.54 ± 0.1 | ns | Decreased | Decreased | ns | ns | 0.884 | 0.691 | 0.691 | 0.782 | 1.000 | |
| 126 | PC aa C40:4 | 2.99 ± 0.4 | 2.4 ± 0.83 | 2.91 ± 0.64 | 2.31 ± 0.61 | ns | ns | ns | ns | ns | 0.802 | 0.972 | 0.774 | 0.965 | 0.797 | |
| 127 | PC aa C40:5 | 3.16 ± 0.61 | 3.04 ± 0.5 | 2.89 ± 0.69 | 2.33 ± 0.52 | ns | ns | ns | ns | ns | 0.963 | 0.915 | 0.739 | 0.767 | 0.807 | |
| 128 | PC aa C40:6 | 27.7 ± 4.58 | 25.39 ± 8.46 | 38.88 ± 9.37 | 30.13 ± 11.85 | ns | ns | ns | ns | ns | 0.916 | 1.404 | 1.088 | 1.187 | 0.775 | |
| 129 | PC aa C42:0 | 0.09 ± 0.02 | 0.09 ± 0.01 | 0.1 ± 0.02 | 0.1 ± 0.02 | ns | ns | ns | ns | ns | 0.986 | 1.147 | 1.119 | 1.134 | 0.975 | |
| 130 | PC aa C42:1 | 0.1 ± 0.02 | 0.1 ± 0.01 | 0.11 ± 0.03 | 0.11 ± 0.02 | ns | ns | ns | ns | ns | 0.985 | 1.077 | 1.082 | 1.099 | 1.005 | |
| 131 | PC aa C42:2 | 0.29 ± 0.06 | 0.23 ± 0.05 | 0.32 ± 0.04 | 0.35 ± 0.09 | ns | ns | ns | Increased | ns | 0.794 | 1.085 | 1.190 | 1.499 | 1.096 | |
| 132 | PC aa C42:4 | 0.22 ± 0.03 | 0.19 ± 0.06 | 0.17 ± 0.04 | 0.17 ± 0.03 | ns | ns | ns | ns | ns | 0.850 | 0.779 | 0.763 | 0.897 | 0.979 | |
| 133 | PC aa C42:5 | 0.3 ± 0.03 | 0.27 ± 0.07 | 0.22 ± 0.03 | 0.21 ± 0.04 | ns | Decreased | Decreased | Decreased | ns | 0.916 | 0.737 | 0.716 | 0.781 | 0.972 | |
| 134 | PC aa C42:6 | 1.3 ± 0.22 | 1.11 ± 0.47 | 1.14 ± 0.24 | 0.92 ± 0.26 | ns | ns | ns | ns | ns | 0.857 | 0.882 | 0.706 | 0.824 | 0.800 | |
| 135 | PC ae C30:0 | 0.11 ± 0.01 | 0.1 ± 0.01 | 0.09 ± 0.01 | 0.09 ± 0.01 | ns | Decreased | Decreased | Decreased | ns | 0.988 | 0.835 | 0.842 | 0.853 | 1.009 | |
| 136 | PC ae C30:1 | 0.08 ± 0.02 | 0.06 ± 0.02 | 0.07 ± 0.01 | 0.08 ± 0.02 | ns | ns | ns | ns | ns | 0.796 | 0.862 | 0.973 | 1.222 | 1.129 | |
| 137 | PC ae C30:2 | 0.19 ± 0.03 | 0.15 ± 0.03 | 0.19 ± 0.04 | 0.14 ± 0.03 | Decreased | ns | Decreased | ns | Decreased | 0.776 | 0.973 | 0.716 | 0.922 | 0.736 | e |
| 138 | PC ae C32:1 | 0.49 ± 0.08 | 0.44 ± 0.05 | 0.39 ± 0.07 | 0.35 ± 0.07 | ns | Decreased | Decreased | Decreased | ns | 0.896 | 0.800 | 0.716 | 0.799 | 0.894 | |
| 139 | PC ae C32:2 | 0.24 ± 0.05 | 0.22 ± 0.03 | 0.24 ± 0.03 | 0.22 ± 0.04 | ns | ns | ns | ns | ns | 0.909 | 0.975 | 0.916 | 1.008 | 0.939 | |
| 140 | PC ae C34:0 | 1.01 ± 0.25 | 0.9 ± 0.19 | 1.18 ± 0.19 | 1.14 ± 0.33 | ns | ns | ns | ns | ns | 0.887 | 1.165 | 1.124 | 1.268 | 0.965 | |
| 141 | PC ae C34:1 | 2.84 ± 0.3 | 2.55 ± 0.36 | 1.91 ± 0.19 | 1.68 ± 0.31 | ns | Decreased | Decreased | Decreased | ns | 0.898 | 0.673 | 0.593 | 0.660 | 0.880 | |
| 142 | PC ae C34:2 | 3.83 ± 0.65 | 3.04 ± 0.79 | 4 ± 0.88 | 4.3 ± 1.16 | ns | ns | ns | ns | ns | 0.793 | 1.043 | 1.123 | 1.417 | 1.077 | |
| 143 | PC ae C34:3 | 0.93 ± 0.16 | 0.76 ± 0.18 | 1.08 ± 0.17 | 0.89 ± 0.25 | ns | ns | ns | ns | ns | 0.813 | 1.160 | 0.964 | 1.185 | 0.831 | |
| 144 | PC ae C36:0 | 0.6 ± 0.16 | 0.56 ± 0.13 | 0.71 ± 0.14 | 0.69 ± 0.21 | ns | ns | ns | ns | ns | 0.936 | 1.184 | 1.154 | 1.233 | 0.975 | |
| 145 | PC ae C36:1 | 5.44 ± 0.79 | 5.11 ± 0.87 | 4.59 ± 0.4 | 4.24 ± 1.03 | ns | ns | Decreased | ns | ns | 0.940 | 0.843 | 0.780 | 0.830 | 0.925 | |
| 146 | PC ae C36:2 | 8.04 ± 1.13 | 6.35 ± 1.16 | 11.71 ± 2.39 | 11.6 ± 3.54 | ns | Increased | Increased | Increased | ns | 0.790 | 1.457 | 1.443 | 1.826 | 0.990 | |
| 147 | PC ae C36:3 | 2.06 ± 0.2 | 1.71 ± 0.38 | 2.28 ± 0.23 | 2.32 ± 0.58 | ns | ns | ns | Increased | ns | 0.830 | 1.108 | 1.127 | 1.358 | 1.018 | |
| 148 | PC ae C36:4 | 2.9 ± 0.44 | 2.12 ± 0.5 | 3.09 ± 0.71 | 2.63 ± 0.71 | Decreased | ns | ns | ns | ns | 0.732 | 1.068 | 0.907 | 1.240 | 0.850 | d |
| 149 | PC ae C36:5 | 1.55 ± 0.21 | 1.2 ± 0.33 | 1.84 ± 0.39 | 1.5 ± 0.47 | ns | ns | ns | ns | ns | 0.774 | 1.187 | 0.966 | 1.249 | 0.814 | |
| 150 | PC ae C38:0 | 1.54 ± 0.38 | 1.53 ± 0.32 | 1.02 ± 0.07 | 1.08 ± 0.25 | ns | Decreased | Decreased | Decreased | ns | 0.995 | 0.659 | 0.697 | 0.701 | 1.059 | |
| 151 | PC ae C38:1 | 2.18 ± 0.33 | 2.17 ± 0.34 | 1.77 ± 0.15 | 1.82 ± 0.27 | ns | Decreased | Decreased | Decreased | ns | 0.994 | 0.809 | 0.833 | 0.838 | 1.030 | |
| 152 | PC ae C38:2 | 8.39 ± 0.82 | 7.17 ± 1.62 | 7.53 ± 1.13 | 6.7 ± 1.38 | ns | ns | ns | ns | ns | 0.854 | 0.897 | 0.798 | 0.934 | 0.889 | |
| 153 | PC ae C38:3 | 3.01 ± 0.38 | 2.66 ± 0.32 | 2.92 ± 0.34 | 2.7 ± 0.52 | ns | ns | ns | ns | ns | 0.886 | 0.970 | 0.899 | 1.014 | 0.927 | |
| 154 | PC ae C38:4 | 3.29 ± 0.6 | 2.52 ± 0.59 | 4.07 ± 0.69 | 3.5 ± 0.93 | ns | ns | ns | Increased | ns | 0.766 | 1.238 | 1.067 | 1.392 | 0.862 | |
| 155 | PC ae C38:5 | 2.38 ± 0.33 | 1.96 ± 0.4 | 2.86 ± 0.52 | 2.44 ± 0.61 | ns | ns | ns | ns | ns | 0.824 | 1.206 | 1.026 | 1.245 | 0.851 | |
| 156 | PC ae C38:6 | 1.73 ± 0.27 | 1.42 ± 0.37 | 1.86 ± 0.38 | 1.67 ± 0.55 | ns | ns | ns | ns | ns | 0.821 | 1.075 | 0.965 | 1.175 | 0.898 | |
| 157 | PC ae C40:1 | 1.86 ± 0.4 | 1.53 ± 0.51 | 1.63 ± 0.22 | 1.43 ± 0.32 | ns | ns | ns | ns | ns | 0.825 | 0.881 | 0.773 | 0.937 | 0.878 | |
| 158 | PC ae C40:2 | 0.89 ± 0.17 | 0.74 ± 0.21 | 0.74 ± 0.1 | 0.68 ± 0.15 | ns | ns | ns | ns | ns | 0.825 | 0.827 | 0.766 | 0.928 | 0.927 | |
| 159 | PC ae C40:3 | 0.98 ± 0.14 | 0.87 ± 0.15 | 0.86 ± 0.1 | 0.84 ± 0.15 | ns | ns | ns | ns | ns | 0.890 | 0.876 | 0.860 | 0.967 | 0.983 | |
| 160 | PC ae C40:4 | 1.91 ± 0.25 | 1.61 ± 0.4 | 1.79 ± 0.28 | 1.56 ± 0.29 | ns | ns | ns | ns | ns | 0.846 | 0.939 | 0.820 | 0.970 | 0.874 | |
| 161 | PC ae C40:5 | 1.57 ± 0.33 | 1.52 ± 0.27 | 2.03 ± 0.25 | 2.01 ± 0.59 | ns | ns | ns | ns | ns | 0.964 | 1.292 | 1.282 | 1.329 | 0.992 | |
| 162 | PC ae C40:6 | 2.4 ± 0.49 | 2.01 ± 0.51 | 3.03 ± 0.4 | 2.5 ± 0.8 | ns | ns | ns | ns | ns | 0.838 | 1.261 | 1.040 | 1.241 | 0.825 | |
| 163 | PC ae C42:0 | 0.81 ± 0.13 | 0.74 ± 0.12 | 0.5 ± 0.05 | 0.43 ± 0.04 | ns | ns | Decreased | ns | ns | 0.917 | 0.615 | 0.526 | 0.574 | 0.856 | |
| 164 | PC ae C42:1 | 0.36 ± 0.05 | 0.31 ± 0.07 | 0.29 ± 0.03 | 0.28 ± 0.04 | ns | ns | ns | ns | ns | 0.884 | 0.814 | 0.781 | 0.883 | 0.959 | |
| 165 | PC ae C42:2 | 0.34 ± 0.09 | 0.29 ± 0.08 | 0.28 ± 0.05 | 0.27 ± 0.07 | ns | ns | ns | ns | ns | 0.846 | 0.823 | 0.795 | 0.941 | 0.966 | |
| 166 | PC ae C42:3 | 1.14 ± 0.27 | 0.91 ± 0.28 | 0.7 ± 0.1 | 0.68 ± 0.14 | ns | Decreased | Decreased | ns | ns | 0.797 | 0.614 | 0.600 | 0.752 | 0.978 | |
| 167 | PC ae C42:5 | 0.9 ± 0.08 | 0.89 ± 0.07 | 0.93 ± 0.08 | 0.94 ± 0.1 | ns | ns | ns | ns | ns | 0.988 | 1.032 | 1.040 | 1.053 | 1.008 | |
| 168 | PC ae C44:3 | 0.06 ± 0.01 | 0.06 ± 0.01 | 0.06 ± 0.01 | 0.06 ± 0.03 | ns | ns | ns | ns | ns | 1.063 | 1.019 | 1.054 | 0.991 | 1.034 | |
| 169 | PC ae C44:4 | 0.09 ± 0.01 | 0.09 ± 0.01 | 0.1 ± 0.01 | 0.11 ± 0.01 | ns | ns | Increased | Increased | ns | 0.964 | 1.112 | 1.193 | 1.238 | 1.073 | |
| 170 | PC ae C44:5 | 0.16 ± 0.03 | 0.13 ± 0.03 | 0.12 ± 0.02 | 0.14 ± 0.03 | ns | ns | ns | ns | ns | 0.828 | 0.787 | 0.856 | 1.034 | 1.087 | |
| 171 | PC ae C44:6 | 0.19 ± 0.02 | 0.19 ± 0.02 | 0.22 ± 0.03 | 0.21 ± 0.02 | ns | ns | ns | ns | ns | 0.958 | 1.114 | 1.089 | 1.137 | 0.978 | |
| 172 | SM (OH) C14:1 | 0.93 ± 0.12 | 0.79 ± 0.15 | 1.05 ± 0.12 | 0.76 ± 0.18 | ns | ns | ns | ns | Decreased | 0.853 | 1.138 | 0.819 | 0.961 | 0.720 | f |
| 173 | SM (OH) C16:1 | 0.59 ± 0.07 | 0.48 ± 0.08 | 0.64 ± 0.1 | 0.5 ± 0.13 | ns | ns | ns | ns | Decreased | 0.818 | 1.075 | 0.847 | 1.035 | 0.787 | f |
| 174 | SM (OH) C22:1 | 2.42 ± 0.49 | 1.58 ± 0.64 | 3.1 ± 0.7 | 2.12 ± 0.53 | Decreased | ns | ns | ns | Decreased | 0.654 | 1.279 | 0.875 | 1.338 | 0.684 | e |
| 175 | SM (OH) C22:2 | 5.16 ± 0.47 | 4.12 ± 1.02 | 4.25 ± 0.71 | 3.59 ± 0.7 | Decreased | ns | Decreased | ns | ns | 0.798 | 0.824 | 0.696 | 0.872 | 0.844 | d |
| 176 | SM (OH) C24:1 | 2.32 ± 0.44 | 1.74 ± 0.57 | 2.04 ± 0.2 | 1.74 ± 0.42 | ns | ns | ns | ns | ns | 0.751 | 0.881 | 0.749 | 0.998 | 0.851 | |
| 177 | SM C16:0 | 19.32 ± 2.56 | 15.24 ± 1.89 | 21.75 ± 3.56 | 17.04 ± 4.13 | Decreased | ns | ns | ns | Decreased | 0.789 | 1.126 | 0.882 | 1.118 | 0.784 | e |
| 178 | SM C16:1 | 3.2 ± 0.45 | 1.98 ± 0.71 | 2.98 ± 0.41 | 2.01 ± 0.83 | Decreased | ns | Decreased | ns | Decreased | 0.619 | 0.929 | 0.627 | 1.012 | 0.675 | e |

**Table S4.** Statistical parameters of 261 qualified metabolites (M261).

| M261 ID | Metabolite | Plasma concentration (μmol $l^{-1}$, mean±SD) | | | | Increased or decreased (*P*<0.05; *ns*, not significant.) | | | | | Fold change | | | | | Group |
|---|---|---|---|---|---|---|---|---|---|---|---|---|---|---|---|---|
| | | WT | *Nrf2*$^{-/-}$ | *Akita* | *Akita::Nrf2*$^{-/-}$ | *Nrf2*$^{-/-}$ *vs.* WT | *Akita vs.* WT | *Akita::Nrf2*$^{-/-}$ *vs.* WT | *Akita::Nrf2*$^{-/-}$ *vs. Nrf2*$^{-/-}$ | *Akita::Nrf2*$^{-/-}$ *vs. Akita* | *Nrf2*$^{-/-}$ *vs.* WT | *Akita vs.* WT | *Akita::Nrf2*$^{-/-}$ *vs.* WT | *Akita::Nrf2*$^{-/-}$ *vs. Nrf2*$^{-/-}$ | *Akita::Nrf2*$^{-/-}$ *vs. Akita* | |
| 179 | SM C18:0 | 4.46 ± 1.11 | 4.11 ± 0.82 | 2.66 ± 0.52 | 2.5 ± 0.76 | ns | Decreased | Decreased | Decreased | ns | 0.922 | 0.596 | 0.561 | 0.608 | 0.940 | |
| 180 | SM C18:1 | 1.63 ± 0.37 | 1.37 ± 0.22 | 1.22 ± 0.2 | 1.25 ± 0.33 | ns | ns | ns | ns | ns | 0.838 | 0.744 | 0.766 | 0.914 | 1.029 | |
| 181 | SM C20:2 | 9.05 ± 1.89 | 8.18 ± 2.34 | 6.15 ± 0.75 | 6.26 ± 1.80 | ns | Decreased | Decreased | ns | ns | 0.904 | 0.680 | 0.692 | 0.765 | 1.018 | |
| 182 | SM C24:0 | 10.37 ± 1.83 | 7.03 ± 2.51 | 9.66 ± 1.79 | 6.64 ± 1.54 | Decreased | ns | Decreased | ns | Decreased | 0.678 | 0.932 | 0.641 | 0.945 | 0.688 | e |
| 183 | SM C24:1 | 20.77 ± 3.53 | 15 ± 3.24 | 14.55 ± 1.88 | 10.69 ± 1.83 | Decreased | Decreased | Decreased | Decreased | Decreased | 0.722 | 0.701 | 0.515 | 0.713 | 0.735 | e |
| 184 | SM C26:0 | 0.25 ± 0.03 | 0.22 ± 0.02 | 0.24 ± 0.02 | 0.24 ± 0.03 | ns | ns | ns | ns | ns | 0.882 | 0.989 | 0.983 | 1.116 | 0.994 | |
| 185 | SM C26:1 | 0.13 ± 0.06 | 0.09 ± 0.03 | 0.13 ± 0.03 | 0.14 ± 0.06 | ns | ns | ns | ns | ns | 0.660 | 0.975 | 1.030 | 1.560 | 1.056 | |
| 186 | TG(16:0_34:2) | 13.46 ± 5.85 | 14.2 ± 6.79 | 17.83 ± 8.59 | 43.81 ± 21.41 | ns | ns | Increased | Increased | Increased | 1.055 | 1.324 | 3.254 | 3.086 | 2.458 | c |
| 187 | TG(16:0_34:3) | 7.14 ± 3.55 | 6.24 ± 2.73 | 3.47 ± 1.48 | 8.16 ± 4.27 | ns | ns | ns | ns | ns | 0.874 | 0.486 | 1.143 | 1.308 | 2.353 | |
| 188 | TG(16:0_36:2) | 17.83 ± 7.61 | 17.06 ± 8.84 | 19.6 ± 9.6 | 36.38 ± 23.31 | ns | ns | ns | ns | ns | 0.957 | 1.099 | 2.041 | 2.132 | 1.856 | |
| 189 | TG(16:0_36:3) | 42.95 ± 14.9 | 34.41 ± 15.05 | 58.7 ± 26.11 | 104.24 ± 53.09 | ns | ns | Increased | Increased | Increased | 0.801 | 1.367 | 2.427 | 3.030 | 1.776 | c |
| 190 | TG(16:0_36:4) | 26.73 ± 9.8 | 19.88 ± 11.53 | 50.72 ± 21.93 | 94.04 ± 45.58 | ns | ns | Increased | Increased | Increased | 0.744 | 1.897 | 3.518 | 4.731 | 1.854 | c |
| 191 | TG(16:0_36:5) | 3.27 ± 1.19 | 2.74 ± 1.48 | 5.51 ± 2.36 | 12.14 ± 5.9 | ns | ns | Increased | Increased | Increased | 0.837 | 1.682 | 3.710 | 4.431 | 2.205 | c |
| 192 | TG(16:0_38:4) | 1.17 ± 0.44 | 1.03 ± 0.33 | 1.3 ± 0.76 | 2.41 ± 1.18 | ns | ns | Increased | Increased | Increased | 0.882 | 1.113 | 2.071 | 2.347 | 1.861 | c |
| 193 | TG(16:0_38:5) | 2.34 ± 0.54 | 2.09 ± 0.54 | 2.9 ± 0.92 | 4.15 ± 1.69 | ns | ns | Increased | Increased | ns | 0.892 | 1.242 | 1.773 | 1.988 | 1.428 | |
| 194 | TG(16:0_40:8) | 2.73 ± 1.12 | 2.32 ± 0.98 | 3.57 ± 1.83 | 4.94 ± 2.29 | ns | ns | ns | ns | ns | 0.848 | 1.308 | 1.808 | 2.131 | 1.382 | |
| 195 | TG(16:1_34:2) | 6.95 ± 4.04 | 6.2 ± 3.29 | 2.42 ± 1.12 | 5.28 ± 3.39 | ns | ns | ns | ns | ns | 0.893 | 0.348 | 0.759 | 0.851 | 2.182 | |
| 196 | TG(16:1_36:2) | 3.58 ± 2.14 | 3.71 ± 1.89 | 2.5 ± 1.23 | 4.95 ± 4.70 | ns | ns | ns | ns | ns | 1.036 | 0.699 | 1.384 | 1.336 | 1.981 | |
| 197 | TG(16:1_36:3) | 7.1 ± 3.23 | 6.2 ± 2.43 | 6.45 ± 2.51 | 10.78 ± 7.30 | ns | ns | ns | ns | ns | 0.873 | 0.909 | 1.517 | 1.737 | 1.670 | |
| 198 | TG(16:1_36:4) | 3.19 ± 1.19 | 2.83 ± 1.72 | 4.65 ± 1.70 | 7.46 ± 3.86 | ns | ns | Increased | Increased | ns | 0.887 | 1.457 | 2.336 | 2.632 | 1.603 | |
| 199 | TG(18:0_36:3) | 2.19 ± 0.64 | 2.21 ± 0.84 | 8.29 ± 3.81 | 20.08 ± 10.95 | ns | ns | Increased | Increased | Increased | 1.007 | 3.782 | 9.161 | 9.095 | 2.422 | c |
| 200 | TG(18:0_36:4) | 1.62 ± 0.45 | 1.89 ± 0.96 | 8.80 ± 4.30 | 20.60 ± 10.75 | ns | ns | Increased | Increased | Increased | 1.164 | 5.415 | 12.682 | 10.894 | 2.342 | c |
| 201 | TG(18:0_36:5) | 0.48 ± 0.1 | 0.48 ± 0.18 | 0.89 ± 0.56 | 2.61 ± 1.21 | ns | ns | Increased | Increased | Increased | 1.012 | 1.857 | 5.474 | 5.412 | 2.947 | c |
| 202 | TG(18:1_32:1) | 5.9 ± 3.65 | 5.97 ± 4.11 | 1.79 ± 0.76 | 4.76 ± 4.00 | ns | ns | ns | ns | ns | 1.011 | 0.304 | 0.807 | 0.798 | 2.658 | |
| 203 | TG(18:1_33:2) | 0.72 ± 0.24 | 0.53 ± 0.39 | 0.96 ± 0.51 | 2.41 ± 1.37 | ns | ns | Increased | Increased | Increased | 0.736 | 1.338 | 3.350 | 4.554 | 2.503 | c |
| 204 | TG(18:1_34:1) | 33.67 ± 14.71 | 33.37 ± 17.45 | 33.37 ± 17.84 | 63.46 ± 43.01 | ns | ns | ns | ns | ns | 0.991 | 0.991 | 1.885 | 1.902 | 1.902 | |
| 205 | TG(18:1_34:2) | 42.33 ± 15.06 | 34.74 ± 13.77 | 53.97 ± 23.61 | 100.01 ± 53.48 | ns | ns | Increased | Increased | Increased | 0.821 | 1.275 | 2.363 | 2.879 | 1.853 | c |
| 206 | TG(18:1_34:3) | 9.29 ± 4.24 | 7.47 ± 3.08 | 9.27 ± 3.49 | 18.11 ± 10.78 | ns | ns | ns | ns | ns | 0.804 | 0.998 | 1.950 | 2.425 | 1.954 | |
| 207 | TG(18:1_35:2) | 0.96 ± 0.24 | 0.85 ± 0.33 | 1.58 ± 0.65 | 3.40 ± 2.13 | ns | ns | Increased | Increased | Increased | 0.887 | 1.639 | 3.533 | 3.983 | 2.156 | c |
| 208 | TG(18:1_35:3) | 0.49 ± 0.11 | 0.31 ± 0.22 | 0.56 ± 0.30 | 1.28 ± 0.75 | ns | ns | ns | ns | ns | 0.643 | 1.147 | 2.648 | 4.119 | 2.309 | |
| 209 | TG(18:1_36:1) | 1.93 ± 0.71 | 2.14 ± 0.81 | 4.6 ± 2.29 | 10.54 ± 7.04 | ns | ns | Increased | Increased | Increased | 1.105 | 2.376 | 5.450 | 4.932 | 2.294 | c |
| 210 | TG(18:1_36:2) | 8.78 ± 3.55 | 9.9 ± 3.76 | 19.8 ± 8.57 | 42.61 ± 30.12 | ns | ns | Increased | Increased | Increased | 1.128 | 2.256 | 4.855 | 4.306 | 2.152 | c |
| 211 | TG(18:1_36:3) | 18.16 ± 6.22 | 17.23 ± 5.19 | 47.93 ± 18.86 | 100.67 ± 54.72 | ns | ns | Increased | Increased | Increased | 0.949 | 2.639 | 5.543 | 5.843 | 2.100 | c |
| 212 | TG(18:1_36:4) | 10.3 ± 3.03 | 9.44 ± 4.51 | 37.1 ± 16.32 | 83.10 ± 37.08 | ns | ns | Increased | Increased | Increased | 0.916 | 3.601 | 8.065 | 8.807 | 2.240 | c |
| 213 | TG(18:1_36:5) | 2.5 ± 0.62 | 2.18 ± 0.95 | 6.29 ± 2.54 | 14.06 ± 6.41 | ns | ns | Increased | Increased | Increased | 0.874 | 2.517 | 5.622 | 6.435 | 2.233 | c |
| 214 | TG(18:1_38:5) | 1.8 ± 0.5 | 1.52 ± 0.29 | 2.64 ± 0.98 | 4.29 ± 2.25 | ns | ns | Increased | Increased | Increased | 0.849 | 1.467 | 2.389 | 2.815 | 1.629 | c |
| 215 | TG(18:1_38:6) | 2.28 ± 0.65 | 2.16 ± 0.65 | 2.63 ± 1.10 | 4.24 ± 1.75 | ns | ns | Increased | Increased | Increased | 0.948 | 1.155 | 1.861 | 1.962 | 1.611 | c |
| 216 | TG(18:2_30:1) | 0.49 ± 0.15 | 0.54 ± 0.16 | 0.23 ± 0.17 | 0.50 ± 0.25 | ns | ns | ns | ns | ns | 1.096 | 0.471 | 1.014 | 0.925 | 2.154 | |
| 217 | TG(18:2_32:0) | 4.36 ± 1.59 | 4.48 ± 1.81 | 9.35 ± 4.84 | 23.13 ± 10.33 | ns | ns | Increased | Increased | Increased | 1.028 | 2.147 | 5.308 | 5.162 | 2.472 | c |
| 218 | TG(18:2_32:1) | 6.27 ± 3.27 | 5.75 ± 2.52 | 3.47 ± 1.28 | 7.38 ± 4.11 | ns | ns | ns | ns | ns | 0.916 | 0.553 | 1.176 | 1.284 | 2.129 | |
| 219 | TG(18:2_32:2) | 1.90 ± 0.76 | 1.67 ± 0.92 | 2.07 ± 0.81 | 4.93 ± 2.30 | ns | ns | Increased | Increased | Increased | 0.874 | 1.085 | 2.590 | 2.962 | 2.388 | c |
| 220 | TG(18:2_33:0) | 0.38 ± 0.15 | 0.32 ± 0.23 | 0.76 ± 0.19 | 1.57 ± 0.69 | ns | ns | Increased | Increased | Increased | 0.839 | 2.010 | 4.178 | 4.977 | 2.078 | c |
| 221 | TG(18:2_33:1) | 0.92 ± 0.24 | 0.78 ± 0.18 | 1.46 ± 0.43 | 2.73 ± 1.45 | ns | ns | Increased | Increased | Increased | 0.842 | 1.576 | 2.956 | 3.508 | 1.875 | c |
| 222 | TG(18:2_34:0) | 3.18 ± 1.11 | 2.72 ± 1.25 | 6.2 ± 2.61 | 12.52 ± 6.48 | ns | ns | Increased | Increased | Increased | 0.855 | 1.953 | 3.944 | 4.612 | 2.020 | c |
| 223 | TG(18:2_34:1) | 39.37 ± 13.54 | 30.08 ± 13.97 | 52.48 ± 22.87 | 99.31 ± 50.85 | ns | ns | Increased | Increased | Increased | 0.764 | 1.333 | 2.523 | 3.302 | 1.892 | c |
| 224 | TG(18:2_34:2) | 43.05 ± 15.59 | 32.62 ± 18.44 | 82.37 ± 34.53 | 155.69 ± 72.81 | ns | ns | Increased | Increased | Increased | 0.758 | 1.913 | 3.616 | 4.772 | 1.890 | c |
| 225 | TG(18:2_34:3) | 7.45 ± 2.63 | 6.12 ± 3.42 | 11.51 ± 4.25 | 21.84 ± 10.52 | ns | ns | Increased | Increased | Increased | 0.822 | 1.546 | 2.932 | 3.566 | 1.897 | c |
| 226 | TG(18:2_35:2) | 1.03 ± 0.25 | 0.79 ± 0.52 | 2.90 ± 1.07 | 5.33 ± 2.69 | ns | ns | Increased | Increased | Increased | 0.768 | 2.822 | 5.180 | 6.747 | 1.836 | c |
| 227 | TG(18:2_36:0) | 0.36 ± 0.06 | 0.33 ± 0.3 | 0.89 ± 0.49 | 2.26 ± 1.06 | ns | ns | Increased | Increased | Increased | 0.911 | 2.436 | 6.206 | 6.809 | 2.547 | c |
| 228 | TG(18:2_36:1) | 2.63 ± 0.77 | 2.72 ± 0.96 | 8.5 ± 3.81 | 19.33 ± 9.91 | ns | ns | Increased | Increased | Increased | 1.033 | 3.233 | 7.353 | 7.116 | 2.275 | c |
| 229 | TG(18:2_36:2) | 9.34 ± 2.97 | 9.14 ± 3.09 | 31.12 ± 12.73 | 67.5 ± 33.69 | ns | ns | Increased | Increased | Increased | 0.979 | 3.332 | 7.227 | 7.384 | 2.169 | c |
| 230 | TG(18:2_36:3) | 16.32 ± 5.26 | 14.45 ± 7.84 | 62.77 ± 28.76 | 138.6 ± 59.34 | ns | Increased | Increased | Increased | Increased | 0.886 | 3.847 | 8.495 | 9.590 | 2.208 | c |
| 231 | TG(18:2_36:4) | 10.5 ± 3.29 | 9.42 ± 5.76 | 50.82 ± 27.41 | 121.96 ± 48.17 | ns | Increased | Increased | Increased | Increased | 0.897 | 4.842 | 11.620 | 12.951 | 2.400 | c |
| 232 | TG(18:2_36:5) | 2.48 ± 0.74 | 2.05 ± 1.35 | 8.61 ± 4.17 | 20.66 ± 8.32 | ns | ns | Increased | Increased | Increased | 0.828 | 3.475 | 8.336 | 10.070 | 2.399 | c |
| 233 | TG(18:2_38:5) | 2.19 ± 0.74 | 1.77 ± 0.52 | 4.31 ± 1.67 | 6.86 ± 3.04 | ns | ns | Increased | Increased | Increased | 0.810 | 1.968 | 3.134 | 3.871 | 1.593 | c |
| 234 | TG(18:2_38:6) | 3.85 ± 1.2 | 2.84 ± 1.18 | 5.88 ± 2.42 | 8.2 ± 3.25 | ns | ns | Increased | Increased | ns | 0.740 | 1.529 | 2.133 | 2.884 | 1.395 | |
| 235 | TG(18:3_34:1) | 3.59 ± 1.16 | 2.92 ± 1.43 | 5.14 ± 2.3 | 11.17 ± 5.6 | ns | ns | Increased | Increased | Increased | 0.814 | 1.432 | 3.112 | 3.823 | 2.173 | c |
| 236 | TG(18:3_34:2) | 3.35 ± 0.95 | 2.84 ± 1.56 | 5.87 ± 2.6 | 12.17 ± 5.74 | ns | ns | Increased | Increased | Increased | 0.846 | 1.751 | 3.630 | 4.290 | 2.073 | c |
| 237 | TG(18:3_36:2) | 1.19 ± 0.38 | 1.18 ± 0.33 | 3.39 ± 1.3 | 7.69 ± 3.84 | ns | ns | Increased | Increased | Increased | 0.991 | 2.846 | 6.456 | 6.518 | 2.268 | c |
| 238 | TG(18:3_36:3) | 1.98 ± 0.55 | 1.78 ± 0.92 | 6.23 ± 2.49 | 14.84 ± 6.58 | ns | ns | Increased | Increased | Increased | 0.898 | 3.145 | 7.496 | 8.345 | 2.384 | c |
| 239 | TG(18:3_36:4) | 1.25 ± 0.35 | 1.16 ± 0.81 | 4.32 ± 2.14 | 11.23 ± 4.74 | ns | ns | Increased | Increased | Increased | 0.932 | 3.464 | 9.000 | 9.656 | 2.598 | c |
| 240 | TG(20:1_34:1) | 1.26 ± 0.44 | 1.26 ± 0.51 | 0.98 ± 0.38 | 2.01 ± 1.04 | ns | ns | ns | ns | ns | 0.996 | 0.776 | 1.592 | 1.598 | 2.052 | |
| 241 | TG(20:1_34:2) | 1.52 ± 0.6 | 1.45 ± 0.51 | 1.53 ± 0.51 | 3.43 ± 1.43 | ns | ns | Increased | Increased | Increased | 0.950 | 1.006 | 2.252 | 2.371 | 2.239 | c |
| 242 | TG(20:2_34:3) | 0.34 ± 0.09 | 0.23 ± 0.15 | 0.32 ± 0.25 | 0.54 ± 0.32 | ns | ns | ns | ns | ns | 0.677 | 0.947 | 1.604 | 2.371 | 1.694 | |
| 243 | TG(20:3_34:1) | 1.09 ± 0.26 | 1.22 ± 0.5 | 0.95 ± 0.56 | 1.83 ± 1.05 | ns | ns | ns | ns | ns | 1.120 | 0.868 | 1.674 | 1.495 | 1.929 | |
| 244 | TG(20:3_34:2) | 1.49 ± 0.42 | 1.26 ± 0.39 | 1.8 ± 0.68 | 2.92 ± 1.38 | ns | ns | Increased | Increased | Increased | 0.843 | 1.211 | 1.958 | 2.323 | 1.617 | c |
| 245 | TG(20:3_36:3) | 0.88 ± 0.22 | 0.99 ± 0.25 | 1.81 ± 0.64 | 3.18 ± 1.5 | ns | ns | Increased | Increased | Increased | 1.118 | 2.050 | 3.599 | 3.220 | 1.755 | c |
| 246 | TG(20:4_34:1) | 2.88 ± 0.74 | 2.56 ± 0.58 | 2.99 ± 1.76 | 4.37 ± 2.36 | ns | ns | ns | ns | ns | 0.888 | 1.038 | 1.519 | 1.710 | 1.464 | |
| 247 | TG(20:4_34:2) | 4.06 ± 0.77 | 3.09 ± 1.04 | 5.29 ± 2.46 | 7.61 ± 3.56 | ns | ns | Increased | Increased | ns | 0.762 | 1.304 | 1.876 | 2.461 | 1.438 | |
| 248 | TG(20:4_34:3) | 1.04 ± 0.22 | 0.85 ± 0.18 | 0.87 ± 0.3 | 1.18 ± 0.43 | ns | ns | ns | ns | ns | 0.811 | 0.831 | 1.132 | 1.395 | 1.361 | |
| 249 | TG(20:4_36:2) | 1.72 ± 0.36 | 1.32 ± 0.28 | 3.03 ± 1.27 | 4.78 ± 2.32 | ns | ns | Increased | Increased | Increased | 0.767 | 1.767 | 2.785 | 3.631 | 1.576 | c |
| 250 | TG(20:4_36:3) | 2.8 ± 0.76 | 2.05 ± 0.49 | 4.88 ± 1.74 | 7.11 ± 3.04 | ns | ns | Increased | Increased | ns | 0.732 | 1.746 | 2.542 | 3.471 | 1.456 | |
| 251 | TG(20:4_36:4) | 1.94 ± 0.80 | 1.21 ± 0.59 | 3.96 ± 1.41 | 5.64 ± 2.04 | ns | Increased | Increased | Increased | ns | 0.623 | 2.042 | 2.914 | 4.675 | 1.427 | |
| 252 | TG(20:5_34:1) | 2.06 ± 0.80 | 1.86 ± 1.05 | 2.26 ± 0.99 | 2.99 ± 1.58 | ns | ns | ns | ns | ns | 0.903 | 1.095 | 1.450 | 1.605 | 1.324 | |
| 253 | TG(20:5_34:2) | 3.12 ± 1.13 | 2.69 ± 1.31 | 4.14 ± 2.19 | 5.85 ± 2.77 | ns | ns | ns | ns | ns | 0.863 | 1.328 | 1.877 | 2.173 | 1.413 | |
| 254 | TG(20:5_36:3) | 2.42 ± 0.84 | 1.85 ± 0.79 | 4.81 ± 1.56 | 6.27 ± 2.96 | ns | Increased | Increased | Increased | ns | 0.762 | 1.986 | 2.589 | 3.400 | 1.304 | |
| 255 | TG(22:2_32:4) | 0.26 ± 0.05 | 0.26 ± 0.08 | 0.38 ± 0.11 | 0.68 ± 0.17 | ns | ns | Increased | Increased | Increased | 1.011 | 1.489 | 2.630 | 2.601 | 1.766 | c |
| 256 | TG(22:5_34:2) | 2.71 ± 0.82 | 2.32 ± 0.84 | 3.09 ± 1.46 | 4.14 ± 1.77 | ns | ns | ns | ns | ns | 0.855 | 1.140 | 1.529 | 1.787 | 1.341 | |
| 257 | TG(22:6_32:0) | 0.94 ± 0.33 | 1.04 ± 0.47 | 1.3 ± 0.71 | 2.82 ± 1.32 | ns | ns | Increased | Increased | Increased | 1.112 | 1.387 | 3.017 | 2.714 | 2.176 | c |
| 258 | TG(22:6_32:1) | 2.13 ± 0.90 | 2.18 ± 0.88 | 1.07 ± 0.33 | 1.56 ± 0.63 | ns | ns | ns | ns | ns | 1.025 | 0.502 | 0.735 | 0.717 | 1.466 | |
| 259 | TG(22:6_34:1) | 10.59 ± 3.51 | 9.77 ± 3.43 | 10.45 ± 5.6 | 14.02 ± 5.44 | ns | ns | ns | ns | ns | 0.923 | 0.986 | 1.324 | 1.435 | 1.342 | |
| 260 | TG(22:6_34:2) | 15.72 ± 5.38 | 12.37 ± 4.84 | 20.15 ± 9.94 | 26.69 ± 11.49 | ns | ns | Increased | Increased | ns | 0.787 | 1.282 | 1.698 | 2.158 | 1.325 | |
| 261 | TG(22:6_34:3) | 2.86 ± 0.88 | 2.52 ± 0.86 | 2.67 ± 1.14 | 3.48 ± 1.28 | ns | ns | ns | ns | ns | 0.882 | 0.932 | 1.216 | 1.378 | 1.304 | |
